# Metabolite Profiling of a Diverse Collection of Wheat Lines Using Ultraperformance Liquid Chromatography Coupled with Time-of-Flight Mass Spectrometry

**DOI:** 10.1371/journal.pone.0044179

**Published:** 2012-08-30

**Authors:** Shawna B. Matthews, Meenakshi Santra, Meghan M. Mensack, Pamela Wolfe, Patrick F. Byrne, Henry J. Thompson

**Affiliations:** 1 Cancer Prevention Laboratory and Department of Horticulture, Colorado State University, Fort Collins, Colorado, United States of America; 2 Cell and Molecular Biology Graduate Program, Colorado State University, Fort Collins, Colorado, United States of America; 3 Department of Chemistry, Colorado State University, Fort Collins, Colorado, United States of America; 4 Colorado Biostatistical Consortium, University of Colorado, Denver, Colorado, United States of America; 5 Department of Soil and Crop Sciences, Colorado State University, Fort Collins, Colorado, United States of America; University of Hyderabad, India

## Abstract

Genetic differences among major types of wheat are well characterized; however, little is known about how these distinctions affect the small molecule profile of the wheat seed. Ethanol/water (65% v/v) extracts of seed from 45 wheat lines representing 3 genetically distinct classes, tetraploid durum (*Triticum turgidum* subspecies *durum*) (DW) and hexaploid hard and soft bread wheat (*T. aestivum* subspecies *aestivum*) (BW) were subjected to ultraperformance liquid chromatography coupled with time-of-flight mass spectrometry (UPLC-TOF-MS). Discriminant analyses distinguished DW from BW with 100% accuracy due to differences in expression of nonpolar and polar ions, with differences attributed to sterol lipids/fatty acids and phospholipids/glycerolipids, respectively. Hard versus soft BW was distinguished with 100% accuracy by polar ions, with differences attributed to heterocyclic amines and polyketides versus phospholipid ions, respectively. This work provides a foundation for identification of metabolite profiles associated with desirable agronomic and human health traits and for assessing how environmental factors impact these characteristics.

## Introduction

As a staple crop, wheat is grown on more land area worldwide than any other crop and is a close third to rice and corn in total world production [1]. In 2009, the average American consumed 178.2 lbs. of wheat products [2], making this crop an important source of dietary calories as well as fiber, micronutrients, and protein. Importantly, the types of wheat used to make major consumer products like pasta and bread are genetically distinct [3]–[5]. Contemporary wheat, though genetically diverse, originated from a natural hybridization event between *Triticum urartu* (AA genome) and *Aegilops speltoides* (SS genome (BB progenitor)) that ultimately gave rise to a tetraploid species identified as *Triticum turgidum* subsp. *dicoccoides* (2n = 4X = 28, AABB genome) [6]–[8]. This wild ancestor of wheat had two fates: cultivation leading to *Triticum turgidum* subspecies (ssp.) *durum*, today’s tetraploid pasta wheat, or further natural hybridization with *Aegilops tauschii* (DD genome) to ultimately give rise to *Triticum aestivum* ssp. *aestivum* (2n = 6X = 42, AABBDD genome), which is the progenitor of contemporary hexaploid bread wheat (BW) [6]–[8].

Three genetically distinct types of wheat that are economically important are: 1) tetraploid durum wheat (DW), 2) hexaploid hard bread wheat (HBW), and 3) hexaploid soft bread wheat (SBW), with the latter classification based on starch fractionation patterns during milling, which is heritable through chromosome 5D [6]. Each type of bread wheat, which is also referred to as common wheat and has uses in addition to making bread, is further subdivided by grain color (red vs. white), based on the number of dominant alleles at the *R/r* locus on group 3 chromosomes, and growing season (spring vs. winter), based on the dominant alleles at vernalization (*Vrn*) loci on group 5 and 7 chromosomes [6], [9]. Though these designations are globally accepted, they are predominantly used in the United States as all three types are grown domestically [6]. For the purposes of this paper, ‘class’ will refer to major market designations (DW, HBW, or SBW) while ‘subclass’ will refer to subsets of these market designations based on seed coat color and growing season, recognizing that this classification scheme differs from the official classification system used in the United States.

Breeding programs for wheat have traditionally focused on the enhancement of agronomic traits including yield, time to maturity, disease and insect resistance, and protein/gluten content and functionality [10]; this approach has been critical to establishing a plentiful and affordable food supply. However, the rapid rate of global climate change will make it difficult to sustain progress using only conventional approaches, especially in light of the expected increase in the world’s population to 9–10 billion by 2050 [11]. Moreover, in regions of the world unlikely to be affected by food shortages, the consumer is demanding foods with enhanced human health benefits.

To meet the daunting challenge of improving wheat for both agronomic and human health traits, new approaches using the advancement of microtechnologies have enabled rapid, high-throughput and affordable analyses of major classes of biologically important molecules. While the most advanced of these developments have focused on nucleic acid polymers, other microscale approaches are being applied to proteins and small molecules. The investigation of an organism’s metabolome, comprising non-protein small molecules, is a recent development in the “omics revolution” and provides a rich, real-time source of information about that organism’s functional state. However, metabolomics is arguably the least explored “omics” field, in part because the systems for extraction and analysis of small molecules have yet to be standardized, resulting in limited power to assign specific identities to detected ions similar to genomics and proteomics technologies a decade ago. Thus, the focus of the work reported in this manuscript was on metabolite profiling, which measures thousands of metabolites from cellular extracts and which seeks to characterize the systemic metabolic state of a plant, rather than metabolomics per se, which is generally considered the precise quantitation and identification of every metabolite in a sample [12] and which is currently not possible due to the infancy of plant metabolite databases.

Not surprisingly, very few reports have addressed the application of metabolite profiling analysis to wheat. The work reported herein was based on the hypothesis that genetic individuality of wheat classes confers uniqueness to metabolite profiles, enabling discrimination of tetraploid DW from genetically distinct hexaploid HBW and SBW, without controlling for environmental effects. It was also hypothesized that metabolic profiling would distinguish HBW from SBW as well as the subclasses within BW market classes. Ions with greatest discriminatory capacity in the comparisons of DW vs. BW and also HBW vs. SBW classes were identified and evaluated for trends in chemical expression patterns.

## Materials and Methods

### Plant Material

Members of the wheat improvement team at Colorado State University provided wheat seed from a diverse collection of wheat germplasm that included parents from the Wheat Coordinated Agricultural Project (http://maswheat.ucdavis.edu/Mapping/index.htm). This study was double-blinded in that the individuals providing seed were blinded to the intent of the analyses and the analytical team was blinded to class and identity of wheat seed. The growing location for the samples evaluated was not standardized, i.e. they came from a wide array of different environments. Forty-five wheat cultivars, advanced breeding lines, and germplasm representing three U.S. market classes (DW, n = 6; HBW, n = 27; and SBW, n = 12) were investigated. Bread wheat (BW) was further subdivided based on grain color and growing season into 4 subclasses: hard white winter (HWW) (n = 6), hard white spring (HWS) (n = 4), hard red winter (HRW) (n = 8), and hard red spring (HRS) (n = 9), for a total of 27 HBW lines evaluated in this study; soft white winter (SWW) (n = 6), soft white spring (SWS) (n = 2), soft red winter (SRW) (n = 4), for a total of 12 SBW lines evaluated in this study. (Note: soft red spring (SRS) wheat designation is not used within the United States wheat grain classification system.) Most lines were domestic; however, international lines from Mexico, Romania and Syria were also included. Pedigree information was collected using the Germplasm Resources Information Network (GRIN) web platform [13], and is summarized in Table 1.

**Table 1:** Pedigree information for 45 wheat lines evaluated.

Number	Wheat Line	Source	Class	Subclass	Pedigree
6	Cham1	Syria	DW	DW	Pelicano/Ruff//Gaviota/Rolette
14	Jennah Khetifa	Morocco	DW	DW	Landrace collected from the Atlas Mountains of Morocco
18	Kofa	California, USA	DW	DW	Selection from composite cross *T. dicoccon* alpha-85 S-1
21	Maier	North Dakota, USA	DW	DW	D8193/D8335
37	Rugby	North Dakota, USA	DW	DW	Langdon/5/(Heiti/Stewart//Mindum/Carleton, Ld 357 )/4/CI 7780/Ld 362/6/Br 180/Wells
44	UC1113	California, USA	DW	DW	Selection from cross CD52600 Kingfisher 'S/Roussia/BD1419/3/Mexi 'S - CP/4/Waha 'S/5/Yavaros 79
9	Conan	Montana, USA	HBW	HRS	Rambo/Westbred 906R
11	Grandin	North Dakota, USA	HBW	HRS	Len//Butte*2/ND507/3/ND593
16	Jupateco 73S	Mexico	HBW	HRS	NIL selection from Jupateco 73 (II12300/Lerma Rojo 64/8156/3/Norteno 67 = II30842)
22	McNeal	Montana, USA	HBW	HRS	PI 125000/Centana//PK176/Frontiera/3/Glenman
23	MTRWA116	Montana, USA	HBW	HRS	PI372129/2*Pondera
31	PI610750	Mexico	HBW	HRS	Crocl/*Ae. tauschii* (205)//Kauz
34	Reeder	North Dakota, USA	HBW	HRS	IAS20*4/H567.71//Stoa/3/ND674
39	Steele	North Dakota, USA	HBW	HRS	Parshall’ (PI 613587)/ND706
42	Thatcher	Minnesota, USA	HBW	HRS	Marquis/Iumillo//Marquis/Kanred
1	2174	Oklahoma, USA	HBW	HRW	IL71-5662/‘PL145’ (PI 600840)//‘2165’
2	Ankor	Colorado, USA	HBW	HRW	Akron/Halt//4*Akron
10	Flamura 85	Romania	HBW	HRW	Mixture (in equal proportions) of 5 lines produced by backcrossing Flamura 80 for resistance to powdery mildew [*Erysiphe. graminis*]
12	Hatcher	Colorado, USA	HBW	HRW	Yuma/PI 372129//TAM-200/3/4*Yuma/4/KS91H184/Vista
13	IDO444	Idaho, USA	HBW	HRW	Utah 216c-12 - 10/Cheyenne/5/PI 476212/4/Burt/3/Rio/Rex//Nebred//6//Utah 216c-12 - 10/Cheyenne/5/PI 476212/4/Burt/3/Rio/Rex//Nebred
15	Jagger	Kansas, USA	HBW	HRW	KS82W418/Stephens
20	Lovrin 34	Romania	HBW	HRW	Raniniaja 12/Nadadores 63//Lovrin 12
41	TAM107-R7	Colorado, USA	HBW	HRW	CO850034/PI372129//5*TAM107
45	Weebill 1	Mexico	HBW	HWS	Babax/Amadina//Babax
17	Kauz	Mexico	HBW	HWS	Jupateco F73/Bluejay//Ures T81
36	Sokoll	Mexico	HBW	HWS	Pastor/3/Altar84/*Ae. tauschii*//Opata
43	UC1110	California, USA	HBW	HWS	Nord Desprez/Pullman Sel. 101, CI 13438
3	Arlin	Kansas, USA	HBW	HWW	Selection from population of intercrossed HRW wheat and HRS wheat genotypes
7	CO940610	Colorado, USA	HBW	HWW	KS87H22/MW09 (KS87H22 = H15A13333/5*Larned//Eagle/Sage/3/TAM 105, MW09 = Clark's Cream/5*KS75216 (Newton Sib))
24	ND735	North Dakota, USA	HBW	HWW	ND 2907/3/Grandin*3//Ramsey/ND 622/4/ND 2809
33	Platte	Colorado, USA	HBW	HWW	N84-1104/Abilene (OK11252/W76-1226)
35	Rio Blanco	Kansas, USA	HBW	HWW	OK11252A/W76-1226
26	NY18/Clarks Cream 40-1	New York, USA	HBW	HWW	N/A
28	P91193	Indiana, USA	SBW	SRW	Benhur//Arthur/Knox62/3/Arthur/NY5715AB/4/Hart/Beau/9/Arthur/8/Afghanistan/Knox*4/6/Kawvale 3/Fultz/Hungarian//W381/4/Wabash/3/Fairfield/Trumbull//G2343/5/Knox/4/Fairfield/3/PI94587//Fultz/Hungarian/7/Redcoat/6/Norin33/5/Fairfield/3/PI94587//Fultz/Hungarian/4/Knox/10/Auburn/Coker8427/3/OH256/Scotty//Clark
29	P92201	Indiana, USA	SBW	SRW	Tyler//Caldwell *2/S76/3/Clark/4/CI15549/5/Caldwell*2/Roazon//Glory
32	Pioneer Variety 26R46	Georgia, USA	SBW	SRW	FL7927-G14//2555*3/Coker 80-28
38	SS550	Virginia, USA	SBW	SRW	Coker 9803/Freedom
19	Louise	Washington, USA	SBW	SWS	Wakanz/Wawawai
30	Penawawa	Washington, USA	SBW	SWS	Potam 70/Fielder
4	Brundage	Idaho, USA	SBW	SWW	Stephens/Geneva
5	Caledonia	New York, USA	SBW	SWW	Variant in Geneva
8	Coda	Idaho, USA	SBW	SWW	Tres/Madsen//Tres
25	NY Cayuga	New York, USA	SBW	SWW	Geneva/Clark's Cream//Geneva
27	OR9900553	Oregon, USA	SBW	SWW	Arminda/3/VPM/MOS951//2*Hill/5/ID#870337
40	Stephens	Oregon, USA	SBW	SWW	Nord Desprez/Pullman Sel. 101, CI 13438

Table columns: Numbers = identifiers used within manuscript to visualize chemical separations in scatter plots and dendrograms; Wheat Line = common field identifier; Source = geographical location where grown; Class = 1 of 3 market classes: durum (DW), hard bread wheat (HBW), or soft bread wheat (SBW); Subclass = subclass within bread wheat market classes based on seed coat color and growth habit (HWW = hard white winter; HWS = hard white spring; HRW = hard red winter; HRS = hard red spring; SWW = soft white winter; SWS = soft white spring; SRW = soft red winter); Pedigree = wheat line development and breeding.

### Metabolite Extraction

Ethanol (65% v/v), sonicator, and a refrigerated centrifuge were used for metabolite extraction. Extraction was carried out using ultrasound assisted extraction (UAE), which both accelerates and facilitates extraction of organic and inorganic compounds as reviewed in [14]. Ten mL of ethanol (65%) was added to 0.5 g milled, uncooked wheat seed, in triplicate for each line and the mixture sonicated for 2 h at room temperature (22±2°C). This was followed by a centrifugation step (1008×*g*, 4°C, 10 min) to remove insoluble material. Extracts were then decanted into fresh 50 mL conical tubes and aliquots transferred to separate vials for analysis. The remaining solution was placed in a freezer (−20°C) for storage (up to one month).

### Metabolite Analysis by UPLC-TOF-MS

An Acquity UPLC controlled with MassLynx software, version 4.1 (Waters, Milford, MA) was used for sample analysis, in which 45 wheat extracts were randomized and analyzed in three independent iterations based on our previously described analytical protocol [15]. Briefly, dried extracts were resuspended in eluent and held at 10°C in a sample manager during the analysis to prevent evaporation prior to UPLC-TOF-MS analysis. For sample separation, an Acquity UPLC held at 40°C with a 1.0×100 mm Waters Acquity UPLC was used, with 1.7 µm Ethylene Bridged Hybrid (BEH)-C18 particles. One µL sample injections were made from 100 µL total sample volumes. Reversed phase chromatography at a flow rate of 0.14 mL·min-1 was used for separation, with eluent compositions of 95∶5 water: methanol (solvent A) (LC-MS grade, Thermo Fisher, San Jose, CA) and 100% methanol (solvent B) (LC-MS) grade, Fluka, St. Louis, MO) both with 0.1% (v/v) formic acid. Separation was achieved by 58 min method as follows: 3 min hold at 100% A, 30 min linear gradient to 100% B, 12 min hold at 100% B, 3 min linear gradient to 100% A, and 10 min hold at 100% A for equilibration. A Q-Tof Micro hybrid quadrupole, orthogonal acceleration time-of-flight mass spectrometer (Waters/MicroMass) using positive mode electrospray ionization (ESI+), was used to collect mass spectral data at a rate of two scans per second over a mass to charge (m/z) range 50–1500 Da.

UPLC-TOF-MS parameters were set as follows: capillary = 3000 V; cone nitrogen flow = 50 L/h; sample cone = 30 V; extraction cone = 2.0 V; desolvation temperature = 250°C; desolvation flow = 400 L/h; source temperature = 130°C. Leucine enkephalin was used as a lock mass reference to ensure accurate mass measurements within 7 ppm. The lock mass compound was introduced via a separate orthogonal ESI+ spray and baffle system (LockSpray) for detection of ions for 0.5 s every 10 s in an independent data collection channel. The standard mass was averaged across 10 scans providing a continuous reference for mass correction of analyte data. Mass spectral scans were mean-centered in real time using MassLynx software.

### Peak Detection, Deconvolution, Filtering, and Scaling

Each wheat line was analyzed in triplicate, resulting in a total of 9 technical replicates per biological replicate (e.g., each wheat line). Mean-centered and integrated peaks were detected, extracted, and aligned using MarkerLynx software (Waters). Chromatographic peaks were extracted from 0 to 35 min with a retention time error window of 0.1 min and mass spectral peaks detected from 50 to 1500 m/z with a mass error window of 7 ppm, generating a data matrix consisting of retention time, m/z, and peak intensity based on peak area for all features.

### High Quality Ion List

A total of 3727 chemical features were detected from UPLC-TOF-MS yielding a preliminary dataset with 405 rows and 3727 features (columns). Initial data reduction was achieved by averaging each wheat line over technical replicates, followed by 2 filtering steps to ensure high quality ions. The first filtering step used a “≥80% present” cut-off criterion within at least one class. For example, if ≥80% DW lines had non-zero intensity values for a specific feature, that feature was retained irrespective of whether SBW or HBW fulfilled the same criteria for that ion. This step removed 2355 ions as noise, or 63.1% of the original dataset. The second filtering step used a ≥1.0 cut-off criterion for averaged intensity values across all classes. For example, if the averaged intensity value of all 45 wheat lines for a specific feature was ≥1.0, the feature was retained. The second filtering step removed an additional 437 ions, for total removal of 2792 ions (75%), leaving a high quality list containing 935 ions for subsequent analysis. Data were normalized with Pareto scaling (scaling factor = standard deviation) before statistical evaluation.

### Statistical Analysis

Supervised and unsupervised multivariate techniques were employed to evaluate and visualize the data [16]–[19].

### PCA (Unsupervised)

Interpretation of multivariate analysis was recently described by our laboratory [20]. Principal components analysis (PCA) summarizes a set of correlated variables by transforming them, by means of an eigen decomposition, into a new set of uncorrelated variables, reducing the dimensionality of the original high-dimensional dataset, and is carried out with no prior knowledge of class membership. The first principal component (PC) is the linear combination of the features (935 ions) that passes through the centroid of the full dataset while minimizing the square of the perpendicular distance of each point to that line; each subsequent PC is constructed in a similar manner while being mutually orthogonal [21]. The PCA model is written:
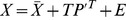
(1)where *X* is the matrix of 935 ions, 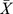 is a vector of means (all 0 when the data are centered), *T* is a matrix of scores that summarize the *X* variables, 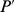 is a matrix of loadings, superscript *^T^* denotes matrix transposition, and *E* is a matrix of residuals.

### OPLS-DA (Supervised)

Orthogonal projections to latent structures for discriminant analysis (OPLD-DA) is a supervised, class-based method where class membership is assigned to samples and used to elicit maximum data separation [17]–[19], [22], and is written:
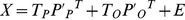
(2)where the interpretation of equation 2 is similar to that for the PCA model, however, an additional rotation has been applied using the class information to partition 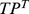 into predictive, 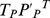, and orthogonal, 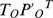, components. The number of predictive and orthogonal components in the models was determined by 7-fold cross-validation. Three key statistics, which are summarized in Table S1, are required to describe the fit of each model. First, R2X(cum) is the total amount of explained variation in X; R2Y(cum) is the total amount of variation explained in Y; and third, Q2Y(cum) is the total amount of predicted variability in Y, estimated by 7-fold cross validation. The contribution of each component partitioned into between-class (predictive) and within-class (orthogonal) variance is also estimated, and summarized as R2X_p_ and R2X_o_, respectively, with number of components denoted as subscripts (e.g. R2X_p1–2_ for a model with two predictive principal components). R2X_p_ and R2X_o_ sum to R2X(cum). The ability of the model to classify the observations into the defined classes is reflected in misclassification rates for each model, where wheat lines were classified based on the modeled probability of a single observation belonging to a particular class.

### Visualization of PCA and OPLS-DA

Scatter plots of the first two score vectors for the PCA models were drawn, along with 95% confidence ellipses based on Hotelling’s multivariate T^2^, to identify outliers that might bias the results of OPLS-DA. For OPLS-DA, class separation was shown in several ways. The first predictive score was plotted against the first orthogonal score to visualize the within- and between-class variability associated with the first principal component, and dendrograms were drawn using the first (or first and second) predictive scores, by the single linkage method, and sorted by size. In the single linkage method, observations were merged by proximity to neighbors based on Euclidean distance, building the hierarchy from individual observations by progressively merging clusters until all observations are merged into one parent cluster. The resulting clusters are further characterized by compactness and distinctness. Compactness is the Euclidean distance of the cluster node from 0; the smaller the number, the more chemically similar are the elements in the cluster [23]. Similarly, distinctness is the Euclidean distance from the cluster node to the next highest cluster; the larger the number, the more chemically distinct the clusters are from each other [23].

Finally, S-plots were constructed to identify influential ions in the separation of tetraploid DW from hexaploid BW and SBW from HBW. S-plots based on the first principal component show reliability (modeled correlation) plotted against feature magnitude (loadings or modeled covariance). If ions have variation in correlation and covariance between classes, this plot will assume an S-shape (giving the plot its name), with heavily influential features separating from other features at the upper right and lower left tails of the feature cloud within the model space [18], [19]. From these S-plots, ions with high discriminatory capacity, that is, with high likelihood for potential biomarkers, were manually chosen based on their physical separation (high magnitude and high reliability), as well as statistical significance [19]. This approach ensures that influential ions are not chosen solely on the basis of high spectral intensity or magnitude (high covariance) nor chosen solely on the basis of high X−Y correlation, in which case an abundance of low-intensity ions with high correlation would increase the false positive rate. All analyses were done using SIMCA-P+ v.12.0.1 (Umetrics, Umea, Sweden).

### Identification of Chemical Features

METLIN: Metabolite and Tandem MS Database (http://metlin.scripps.edu/) was used to assign tentative compound identities and empirical formulas [24]. Upon entering m/z of influential ions chosen from the S-plot, METLIN queried the database with expanded lipids with maximal error set at 10 ppm under the following positive ionization adduct scan modes: parent ion mass (M)+H; M+Na; M+H−2H_2_0; M+H−H_2_0; M+K; M+2Na−H; M+2H; M+3H; M+H+Na; M+2H+Na; M+2Na; M+2Na+H; M+Li; and M+CH_3_OH+H.

Note: The step-wise workflow for the metabolite profiling described above is depicted in Figure S1.

## Results

### Between-class Discrimination

**Figure 1:**
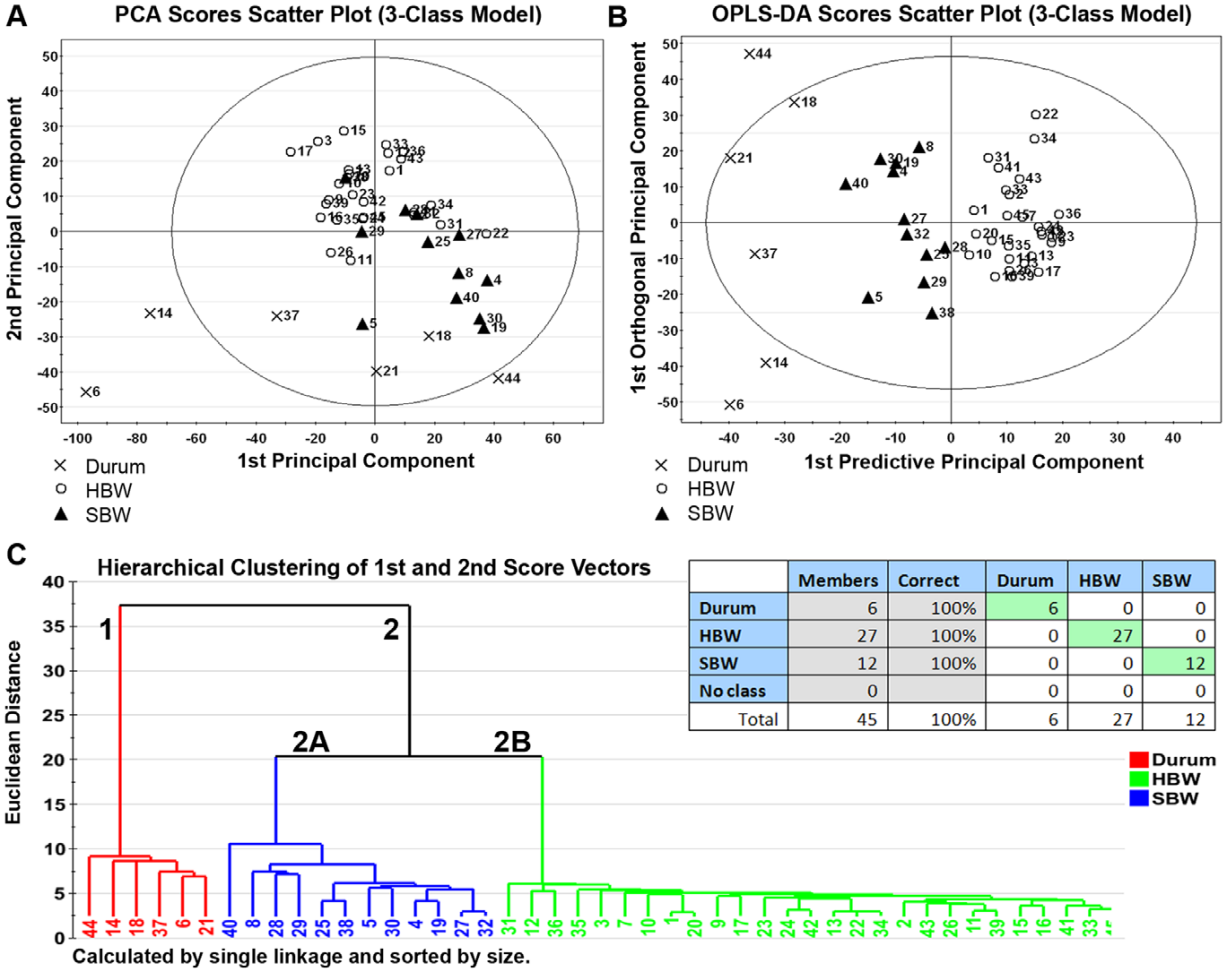
Metabolite profiling distinguishes between genetically distinct wheat classes with 100% accuracy. Multivariate discriminant analysis of the high-quality ion list, consisting of 935 ions in 45 wheat lines, was used to distinguish between wheat classes of differing ploidy levels: tetraploid durum wheat (DW) vs. hexaploid bread wheat, which comprises hard (HBW) and soft (SBW) bread wheat. Each point represents a single observation (e.g. each wheat line). **(Panel 1A)** To visualize inherent clustering patterns, the scatter plot represents unsupervised analysis through the PCA 3-class model. Separation of DW lines from HBW and SBW lines is observed. Model fit: R2X(cum) = 68.6%, with 7 components, and Q2(cum) = 38.9%. **(Panel 1B)** To determine contributing sources of variation, the scatter plot represents supervised analysis of the 3-class OPLS-DA model, which rotates the model plane to maximize separation due to class assignment. Near-complete separation of the 3 classes was observed. Model fit: R2Y(cum) = 93.2%, Q2Y(cum) = 71.0%. **(Panel 1C-Inset)** The misclassification table for the 3-class OPLS-DA model indicates that 100% of wheat lines (45 of 45 lines) were correctly classified, with low probability (p = 3.10E−17) of random table generation as assessed by Fisher’s Exact Probability. **(Panel 1C)** To visualize the misclassification rate, the dendrogram depicts hierarchical clustering patterns among major wheat classes using single linkage and size. Two main clusters comprise 1) DW lines and 2) all BW lines, with cluster 2 branching into 2A, comprising SBW lines, and 2B, comprising HBW lines. Node height of cluster 1 from 0 confirms the high degree of chemical distinctness seen within the DW lines evaluated in this study compared to node height of cluster 2.

To determine if genetic individuality of wheat classes confers chemical distinctness, the high quality ion list was first evaluated using PCA on all 3 classes of wheat. PCA identified 7 significant components that explained a total of 68.6% of the variance in the high quality ion list. The first 2 component scores of the model are shown in Figure 1A. Hexaploid hard (HBW) and soft (SBW) lines separated well from tetraploid durum (DW) lines; the scatter of DW lines relative to the scatter of BW lines was indicative of increased chemical diversity within the durum lines evaluated. Three of the 6 DW lines, 14, 44, and 6, fell outside the 95% confidence ellipse.

OPLS-DA was then used to refine the model fit and partition the variance into predictive (ion differences related to wheat class) and orthogonal (ion differences unrelated to wheat class) sources. The first predictive and orthogonal components are plotted in Figure 1B; 11.8% of the variance in ion type and concentration was related to wheat class (first of 2 predictive principal components), whereas 21.4% of the variance was unrelated to wheat class (first of 4 orthogonal principal components). HBW and SBW lines clustered around 0 on the y-axis, with DW lines having the widest scatter. All 45 wheat lines were correctly classified and the overall fit of the model was excellent (R2X_P1,2_ = 22.0%, R2X_O1–4_ = 37.7%, R2Y(cum) = 93.2%, Q2(cum) = 71.0%).

The dendrogram in Figure 1C, constructed using the first 2 score vectors form the OPLS-DA model, illustrates the classification accuracy. Two main clusters were defined: 1) DW lines and 2) BW lines, which subsequently splits into clusters comprising 2A) SBW and 2B) HBW lines. The dendrogram indicates that cluster 1 (DW) is more compact (compactness = 7.2) and therefore durum lines are more chemically similar than lines within cluster 2 (BW) (compactness = 19.2). However, within cluster 2, HBW lines in cluster 2B (compactness = 3.9) are more chemically similar than are SBW lines in cluster 2A (compactness = 8.7). Similarly, DW lines forming cluster 1 are chemically distinct from the BW lines within cluster 2, based on vertical distance to the parent cluster (distinctness = 18.1 and 30.2 for DW and BW, respectively). Chemically comparable wheat lines can be identified based on the hierarchical distance from 0 at which they cluster; HBW lines 1 and 20 were the first to cluster and thus are the most chemically similar lines within the dataset, followed by the addition of HBW line 10 and so on until all lines converge in a single primary cluster.

### Within-class Discrimination

Hexaploid HBW and SBW are classified into subclasses based on grain color and growing season [25]. The compactness and lower diversity of the HBW cluster (2B) compared to SBW (2A) observed in the 3-class dendrogram in Figure 1C, offers a test of the capacity of metabolite profiling to distinguish among subclasses of the same ploidy level.

#### Hard bread wheat

**Figure 2:**
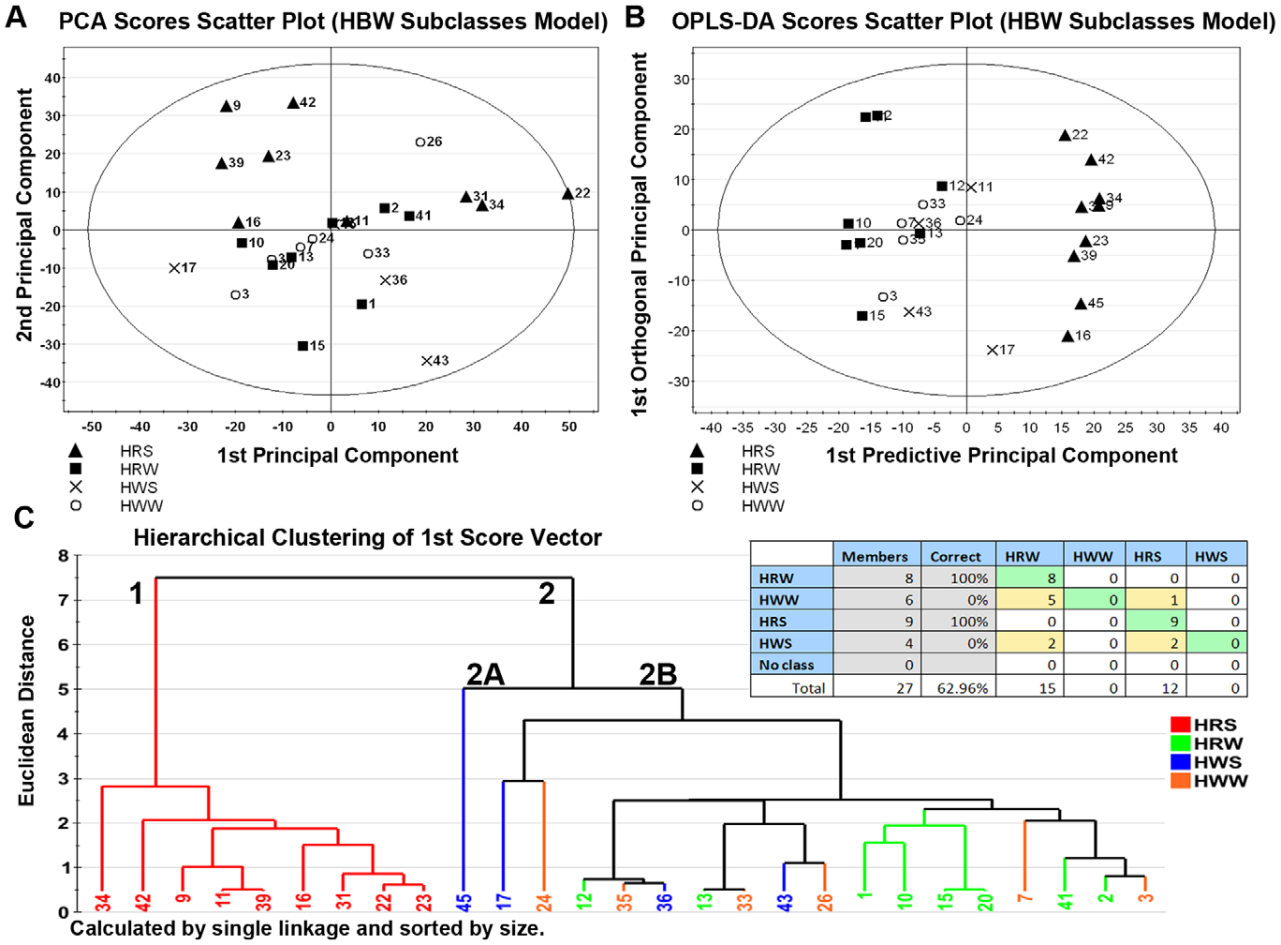
Metabolite profiling distinguishes between HBW subclasses with >62% accuracy. Multivariate discriminant analysis of the high-quality ion list, consisting of 935 ions in 27 wheat lines, was used to distinguish between subclasses of hard bread wheat (HBW) comprising hard white winter (HWW), hard white spring (HWS), hard red winter (HRW), and hard red spring (HRS). **(Panel 2A)** To visualize inherent clustering patterns, the scatter plot depicts unsupervised analysis through the PCA model. Model fit: R2X(cum) = 40.3%, with 3 components, and Q2(cum) = 10.8%. **(Panel 2B)** To determine contributing sources of variation, the scatter plot represents supervised analysis of the OPLS-DA model. Near-complete separation of subclasses was observed. Model fit: R2Y(cum) = 36.6%, Q2Y(cum) = 17.3%. **(Panel 2C-Inset)** The misclassification table for the OPLS-DA model indicates that approximately 63% of wheat lines (17 out of 27 lines) were correctly classified, with low probability (p = 1.40E−05) of random table generation as assessed by Fisher’s Exact Probability. **(Panel 2C)** To visualize the misclassification rate, the dendrogram was constructed using single linkage hierarchical clustering and sorted by size. Two main clusters comprise 1) HRS and 2) the other 3 subclasses, which do not cluster by subclass, indicating a high degree of chemical homogeneity and therefore resistance to clustering by hierarchical methods between HBW subclasses.

A total of 27 HBW lines comprising data from the high quality ion list were evaluated by PCA, for which the first 2 scores vectors are plotted in Figure 2A. Overall, the model consisted of 3 principal components that explained 40.3% of the variance within the high quality ion list, and which resulted in a relatively poor separation of the 4 subclasses.

An OPLS-DA model for HBW with the 4 subclasses for color and growing season coded as the Y variable, produced the scores plotted in Figure 2B. The first predictive principal component explained 9.7% of the variability in the ion set, while 11.4% was explained by the first orthogonal principal component. In addition to the improved separation of subclasses, Figure 2B demonstrates that the hard red spring (HRS), hard red winter (HRW), and hard white winter (HWW) subclasses have approximately equal scatter around 0 on the vertical axis and thus similar amounts of variation due to orthogonal sources of the HBW lines evaluated herein. Ten of the 27 lines were misclassified (63% classified correctly) and the overall fit of the model was poor (R2X_P1_ = 9.7%, R2X_O1_ = 11.4%, R2Y(cum) = 36.6%, Q2(cum) = 17.3%).

The dendrogram constructed from this model is shown in Figure 2C. There are two main clusters comprising 1) the HRS subclass and 2) the other 3 subclasses. While cluster 1, comprising HRS lines, is chemically distinct (compactness = 2.5, distinctness = 5.0) from the other subclasses with 100% classification accuracy, the remaining subclasses do not readily cluster using hierarchical clustering methods.

#### Soft bread wheat

**Figure 3:**
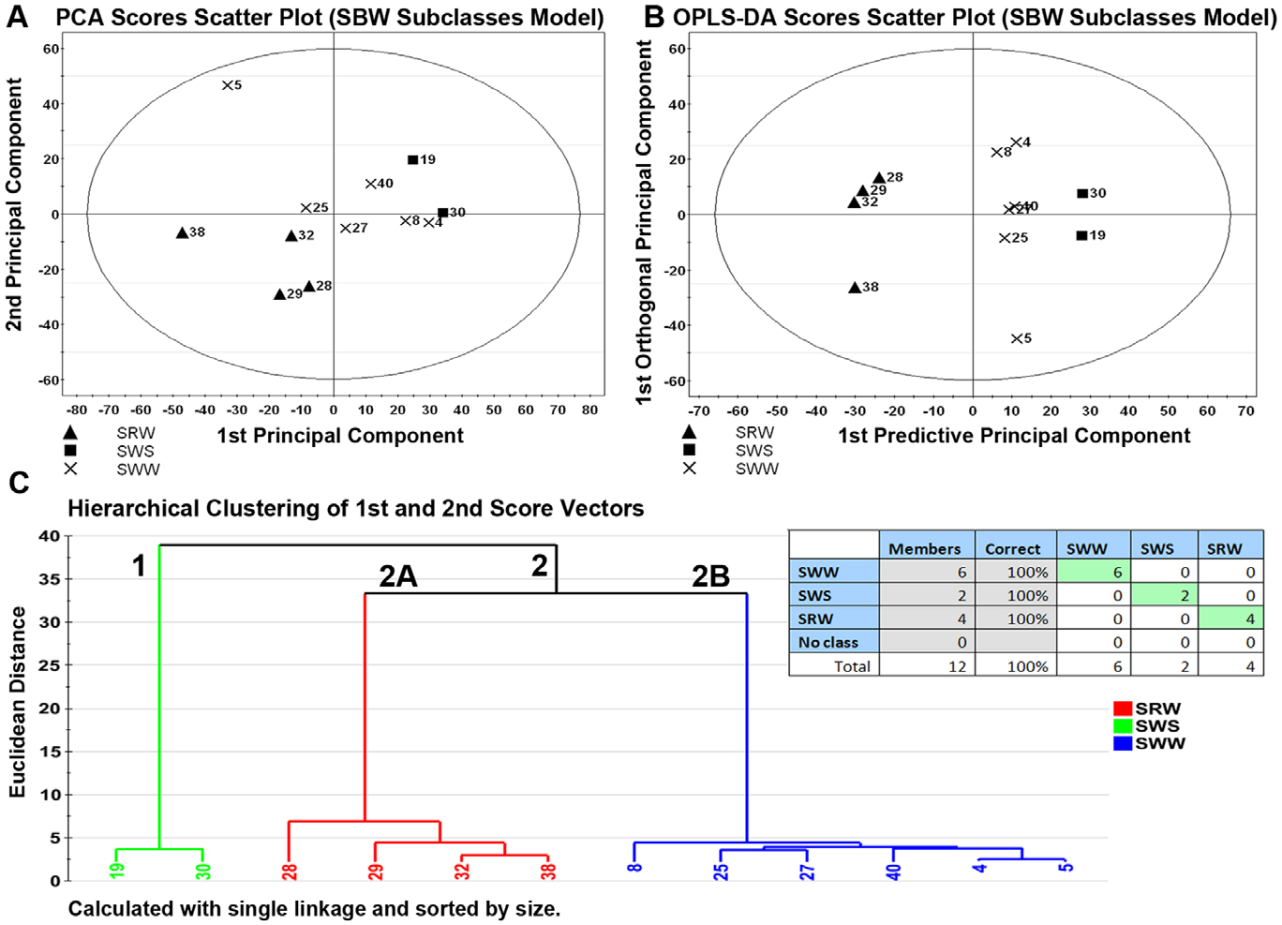
Metabolite profiling distinguishes between SBW subclasses with 100% accuracy. Multivariate discriminant analysis of the high-quality ion list, consisting of 935 ions in 12 wheat lines, was used to distinguish between subclasses of soft white winter (SWW) comprising soft white winter (SWW), soft red winter (SRW), and soft white spring (SWS). **(Panel 3A)** To visualize inherent clustering patterns, the scatter plot represents unsupervised analysis through the PCA model. Model fit: R2X(cum) = 48.9%, with 2 components, and Q2(cum) = 4.9%. **(Panel 3B)** To determine contributing sources of variation, the scatter plot represents supervised analysis of the OPLS-DA model. Subclasses demonstrate complete separation, and the propensity of wheat lines to localize near lines of similar growth habit, as observed with hard bread wheat lines, was observed in soft bread wheat lines: the divergence of SRW and SWW from a common parent cluster indicates chemical similarity. Model fit: R2Y(cum) = 99.1%, Q2Y(cum) = 64.9%. **(Panel 3C-Inset)** The misclassification table for the OPLS-DA model indicates that 100% of wheat lines (12 out of 12 lines) were correctly classified, with low probability (p = 7.20E−05) of random table generation as assessed by Fisher’s Exact Probability. **(Panel 3C)** To visualize the misclassification rate, the dendrogram was constructed using single linkage hierarchical clustering and sorted by size. Two main clusters comprise 1) SWS and 2) the 2 winter habit subclasses, with cluster 2 branching into 2A, comprising SRW lines, and 2B, comprising SWW lines, suggesting that SBW subclasses have unique chemical profiles.

Twelve SBW lines were evaluated using the PCA model, shown in Figure 3A with the first 2 score vectors plotted. Overall, the model consisted of 2 predictive components that explained a total of 48.9% of the variance within the high quality ion list, and which resulted in complete separation of the 4 subclasses on the first score vector.

An OPLS-DA model was next constructed on the 3 SBW subclasses. The first of 2 predictive principal components explained 22.7% of variance in the ion set related to the classes, and the first of 3 orthogonal principal components explained 23.0% of the variance in the ion set unrelated to Y. There were no misclassifications, and while the overall fit of the model was excellent, predictability was relatively poor due to the small sample size (R2X_P1,2_ = 32.1%, R2X_O1–3_ = 40.0%, R2Y(cum) = 99.1%, Q2(cum) = 64.9%).

The dendrogram constructed from this analysis, shown in Figure 3C, shows two main clusters comprising 1) the SWS subclass and 2) the other 2 subclasses, which splits into 2A) SWW and 2B) SRW subclasses. Each cluster, comprising one subclass, is characterized by homogeneity and is highly chemically distinct from the other subclasses (compactness = 1.5, 4.9, and 2.3; distinctness = 37.5, 34.1, and 36.7 for SWS, SWW, and SRW, respectively).

### Tentative Identification of Influential Ions

**Figure 4:**
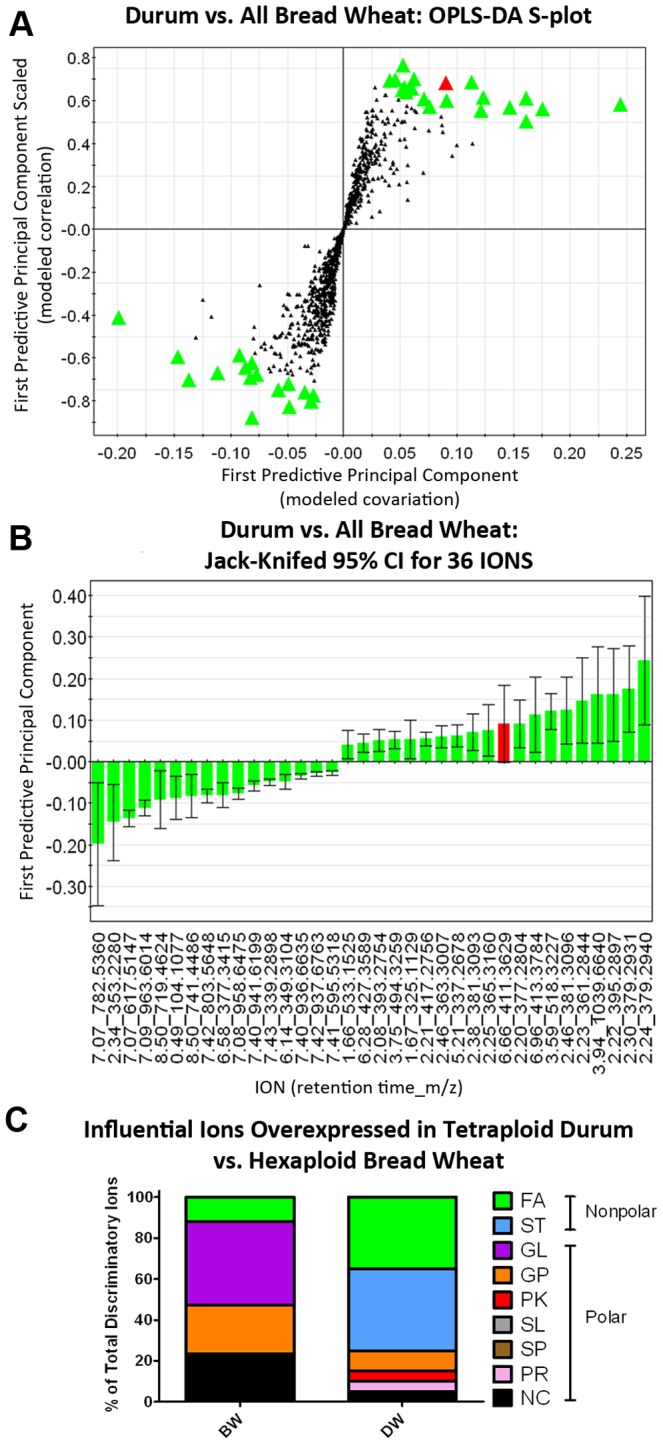
Discriminatory ions of differential polarity determine separation of tetraploid DW from hexaploid BW lines. Multivariate analysis was extended to identify influential ions responsible for the separation between classes. **(Panel 4A)** The supervised OPLS-DA model was created to compare all durum lines to all bread wheat lines, and an S-plot was constructed by plotting modeled correlation in the first predictive principal component against modeled correlation from the first predictive component (t1). Upper right and lower left regions of S-plots contain candidate biomarkers with both high reliability and high magnitude; discriminatory ions (n = 36) chosen from these regions are enlarged X3. **(Panel 4B)** To determine the statistical reliability of the ions chosen in **Panel 4A**, jack-knifed confidence intervals (JKCI) were created on the magnitude of covariance in the first component for the 36 ions and sorted in ascending order based on expression in durum wheat; ions with JKCI including 0 were excluded from further analysis (indicated by red bars in **Panel 4B** and red icons in **Panel 4A**), resulting in n = 35 ions responsible for the separation of durum wheat from bread wheat. **(Panel 4C)** Of the 31 ions to which tentative compound identities and empirical formulas could be assigned by the METLIN: Metabolite and Tandem MS Database, the tentative identity with the smallest accurate mass error was assigned to 1 of 8 lipid classes according to the Lipid Maps classification system: 1) fatty acyls (FA), 2) glycerolipids (GL), 3) glycerophospholipids (GP), 4) sphingolipids (SP), 5) sterol lipids (ST), 6) prenol lipids (PR), 7) saccharolipids (SL), 8) polyketides (PK), or NC for the 4 ions in which tentative identities could not be matched to the ion m/z. Of the 19 ions overexpressed in durum wheat (DW) compared to bread wheat (BW), 62.5% were tentatively identified as nonpolar lipids, while 74% of the 16 ions overexpressed in BW were tentatively identified as polar lipids, suggesting differences in lipid biosynthetic pathways within the two species.

To determine the identity of ions responsible for the unique chemical profiles of wheat classes, a two-class OPLS-DA model comparing tetraploid durum to all hexaploid BW lines (HBW + SBW) was used to generate the S-plot shown in Figure 4A. From the S-plot, discriminatory ions were manually chosen based on their physical separation from the main body from regions described by Wiklund due to both high reliability and high magnitude [19]. Use of both parameters favors identification of influential ions with concomitantly high magnitude (covariance) and high reliability (correlation) [18], [19]; ions found in the upper right and lower left corners were overexpressed or underexpressed, respectively, in DW compared to BW. The performance of the model parameterized in this way was excellent, with R2Y(cum) = 95.4% and QY(cum) = 82.1%.

As indicated in Figure 4A, 36 ions (enlarged icons) were selected for high discriminatory capacity. Figure 4B shows the 95% confidence intervals for covariance of the 36 discriminatory ions in the first principal component sorted in ascending order, which relegates ions with elevated expression in DW compared to BW to the distal end of the x-axis. These jack-knifed confidence intervals (JKCI) identified ions with high reliability (green bars) versus low reliability (red bar). Ions with low reliability, based on error bars crossing 0, were excluded from further analysis. Together, Figures 4A and 4B revealed a total of 35 statistically significant ions responsible for separation of tetraploid DW from hexaploid BW.

These 35 ions were submitted to batch analysis using the METLIN: Metabolite and Tandem MS Database from the Scripps Institute using 10 ppm error and correcting for positive ionization adducts [24]. “Best-choice” tentative identities for each ion were chosen based on smallest accurate mass error (AME); in the event of an AME tie, adducts with equal AME but different empirical formulas were reported. Tentative identities, retention time, adduct, and tentative empirical formula for discriminatory ions in the separation of DW from BW are reported in Table 2. As most ions had multiple isomers and derivatives within a single empirical formula and exact mass as provided by METLIN, in the interest of simplicity, only general tentative identities and classes for each ion are reported in Table 2; all stereoisomers and tentative adducts of the 35 ions are provided in Table S2.

**Table 2:** Tentative identities of ions with high discriminatory capacity in the durum vs. bread wheat OPLS-DA model.

Ion Identifier	Ion RT	Ion m/z	Adduct	Adduct Mass	Dppm	Tentative Identity	Empirical Formula	Class	Overexpressed In:	Broad Class
0.49_104.1077	0.49	104.1077	[M+H+Na]2+	184.2191	0	Tridecane	C13H28	FA	BW	NP
7.43_339.2898	7.43	339.2898	[M+H−H2O]+	356.2926	0	Heneicosanedioic acid, 6 MG derivatives	C21H40O4	FA	BW	NP
6.14_349.3104	6.14	349.3104	[M+3H]3+	1044.9085	0	7 TG derivatives	C69H120O6	GL	BW	PL
2.34_353.228	2.34	353.2280	[M+2Na]2+	660.4754	3	3 DG derivatives	C43H64O5	GL	BW	PL
6.58_377.3415	6.58	377.3415	[M+H−2H2O]+	412.3553	2	2 MG derivatives	C25H48O4	GL	BW	PL
7.41_595.5318	7.41	595.5318	[M+H]+	594.5223	3	12 DG derivatives	C37H70O5	GL	BW	PL
7.07_617.5147	7.07	617.5147	[M+H]+	616.5067	1	21 DG derivatives	C39H68O5	GL	BW	PL
7.42_937.6763	7.42	937.6763	[M+K]+	898.7050	8	8 TG derivatives	C59H94O6	GL	BW	PL
8.50_719.4624	8.50	719.4624	[M+Na]+	696.4730	0	2 PA derivatives	C39H69O8P	GP	BW	PL
8.50_741.4486	8.50	741.4486	[M+2Na−H]+	696.4730	6	2 PA derivatives	C39H69O8P	GP	BW	PL
7.07_782.536	7.07	782.5360	[M+CH3OH+H]+	749.4996	3	3 PC derivatives, 2 PE derivatives	C42H72NO8P	GP	BW	PL
7.42_803.5648	7.42	803.5648	[M+H]+	802.5598	2	PG(17∶0/20∶4(5Z,8Z,11Z,14Z))	C43H81NO10P	GP	BW	PL
7.40_936.6635	7.40	936.6635	NC					NC	BW	
7.40_941.6199	7.40	941.6199	NC					NC	BW	
7.08_958.6475	7.08	958.6475	NC					NC	BW	
7.09_963.6014	7.09	963.6014	NC					NC	BW	
1.67_325.1129	1.67	325.1129	[M+H+Na]2+	626.2296	0	Tetrahydropteroyltri-L-glutamate	C24H34N8O12	FA	DW	NP
2.25_365.316	2.25	365.3160	[M+K]+	326.3549	5	Behenyl alcohol	C22H46O	FA	DW	NP
2.38_381.3093	2.38	381.3093	[M+Na]+	358.3236	9	3,7,11,15,19-Pentamethyleicosa-2E,6E,10E,14E,18-pentaen-1-ol; 15-methyl-1,2-heneicosanediol	C25H42O	FA	DW	NP
2.46_381.3096	2.46	381.3096	[M+Na]+	358.3236	8	3,7,11,15,19-Pentamethyleicosa-2E,6E,10E,14E,18-pentaen-1-ol; 15-methyl-1,2-heneicosanediol	C25H42O	FA	DW	NP
2.08_393.2754	2.08	393.2754	[M+K]+	354.3134	2	4 oxodocosanoic acid derivatives	C22H42O3	FA	DW	NP
3.75_494.3259	3.75	494.3259	[M+CH3OH+H]+	461.2930	1	AMC arachidonoyl amide	C30H39NO3	FA	DW	NP
3.59_518.3227	3.59	518.3227	[M+2Na-H]+	473.3505	1	Docosa-4,7,10,13,16-pentaenoyl carnitine, clupanodonyl carnitine	C29H47NO4	FA	DW	NP
2.23_361.2844	2.23	361.2844	[M+2H]2+	720.5543	0	7 PC derivatives	C39H79NO8P	GP	DW	PL
3.59_518.3227	3.59	518.3227	[M+Na]+	495.3325	1	5 PC derivatives	C24H50NO7P	GP	DW	PL
2.46_363.3007	2.46	363.3007	NC					NC	DW	
1.66_533.1525	1.66	533.1525	[M+2Na-H]+	488.1835	4	Sericetin diacetate	C29H28O7	PK	DW	PL
3.94_1039.664	3.94	1039.6640	[M+2Na−H]+	994.6944	1	Dodecaprenyl diphosphate	C60H100O7P2	PR	DW	PL
5.21_337.2678	5.21	337.2678	[M+H−2H2O]+	372.2777	8	Steroid derivative (structurally similar to Finasteride)	C23H36N2O2	ST	DW	NP
2.20_377.2804	2.20	377.2804	[M+CH3OH+H]+	344.2464	1	6 steroid derivatives (structurally similar to hydroxystanozolol or epistanolozolol)	C21H32N2O2	ST	DW	NP
2.30_379.2931	2.30	379.2931	[M+Li]+	372.2777	1	Steroid derivative (structurally similar to Finasteride)	C23H36N2O2	ST	DW	NP
2.24_379.294	2.24	379.2940	[M+Li]+	372.2777	0	Steroid derivative (structurally similar to Finasteride)	C23H36N2O2	ST	DW	NP
2.22_395.2897	2.22	395.2897	[M+Li]+	388.2726	2	Steroid derivative (structurally similar to o-Hydroxyfinasteride)	C23H36N2O3	ST	DW	NP
6.96_413.3784	6.96	413.3784	[M+H−H2O]+	430.3811	0	8 Vitamin D3 derivatives	C29H50O2	ST	DW	NP
2.21_417.2756	2.21	417.2756	[M+Na]+	394.2872	1	Vitamin D3 derivative	C27H38O2	ST	DW	NP
6.28_427.3589	6.28	427.3589	[M+H−2H2O]+	462.3709	1	Vitamin D3 derivative	C29H50O4	ST	DW	NP

The METLIN: Metabolite and Tandem MS Database was used to assign tentative compound identities and empirical formulas to the 33 influential ions with maximal m/z error set at 10 ppm under positive ionization adduct scan modes. Tentative identities for each ion were chosen based on smallest accurate mass error (AME) compared to the queried ion m/z; in the event of an AME tie, adducts with same AME but different empirical formulas are reported. Table columns: Ion Identifier = identifier used in **Figure 4** **, Panel 4B**; Ion RT = ion retention time in minutes; Ion m/z = ion mass-to-charge ratio in daltons; Adduct = positive ionization adduct; Adduct Mass = mass of ion + adduct; Dppm = change (Δ, or d) in ppm from Ion m/z, used to determine AME; Tentative Identity = identifier with smallest AME from potential hits; Empirical Formula = derived from Adduct Mass and Dppm; Class = class assignation according to the Lipid Maps Lipidomics Gateway as: 1) fatty acyls (FA), 2) glycerolipids (GL), 3) glycerophospholipids (GP), 4) sphingolipids (SP), 5) sterol lipids (ST), 6) prenol lipids (PR), 7) saccharolipids (SL), 8) polyketides (PK), or NC if tentative identities could not be matched to the ion m/z; Overexpressed in: = ion spectral intensity higher in bread wheat (BW) or durum wheat (DW) as indicated.

For the comparison of tetraploid DW to hexaploid BW lines, 31 of 35 ions were assigned tentative identities and classified according to the Lipid Classification System employed by the Lipid Maps Lipidomics Gateway which utilizes 8 categories: 1) fatty acyls (FA), 2) glycerolipids (GL), 3) glycerophospholipids (GP), 4) sphingolipids (SP), 5) sterol lipids (ST), 6) prenol lipids (PR), 7) saccharolipids (SL), 8) polyketides (PK) [26], [27], or a final class, NC, applied to the 4 ions for which no tentative identity or class could be assigned. Assignment of each ion to the appropriate category was achieved by consulting the Lipidomics Gateway and related publications [26]–[28] and is reported in Table 2. Of the 35 influential ions, 16 ions were overexpressed in BW compared to DW; as depicted in Figure 4C and described in detail in Table 2, 10 (62.5%) of these elevated ions were tentatively identified as polar GL, including derivatives of monoacylglycerol (MG) (retention time (RT): 6.58, 7.43; m/z: 377.3415, 339.2898), diacylglycerol (DG) (RT: 2.34, 7.07, 7.41; m/z: 353.2280, 617.5147, 595.5318), and triacylglycerol (TG) (RT: 6.14, 7.42; m/z: 349.3104, 937.6763); and derivatives of phosphatidic acid (PA) (RT: 8.50, 8.50; m/z: 719.4624, 741.4486), and phosphatidylcholine/phosphatidyl-ethanolamine (RT: 7.07; m/z: 782.5360).

Conversely, 19 ions were overexpressed in DW compared to BW; as described in detail in Table 2, 14 (74%) of these were tentatively identified as nonpolar ST and FA, including vitamin D3 derivatives (RT: 2.21, 6.28, 6.96; m/z: 417.2756, 427.3589, 413.3784) and very long chain fatty acid derivatives (RT: 2.08, 2.38, 2.46, 3.75; m/z: 393.2754, 381.3093, 381.3096, 494.3259). The elevated content of putative polar lipids in BW, compared to the elevated content of putative nonpolar lipids in DW, suggests that usage of acetyl coenzyme A (CoA), the common synthetic precursor molecule in synthesis of each class of lipids, may be differentially regulated in tetraploid durum compared to hexaploid BW classes.

To highlight metabolite profiles characteristic of classes within the hexaploid wheat species, a two-class OPLS-DA model comparing hexaploid HBW to SBW lines was created. Overall model performance is depicted in Figure S2 and model fit is reported in Table S1. Based on the excellent model fit observed, this model was used to generate the S-plot shown in Figure S3A, where 31 ions (enlarged icons) were selected as features with high discriminatory capacity. This model performed well, with R2Y(cum) = 95.0% and QY(cum) = 64.2%. Figure S3B shows the 95% confidence intervals for covariance of the 31 discriminatory ions in the first principal component sorted in ascending order, indicating that all 31 ions were statistically significant ions for separation of HBW from SBW. Tentative identities for these ions were obtained using METLIN, and “best-choice” tentative identities, retention time, adduct, and tentative empirical formula are reported in Table S3, with all stereoisomers and tentative adduct identities provided in Table S4.

For the comparison of HBW to SBW lines, 26 of 31 ions were tentatively identified and classified according to the Lipid Classification System employed by the Lipid Maps Lipidomics Gateway classification system; due to the polar nature of the discriminatory ions in this comparison, ions not readily classified into the Lipid Maps schematic were classified into 2 additional chemical classes: OS, for organosulfur compounds; ON, for organonitrogen compounds; or NC for the 5 ions for which tentative identity or class could not be assigned. Of the 12 ions identified as overexpressed in HBW, 10 of which were assigned tentative identities, 3 ions (25%) were tentatively identified as glycerophospholipids (GP), 3 ions (25%) were tentatively identified as glycerolipids (GL), and 3 ions were tentatively identified as organonitrogen compounds (ON) of heterocyclic amine ontology. Conversely, of the 19 ions overexpressed in SBW, 16 of which were assigned tentative identities, 6 ions (32%) were tentatively identified as polyketides (PK- primarily flavonoids) and 6 ions (32%) were tentatively identified as glycerophospholipids (GP).

## Discussion

Figures 1–4 provide evidence that the genetic individuality of wheat classes and subclasses permitted chemical separation of commonly grown wheat lines (described in Table 1) without controlling for environmental effects. Thus, this study constitutes a proof-in-principle of the ability of metabolite profiling to drive hypothesis generation through identification of plant metabolites and potential pathways of metabolite biosynthesis that distinguish among wheat classes. These findings support the use of global high-throughput metabolite profiling as a discovery tool capable of identifying a specific pattern of ion expression, or ‘profile’, responsible for traits of interest. In turn, identified profiles can be used for dedicated analytical procedures and as a routine, cost-effective screening tool that can rapidly evaluate large numbers of plant varieties for profiles associated with desirable or undesirable traits. These implications are discussed in greater detail below.

While two ploidy levels, tetraploid and hexaploid, distinguish between the major types of wheat consumed by humans, to our knowledge the application of broad-scale, metabolite profiling has not been utilized to determine whether these species have distinguishing chemical profiles, which may have agronomic and biomedical implications. As shown in Figure 1, metabolite profiling distinguished between DW and BW with 100% accuracy, indicating that any given DW line is more chemically similar to other DW lines than to any BW line. In addition to highlighting the distinct chemical characteristics of DW versus BW, Figure 1 provides information regarding both chemical diversity and similarity. The dendrogram depicted in Figure 1C indicates that DW lines have a highly distinct chemical footprint, as cluster 1 (comprising DW) has low compactness and high distinctness compared to cluster 2 (comprising BW lines) based on node height.

Dendrograms provide estimates of chemical similarity based on the hierarchical distance at which wheat lines cluster. The following example illustrates the value of this information. In Figure 1C, cluster 2A, HBW lines 1 and 20 were the first to cluster based on vertical distance from 0, and thus are the most chemically similar lines within the dataset. If, for example, line 20 was a well-established cultivar with a desirable chemical trait such as overexpression of a bioactive molecule, the hierarchical distance suggests that line 1 is chemically similar to line 20 and has higher likelihood of exhibiting the same chemical traits than a wheat line at a greater hierarchical distance. This capacity may be of particular use to plant breeders when choosing elite parents for developing breeding programs for enhancement of beneficial traits through heterosis, or hybrid vigor.

Although this is the first report on the chemical uniqueness of tetraploid DW and hexaploid BW, this finding is not unexpected given that DW and BW differ by an additional set of chromosomes–it stands to reason that the addition of an entire genome would substantially alter the metabolite profile. Indeed, enzyme multiplicity, due to coding of the same enzyme by multiple chromosomes, is postulated by Feldman and Levy in 2005 [29] to be at least partially responsible for the environmental adaptability of BW, which is conferred largely by the metabolome. Furthermore, the 2-class model, comparing DW to all BW lines, had the best model fit of all comparisons with R2Y(cum) = 95.4%, Q = 82.0%, and 0 lines misclassified, indicating that tetraploid DW lines are, chemically speaking, very different than hexaploid BW lines.

However, the capacity of metabolite profiling to distinguish between classes was not restricted to large-scale genetic differences. As shown in the 3-class model in Figure 1B and the 2-class model in Figure S2, metabolite profiling was also able to distinguish between HBW and SBW with 100% accuracy for the lines evaluated, suggesting that considerable variation in metabolite expression is conferred by genetic differences between wheat classes of the same species and ploidy level.

In addition to distinguishing between wheat lines of different ploidy levels, metabolite profiling also distinguished between subclasses of the major wheat classes based on minor genetic differences related to growing season (winter vs. spring) and seed coat color (white vs. red). In the HBW model shown in Figure 2, the metabolite profile distinguished between 27 wheat lines comprising 4 subclasses with ∼63% accuracy; however, the low R2(cum) indicates a great deal of chemical homogeneity among HBW lines, making distinguishing between subclasses difficult. Node height of cluster 2B in Figure 1C confirms the high degree of compactness in HBW lines.

As seen in Figure 3, the SBW model distinguished between 12 wheat lines comprising 3 subclasses with 100% accuracy, suggesting that these subclasses have chemical profiles with high discriminatory capacity. Interestingly, the high proportion of within-class variation (R2X_O1–3_ = 40.0%) indicates that other factors contribute strongly to chemical profiles of SBW; further investigation and larger sample sizes are needed to understand this phenomenon. However, despite small sample size, metabolite profiling was capable of distinguishing between wheat subclasses, which is consistent with previous studies by Heuberger *et al.* in rice [30], where chemical diversity within varieties of the rice species, *Oryza sativa,* clustered according to their defined species subclassifications: *indica*, *japonica*, and *aus*.

In addition to characterization of wheat class chemical profiles, OPLS-DA also provided information regarding discriminatory ions responsible for the distinction between tetraploid and hexaploid wheat classes. Thirty-six ions were identified using the S-plot of Figure 4A and jack-knifed confidence interval of modeled correlation between X-variables (ions) and Y variables (classes) identified 35 of these ions as statistically significant for influencing the separation of DW and BW, which were then assigned tentative identities and empirical formulas using METLIN: Metabolite and Tandem MS Database. Information for each ion, including retention time, m/z of the ion, tentative identity, empirical formula, lipid class, polarity class, and wheat class in which the ion is overexpressed (BW versus DW) are reported in Table 2. A total of 16 ions were overexpressed in BW, of which 10 (62.5%) were tentatively identified as polar lipids; in contrast 19 ions were overexpressed in DW, of which 14 (74%) were tentatively identified as nonpolar lipids according to the Lipid Maps Lipidomics Gateway classification system as described by Fahy *et al.* [26]–[28] and as polar versus nonpolar according to Chung *et al.* in 2009 [31].

These distinct patterns of nonpolar versus polar lipid expression indicate that lipid class profiles are responsible for the separation of clusters 1 and 2 in Figure 1C, which illustrate the discrete chemical profile of tetraploid DW compared to hexaploid BW. To illustrate the capacity of metabolite profiling for hypothesis generation, the prevalence of class-discriminating phospholipids, including glycerolipids and glycerophospholipids, in BW at the apparent expense of nonpolar lipids may suggest that the DD genome confers a preferential shunting of cellular carbon into the fatty acyl synthetic pathway in the plant plastid, in which acetyl CoA molecules are cyclically condensed with malonyl CoA for carbon chain elongation at the expense of the mevalonate pathway, which provides isoprenoid precursor molecules for NP steroid biosynthesis [28], [32]. The fatty acyl synthetic pathway is the starting point for synthesis of polar glycerolipid and glycerophospholipids, in which fatty acids are transported from the plastid to the endoplasmic reticulum for conjugation to a glycerol backbone by acyl Coa:*sn-*glycerol-3-phosphate acyltransferase, the rate limiting enzyme for formation of both glycerolipid and glycerophospholipid [33]. Thus, while future studies are required to validate this hypothesis, metabolite profiling serves as a way to establish potential links between plant chemicals and observed biological phenomena.

To further highlight the utility of metabolite profiling, discriminatory ions in the separation of HBW and SBW are reported in Tables S3 and S4. In contrast to the differential polarity of metabolites that separate DW from BW, nearly all discriminatory ions in the separation of BW classes were tentatively identified as polar compounds. However, the biosynthetic origin of the major class of discriminatory polar compounds varies; of the 12 ions identified as overexpressed in HBW, 10 of which were assigned tentative identities, 3 ions (25%) were tentatively identified as glycerophospholipids, 3 ions (25%) were tentatively identified as glycerolipids, and 3 ions were tentatively identified as organonitrogen compounds of heterocyclic amine ontology. Conversely, of the 19 ions overexpressed in SBW, 16 of which were assigned tentative identities, 6 ions (32%) were tentatively identified as polyketides [34] (primarily flavonoids), suggesting that the utilization of glycolytic intermediates, e.g. phosphoenolpyruvate, into phenolic or alkaloid biosynthesis via the shikimate pathway, as reviewed in [34] vs. synthesis of acetyl CoA for glycerolipid/glycerophospholipid biosynthesis may be differentially regulated between HBW and SBW.

Though the exploratory nature of metabolite identification through profiling techniques must be emphasized, the differential expression of polar and nonpolar lipids in major wheat classes is supported by Armanino *et al.* [35], whose work demonstrated that lipid profiling was a way to reliably distinguish between DW and BW, and by Chung *et al.*, whose summary of several published articles indicated that the ratio of polar to nonpolar lipids is generally higher in BW vs. DW [31]. Additionally, though relative lipid abundance in wheat seed is minor (3.5% seed mass), lipid polarity impacts the location of lipid synthesis and aggregation within the plant cell [36], [37], which has implications for bread dough properties such as viscosity, pasting, and foaming [31], and in loaf quality properties such as gas bubble formation, leavening capacity, and final loaf volume (reviewed in [31]), making the distinct chemical profiles described herein of value to breeders concerned with traits of bread quality. Thus, the differential expression of lipid classes highlights the utility of metabolite profiling for hypothesis generation. Finally, the agreement of our experimental findings, in which environment and growing year were not controlled, with the general conclusions obtained by the work of Armanino *et al.* [35], that polar lipids distinguish between ploidy levels, and which employed very stringent environmental control, provides evidence that metabolite profiling can ultimately serve as a quick, relatively inexpensive method of determining which compounds are heavily influential in the chemical distinction between genotypes.

## Limitations

Plants synthesize small molecules to aid in reproduction, assist communication within and among plant species [38], and as a means of dealing with biotic (other plants, pests, animals) and abiotic (temperature, drought, soil quality) stressors [39], [40]. Indeed, secondary metabolites under the Poaceae family, to which all wheat species belong, have been reported to vary in concentration based on abiotic, environmental stressors including water availability, light intensity, and temperature [41], [42]. Environmental effects on gene expression are likely to account, at least in part, for the orthogonal sources of variation observed in the OPLS -DA modeling. This is consistent with the ability of metabolite profiling not only to provide valuable information about traits based on genetic differences, but potentially to elucidate how gene-by-environment interactions affect chemical profiles associated with traits of interest. Nonetheless, as environment was not controlled in seed selection for these analyses, it is not possible to determine whether the variation due to environment was accounted for in the systematic variation in metabolite concentration orthogonal to that associated with wheat class, or in the variation currently identified as noise. The small sample size, particularly in the subclass models, is reflected in the low predictive reliability (Q2Y(cum)) of the models, which ranged from 82.1% in the DW vs. BW model to 17.3% in the HBW subclasses model. These will be important factors to consider in the design of future experiments to investigate the effect of gene-by-environment interactions on traits of interest.

### Concluding Comments

This is the first comprehensive study of the systemic metabolic state of wheat, utilizing high-throughput, semi-quantitative chromatographic (UPLC-TOF-MS) techniques, in order to characterize the impact of genetic differences between wheat classes on metabolite expression profiles with the goal of determining which ions, and classes of ions, distinguish between major wheat classes. In accomplishing this objective, this work sets the stage for the second objective of metabolite profiling as described by Kopka *et al.*, i.e. to characterize the regulatory mechanisms responsible for the discriminatory metabolic states that were identified [12]. This report also provides a foundation for future applications of metabolite profiling for the improvement of wheat for enhanced agronomic and human health traits.

## Supporting Information

Figure S1 **Metabolite profiling workflow for analysis of 3 major classes of wheat.** UPLC-TOF-MS = ultraperformance liquid chromatography with time-of-flight mass spectrometer; C18 = carbon chain length on stationary phase; PCA = principal components analysis; OPLS-DA = orthogonal projections to latent structures discriminant analysis; DW = durum wheat; BW = bread wheat, including hard (HBW) and soft (SBW) bread wheat classes; JKCI = jack-knifed 95% confidence interval of modeled covariance in the first predictive principal component; ppm = parts per million of accurate mass error.
(TIF)

Figure S2 **Metabolite profiling distinguishes between hexaploid hard and soft bread wheat classes with 100% accuracy.** Multivariate discriminant analysis of the high-quality ion list, consisting of 935 ions in 39 wheat lines, was used to distinguish between hexaploid hard (HBW) and soft (SBW) bread wheat classes. Each point represents a single observation (e.g. each wheat line). **(Panel 1A)** To visualize inherent clustering patterns, the scatter plot represents unsupervised analysis through the PCA model comparing HBW to SBW lines. Separation of HBW and SBW lines is observed. Model fit: R2X(cum) = 64.8%, with 7 components, and Q2(cum) = 27.6%. **(Panel 1B)** To determine contributing sources of variation, the scatter plot represents supervised analysis of the 2-class OPLS-DA model, which rotates the model plane to maximize separation due to class assignment. Complete separation of HBW and SBW was observed. Model fit: R2Y(cum) = 95.0%, Q2Y(cum) = 64.2%. **(Panel 1C-Inset)** The misclassification table for the 3-class OPLS-DA model indicates that 100% of wheat lines were correctly classified, with low probability (p = 2.60E−10) of random table generation as assessed by Fisher’s Exact Probability. **(Panel 1C)** To visualize the misclassification rate, the dendrogram depicts hierarchical clustering patterns among major wheat classes using single linkage and size. Two main clusters completely separate 1) HBW lines and 2) SBW lines, indicating chemical distinctness between these classes.
(TIF)

Figure S3 **Discriminatory ions determine separation of hexaploid HBW from SBW lines.** Multivariate analysis was extended to identify influential ions responsible for the separation between classes. **(Panel 4A)** The supervised OPLS-DA model was created to compare all HBW lines to all SBW lines, and an S-plot was constructed by plotting modeled correlation against modeled covariation from the first predictive component. Upper right and lower left regions of S-plots contain candidate biomarkers with both high reliability and high magnitude; discriminatory ions (n = 31) chosen from these regions are enlarged X3. **(Panel 4B)** To determine the statistical reliability of the ions chosen in **Panel 4A**, jack-knifed confidence intervals (JKCI) were created on the magnitude of covariance in the first component for the 31 ions and sorted in ascending order based on expression in durum wheat; all ions were statistically significant at this level, resulting in n = 31 ions responsible for the separation of HBW from SBW lines. Tentative identities for these discriminatory ions are provided in **Table S3.**
(TIF)

Table S1 **Model fit summaries of unsupervised and supervised analyses.** Table columns: Model = wheat lines used to construct OPLS-DA model; R2X_p_ = variation in X variables (ions) explained by predictive principal components; R2X_o_ = variation in X variables explained by orthogonal principal components; R2X(cum) = total amount of explained variation in X (R2X_p_ + R2X_o_); R2Y(cum) = total amount of variation explained in Y; Q2Y(cum) = total amount of predicted variability in Y, estimated by 7-fold cross validation; Wheat Lines Misclassified = number of wheat lines misclassified by the model.
(DOCX)

Table S2 **All tentative identities for discriminatory ions in the DW vs. BW OPLS-DA model.** The METLIN: Metabolite and Tandem MS Database was used to assign tentative compound identities and empirical formulas to the 35 influential ions with maximal m/z error set at 10 ppm under positive ionization adduct scan modes. Table columns: Ion Identifier; Ion RT = ion retention time in minutes; Ion m/z = ion mass-to-charge ratio in daltons; Adduct = positive ionization adduct; Adduct Mass = mass of ion + adduct; Dppm = change (Δ, or d) in ppm from Ion m/z; Tentative Identity = identifier from METLIN; Empirical Formula = derived from Adduct Mass and Dppm. NC = not classified; no tentative compound identity hits within limits of METLIN search.
(DOCX)

Table S3 **Best-choice tentative identities of discriminatory ions in the hexaploid HBW vs. SBW OPLS-DA model.** The METLIN: Metabolite and Tandem MS Database was used to assign tentative compound identities and empirical formulas to the 31 influential ions with maximal m/z error set at 10 ppm under positive ionization adduct scan modes. Table columns: Ion Identifier (used in Figure S3, Panel A); Ion RT = ion retention time in minutes; Ion m/z = ion mass-to-charge ratio in daltons; Adduct = positive ionization adduct; Adduct Mass = mass of ion + adduct; Dppm = change (Δ, or d) in ppm from Ion m/z; Tentative Identity = identifier from METLIN; Empirical Formula = derived from Adduct Mass and Dppm; Class = class assignation according to the Lipid Maps Lipidomics Gateway as: 1) fatty acyls (FA), 2) glycerolipids (GL), 3) glycerophospholipids (GP), 4) sphingolipids (SP), 5) sterol lipids (ST), 6) prenol lipids (PR), 7) saccharolipids (SL), 8) polyketides (PK), additional classes ON (organonitrogen) and OS (organosulfur compounds), with NC classification if tentative identities could not be matched to the ion m/z; Overexpressed in: = ion spectral intensity higher in hard bread wheat (HBW) or soft bread wheat (SBW) as indicated.
(DOCX)

Table S4 **Characteristics and all possible tentative identities for discriminatory ions in the hexaploid HBW vs. SBW OPLS-DA model.** The METLIN: Metabolite and Tandem MS Database was used to assign tentative compound identities and empirical formulas to the 31 influential ions with maximal m/z error set at 10 ppm under positive ionization adduct scan modes. Table columns: Ion Identifier; Ion RT = ion retention time in minutes; Ion m/z = ion mass-to-charge ratio in daltons; Adduct = positive ionization adduct; Adduct Mass = mass of ion + adduct; Dppm = change (Δ, or d) in ppm from Ion m/z; Tentative Identity = identifier from METLIN; Empirical Formula = derived from Adduct Mass and Dppm. NC = not classified; no tentative compound identity hits within limits of METLIN search.
(DOCX)
