# Supplementary figures and images for: Metabolite Profiling of a Diverse Collection of Wheat Lines Using Ultraperformance Liquid Chromatography Coupled with Time-of-Flight Mass Spectrometry
Source: PLoS One. 2012 Aug 30;7(8):e44179. doi: 10.1371/journal.pone.0044179 (PMC3431305; doi:10.1371/journal.pone.0044179)

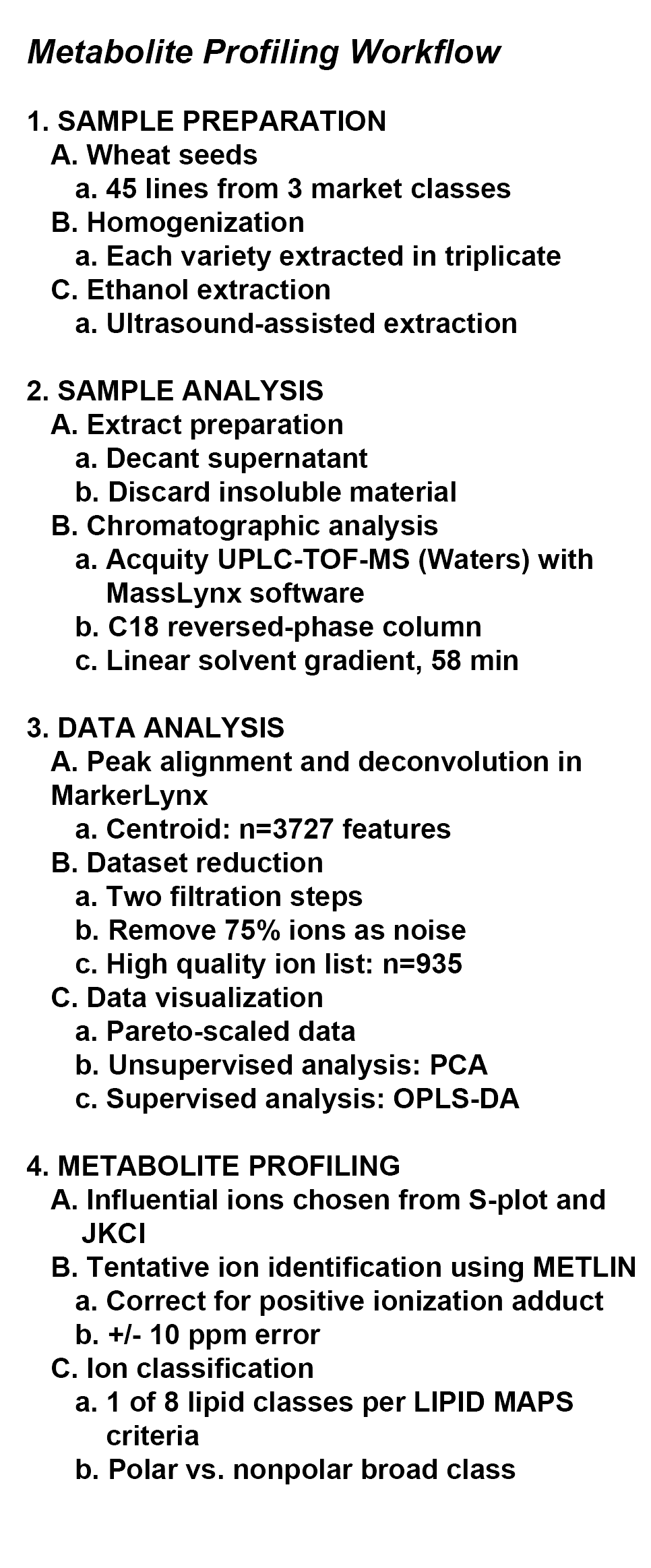

Supplement: Figure S1 — Metabolite profiling workflow for analysis of 3 major classes of wheat. UPLC-TOF-MS = ultraperformance liquid chromatography with time-of-flight mass spectrometer; C18 = carbon chain length on stationary phase; PCA = principal components analysis; OPLS-DA = orthogonal projections to latent structures discriminant analysis; DW = durum wheat; BW = bread wheat, including hard (HBW) and soft (SBW) bread wheat classes; JKCI = jack-knifed 95% confidence interval of modeled covariance in the first predictive principal component; ppm = parts per million of accurate mass error. (TIF) [file pone.0044179.s001.tif]

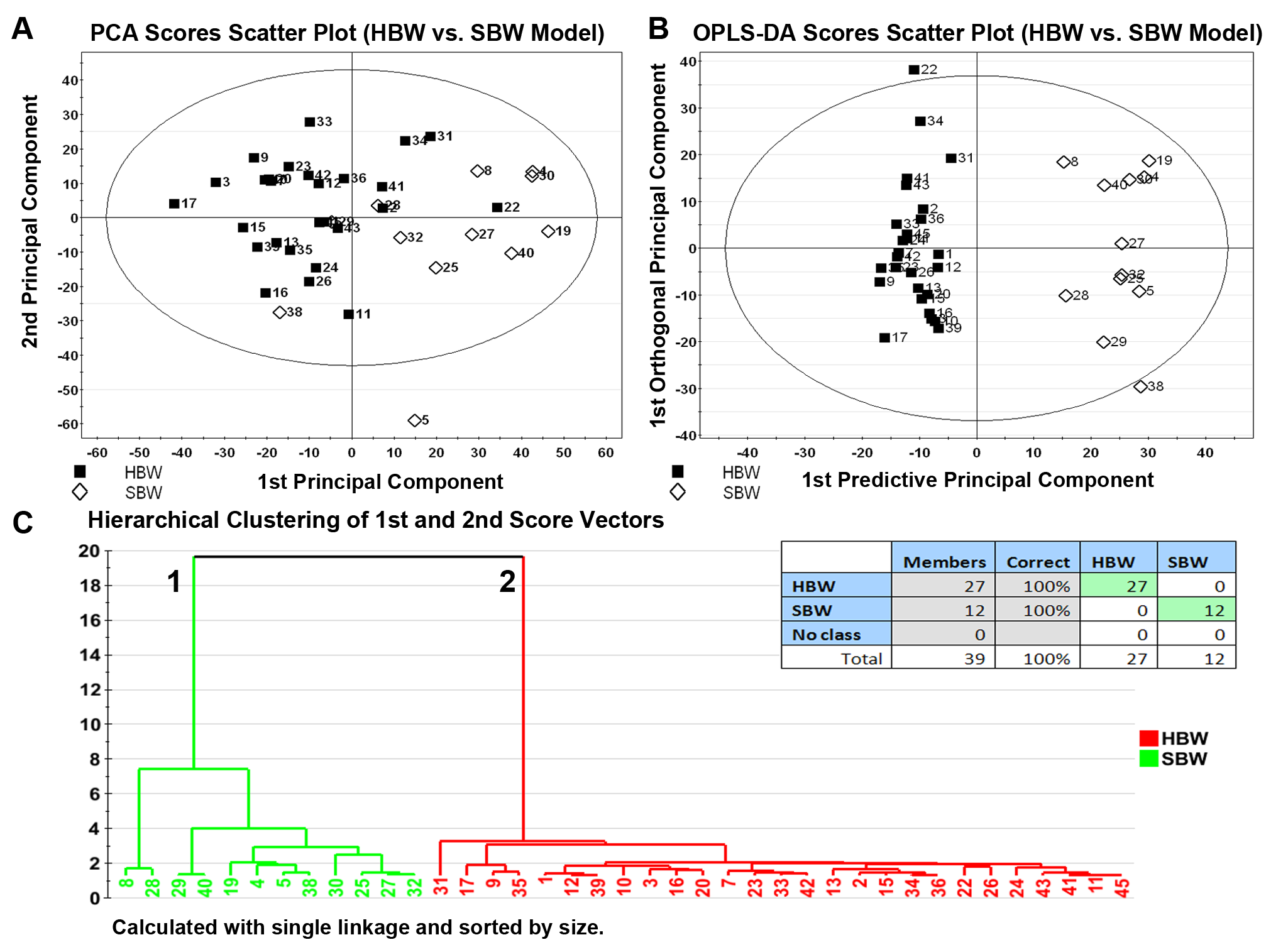

Supplement: Figure S2 — Metabolite profiling distinguishes between hexaploid hard and soft bread wheat classes with 100% accuracy. Multivariate discriminant analysis of the high-quality ion list, consisting of 935 ions in 39 wheat lines, was used to distinguish between hexaploid hard (HBW) and soft (SBW) bread wheat classes. Each point represents a single observation (e.g. each wheat line). (Panel 1A) To visualize inherent clustering patterns, the scatter plot represents unsupervised analysis through the PCA model comparing HBW to SBW lines. Separation of HBW and SBW lines is observed. Model fit: R2X(cum) = 64.8%, with 7 components, and Q2(cum) = 27.6%. (Panel 1B) To determine contributing sources of variation, the scatter plot represents supervised analysis of the 2-class OPLS-DA model, which rotates the model plane to maximize separation due to class assignment. Complete separation of HBW and SBW was observed. Model fit: R2Y(cum) = 95.0%, Q2Y(cum) = 64.2%. (Panel 1C-Inset) The misclassification table for the 3-class OPLS-DA model indicates that 100% of wheat lines were correctly classified, with low probability (p = 2.60E−10) of random table generation as assessed by Fisher’s Exact Probability. (Panel 1C) To visualize the misclassification rate, the dendrogram depicts hierarchical clustering patterns among major wheat classes using single linkage and size. Two main clusters completely separate 1) HBW lines and 2) SBW lines, indicating chemical distinctness between these classes. (TIF) [file pone.0044179.s002.tif]

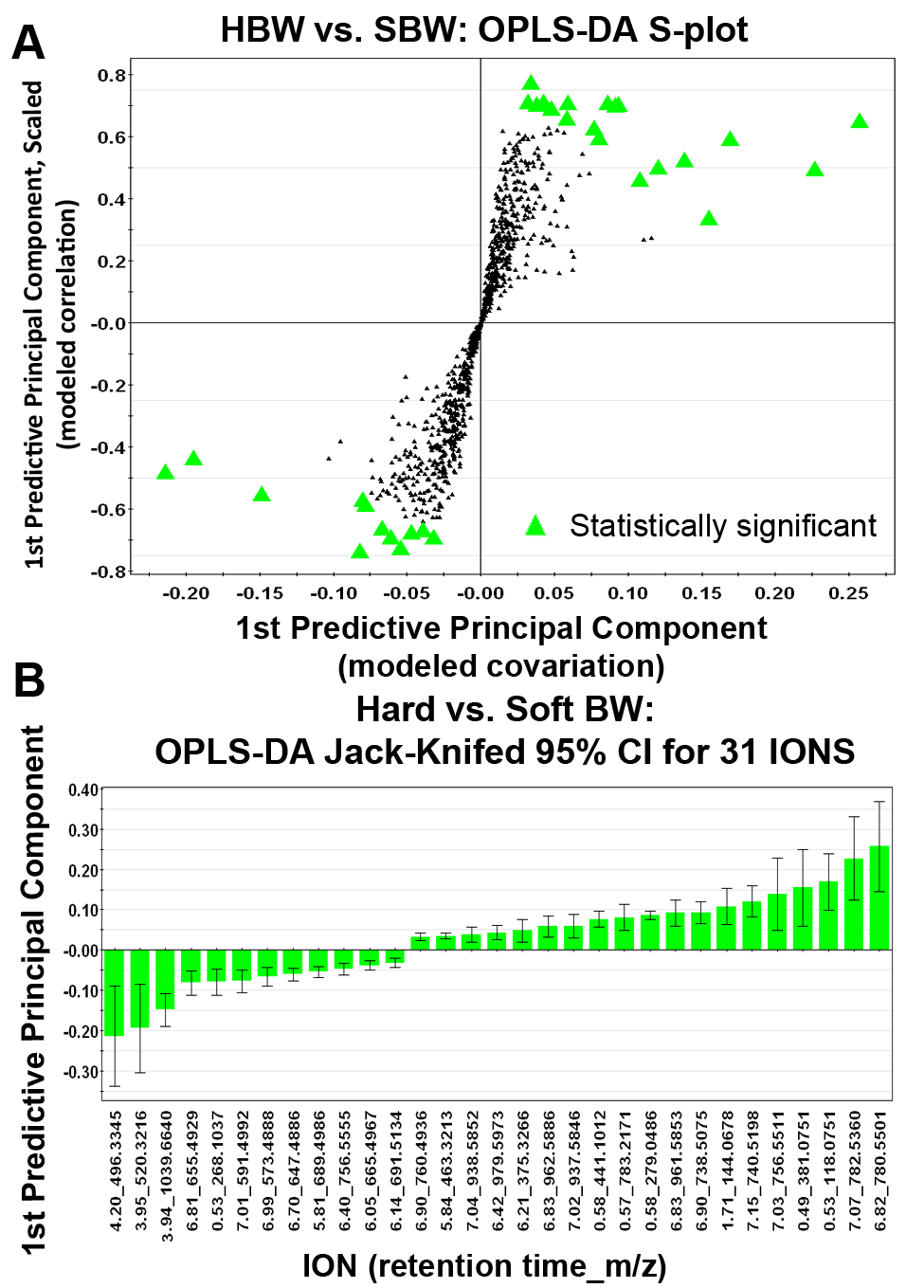

Supplement: Figure S3 — Discriminatory ions determine separation of hexaploid HBW from SBW lines. Multivariate analysis was extended to identify influential ions responsible for the separation between classes. (Panel 4A) The supervised OPLS-DA model was created to compare all HBW lines to all SBW lines, and an S-plot was constructed by plotting modeled correlation against modeled covariation from the first predictive component. Upper right and lower left regions of S-plots contain candidate biomarkers with both high reliability and high magnitude; discriminatory ions (n = 31) chosen from these regions are enlarged X3. (Panel 4B) To determine the statistical reliability of the ions chosen in Panel 4A, jack-knifed confidence intervals (JKCI) were created on the magnitude of covariance in the first component for the 31 ions and sorted in ascending order based on expression in durum wheat; all ions were statistically significant at this level, resulting in n = 31 ions responsible for the separation of HBW from SBW lines. Tentative identities for these discriminatory ions are provided in Table S3. (TIF) [file pone.0044179.s003.tif]
