# Supplementary material for: Metabolite Profiling of a Diverse Collection of Wheat Lines Using Ultraperformance Liquid Chromatography Coupled with Time-of-Flight Mass Spectrometry
Source: PLoS One. 2012 Aug 30;7(8):e44179. doi: 10.1371/journal.pone.0044179 (PMC3431305; doi:10.1371/journal.pone.0044179)
Supplement: Table S1 — Model fit summaries of unsupervised and supervised analyses. Table columns: Model = wheat lines used to construct OPLS-DA model; R2Xp = variation in X variables (ions) explained by predictive principal components; R2Xo = variation in X variables explained by orthogonal principal components; R2X(cum) = total amount of explained variation in X (R2Xp + R2Xo); R2Y(cum) = total amount of variation explained in Y; Q2Y(cum) = total amount of predicted variability in Y, estimated by 7-fold cross validation; Wheat Lines Misclassified = number of wheat lines misclassified by the model. (DOCX) [file pone.0044179.s004.docx]

| **PCA Model** | **R2X(cum) (# of components)** | | **Q2(cum)** | |  |  |  |
| --- | --- | --- | --- | --- | --- | --- | --- |
| Figure 1A: Durum vs. HBW vs. SBW (3-Class) | 68.6% (7) | | 38.9% | |  |  |  |
| Figure 2A: HBW Subclasses (4-Class) | 40.3% (3) | | 10.8% | |  |  |  |
| Figure 3A: SBW Subclasses (3-Class) | 48.9% (2) | | 4.9% | |  |  |  |
| Figure 4: (scatter plot not shown) DW vs. All BW (2-Class) | 68.6% (7) | | 38.9% | |  |  |  |
| Supplemental Figure 2A: HBW vs. SBW (2-Class) | 64.8% (7) | | 27.6% | |  |  |  |
|  | **X** | | | | **Y** | |  |
| **OPLS-DA Model** | **R2X_p_ (number of components)** | **R2X_o_ (number of components)** | | **R2X(cum)** | **R2Y(cum)** | **Q2Y(cum)** | **Wheat Lines Misclassified** |
| Figure 1B: Durum vs. HBW vs. SBW (3-Class) | 22.0% (2) | 37.7% (4) | | 59.7% | 93.2% | 71.0% | 0/45 |
| Figure 2B: HBW Subclasses (4-Class) | 9.7% (1) | 11.4% (1) | | 21.1% | 36.6% | 17.3% | 10/27 |
| Figure 3B: SBW Subclasses (3-Class) | 32.1% (2) | 40.0% (3) | | 72.1% | 99.1% | 64.9% | 0/12 |
| Figure 4 (scatter plot not shown): DW vs. All BW (2-Class) | 12.6% (1) | 38.9% (3) | | 51.5% | 95.4% | 82.1% | 0/45 |
| Supplemental Figure 2A: HBW vs. SBW (2-Class) | 12.8% (1) | 30.7% (3) | | 43.5% | 95.0% | 64.2% | 0/39 |

**Table S1. Model fit summaries of unsupervised and supervised analyses.**
