# Supplementary material for: Metabolite Profiling of a Diverse Collection of Wheat Lines Using Ultraperformance Liquid Chromatography Coupled with Time-of-Flight Mass Spectrometry
Source: PLoS One. 2012 Aug 30;7(8):e44179. doi: 10.1371/journal.pone.0044179 (PMC3431305; doi:10.1371/journal.pone.0044179)
Supplement: Table S2 — All tentative identities for discriminatory ions in the DW vs. BW OPLS-DA model. The METLIN: Metabolite and Tandem MS Database was used to assign tentative compound identities and empirical formulas to the 35 influential ions with maximal m/z error set at 10 ppm under positive ionization adduct scan modes. Table columns: Ion Identifier; Ion RT = ion retention time in minutes; Ion m/z = ion mass-to-charge ratio in daltons; Adduct = positive ionization adduct; Adduct Mass = mass of ion + adduct; Dppm = change (Δ, or d) in ppm from Ion m/z; Tentative Identity = identifier from METLIN; Empirical Formula = derived from Adduct Mass and Dppm. NC = not classified; no tentative compound identity hits within limits of METLIN search. (DOCX) [file pone.0044179.s005.docx]

**Table S2. All tentative identities for discriminatory ions in the DW vs. BW OPLS-DA model.**

| **Ion Identifier** | **Ion RT** | **Ion m/z** | **Adduct** | **Adduct Mass** | **Dppm** | **Tentative Identity** | **Empirical Formula** | **Overexpressed In:** |
| --- | --- | --- | --- | --- | --- | --- | --- | --- |
| 0.49_104.1077 | 0.49 | 104.1077 | [M+H+Na]2+ | 184.2191 | 0 | Tridecane | C13H28 | BW |
| 0.49_104.1077 | 0.49 | 104.1077 | [M+H]+ | 103.0997 | 6 | Choline | C5H13NO | BW |
| 0.49_104.1077 | 0.49 | 104.1077 | [M+H]+ | 103.0997 | 6 | 2-Amino-3-methyl-1-butanol | C5H13NO | BW |
| 1.67_325.1129 | 1.67 | 325.1129 | [M+H+Na]2+ | 626.2296 | 0 | Tetrahydropteroyltri-L-glutamate | C24H34N8O12 | DW |
| 1.67_325.1129 | 1.67 | 325.1129 | [M+H-H2O]+ | 342.1162 | 1 | Gentiobiose | C12H22O11 | DW |
| 1.67_325.1129 | 1.67 | 325.1129 | [M+H-H2O]+ | 342.1162 | 1 | Leucrose | C12H22O11 | DW |
| 1.67_325.1129 | 1.67 | 325.1129 | [M+H-H2O]+ | 342.1162 | 1 | 3-b-Galactopyranosyl glucose | C12H22O11 | DW |
| 1.67_325.1129 | 1.67 | 325.1129 | [M+H-H2O]+ | 342.1162 | 1 | D-(+)-Turanose | C12H22O11 | DW |
| 1.67_325.1129 | 1.67 | 325.1129 | [M+H-H2O]+ | 342.1162 | 1 | Kojibiose | C12H22O11 | DW |
| 1.67_325.1129 | 1.67 | 325.1129 | [M+H-H2O]+ | 342.1162 | 1 | Sucrose | C12H22O11 | DW |
| 1.67_325.1129 | 1.67 | 325.1129 | [M+H-H2O]+ | 342.1162 | 1 | Lactose | C12H22O11 | DW |
| 1.67_325.1129 | 1.67 | 325.1129 | [M+H-H2O]+ | 342.1162 | 1 | Isomaltose | C12H22O11 | DW |
| 1.67_325.1129 | 1.67 | 325.1129 | [M+H-H2O]+ | 342.1162 | 1 | Maltose | C12H22O11 | DW |
| 1.67_325.1129 | 1.67 | 325.1129 | [M+H-H2O]+ | 342.1162 | 1 | Lactulose | C12H22O11 | DW |
| 1.67_325.1129 | 1.67 | 325.1129 | [M+H-H2O]+ | 342.1162 | 1 | Epimelibiose | C12H22O11 | DW |
| 1.67_325.1129 | 1.67 | 325.1129 | [M+H-H2O]+ | 342.1162 | 1 | Galactinol (1-&alpha;-d-galactosyl-myo-inositol) | C12H22O11 | DW |
| 1.67_325.1129 | 1.67 | 325.1129 | [M+H-H2O]+ | 342.1162 | 1 | Melibiose | C12H22O11 | DW |
| 1.67_325.1129 | 1.67 | 325.1129 | [M+H-H2O]+ | 342.1162 | 1 | α,α-Trehalose | C12H22O11 | DW |
| 1.67_325.1129 | 1.67 | 325.1129 | [M+H-H2O]+ | 342.1162 | 1 | Cellobiose | C12H22O11 | DW |
| 1.67_325.1129 | 1.67 | 325.1129 | [M+H-H2O]+ | 342.1162 | 1 | Laminaribiose | C12H22O11 | DW |
| 1.67_325.1129 | 1.67 | 325.1129 | [M+H-H2O]+ | 342.1162 | 1 | Nigerose (Sakebiose) | C12H22O11 | DW |
| 1.67_325.1129 | 1.67 | 325.1129 | [M+H-H2O]+ | 342.1162 | 1 | D-(+)-Cellobiose | C12H22O11 | DW |
| 1.67_325.1129 | 1.67 | 325.1129 | [M+Li]+ | 318.0957 | 3 | Organosulfur derivative (structurally similar to chlorpromazine) | C17H19ClN2S | DW |
| 1.67_325.1129 | 1.67 | 325.1129 | [M+Li]+ | 318.0997 | 8 | Isopentyl pyrophosphate | C10H24O7P2 | DW |
| 1.67_325.1129 | 1.67 | 325.1129 | [M+CH3OH+H]+ | 292.0768 | 8 | 3-(2-chloro-10H-phenothiazin-10-yl)propan-1-amine | C15H13D2ClN2S | DW |
| 5.21_337.2678 | 5.21 | 337.2678 | [M+H-2H2O]+ | 372.2777 | 8 | Steroid derivative (structurally similar to finasteride) | C23H36N2O2 | DW |
| 7.43_339.2898 | 7.43 | 339.2898 | [M+H-H2O]+ | 356.2926 | 0 | Heneicosanedioic acid | C21H40O4 | BW |
| 7.43_339.2898 | 7.43 | 339.2898 | [M+H-H2O]+ | 356.2927 | 0 | MG(0:0/18:1(11Z)/0:0) | C21H40O4 | BW |
| 7.43_339.2898 | 7.43 | 339.2898 | [M+H-H2O]+ | 356.2927 | 0 | MG(0:0/18:1(9Z)/0:0) | C21H40O4 | BW |
| 7.43_339.2898 | 7.43 | 339.2898 | [M+H-H2O]+ | 356.2927 | 0 | MG(18:1(11Z)/0:0/0:0) | C21H40O4 | BW |
| 7.43_339.2898 | 7.43 | 339.2898 | [M+H-H2O]+ | 356.2927 | 0 | MG(18:1(9Z)/0:0/0:0) | C21H40O4 | BW |
| 7.43_339.2898 | 7.43 | 339.2898 | [M+H-H2O]+ | 356.2927 | 0 | MG(18:1(11E)/0:0/0:0)[rac] | C21H40O4 | BW |
| 7.43_339.2898 | 7.43 | 339.2898 | [M+H-H2O]+ | 356.2927 | 0 | MG(18:1(9Z)/0:0/0:0)[rac] | C21H40O4 | BW |
| 7.43_339.2898 | 7.43 | 339.2898 | [M+CH3OH+H]+ | 306.2559 | 1 | 18-methyl-8Z,11Z,14Z-nonadecatrienoic acid | C20H34O2 | BW |
| 7.43_339.2898 | 7.43 | 339.2898 | [M+CH3OH+H]+ | 306.2559 | 1 | 5,8,11-eicosatrienoic acid | C20H34O2 | BW |
| 7.43_339.2898 | 7.43 | 339.2898 | [M+CH3OH+H]+ | 306.2559 | 1 | Dihomo-β;-Linolenic Acid | C20H34O2 | BW |
| 7.43_339.2898 | 7.43 | 339.2898 | [M+CH3OH+H]+ | 306.2559 | 1 | 11,14,17-eicosatrienoic acid | C20H34O2 | BW |
| 7.43_339.2898 | 7.43 | 339.2898 | [M+CH3OH+H]+ | 306.2559 | 1 | Podocarpric acid | C20H34O2 | BW |
| 7.43_339.2898 | 7.43 | 339.2898 | [M+CH3OH+H]+ | 306.2559 | 1 | 11(Z),14(Z),17(Z)-Eicosatrienoic Acid | C20H34O2 | BW |
| 7.43_339.2898 | 7.43 | 339.2898 | [M+CH3OH+H]+ | 306.2559 | 1 | 2E,4E,8Z-eicosatrienoic acid | C20H34O2 | BW |
| 7.43_339.2898 | 7.43 | 339.2898 | [M+CH3OH+H]+ | 306.2559 | 1 | 5(Z),11(Z),14(Z)-Eicosatrienoic acid | C20H34O2 | BW |
| 7.43_339.2898 | 7.43 | 339.2898 | [M+CH3OH+H]+ | 306.2559 | 1 | 5(Z),8(Z),11(Z)-Eicosatrienoic Acid | C20H34O2 | BW |
| 7.43_339.2898 | 7.43 | 339.2898 | [M+CH3OH+H]+ | 306.2559 | 1 | 7,10,13-Eicosatrienoic acid | C20H34O2 | BW |
| 7.43_339.2898 | 7.43 | 339.2898 | [M+CH3OH+H]+ | 306.2559 | 1 | 7,11,14-Eicosatrienoic acid | C20H34O2 | BW |
| 7.43_339.2898 | 7.43 | 339.2898 | [M+CH3OH+H]+ | 306.2559 | 1 | 7Z,10Z,13Z-eicosatrienoic acid | C20H34O2 | BW |
| 7.43_339.2898 | 7.43 | 339.2898 | [M+CH3OH+H]+ | 306.2559 | 1 | 7Z,11Z,14E-eicosatrienoic acid | C20H34O2 | BW |
| 7.43_339.2898 | 7.43 | 339.2898 | [M+CH3OH+H]+ | 306.2559 | 1 | 7Z,11Z,14Z-eicosatrienoic acid | C20H34O2 | BW |
| 7.43_339.2898 | 7.43 | 339.2898 | [M+CH3OH+H]+ | 306.2559 | 1 | 8Z,12E,14Z-eicosatrienoic acid | C20H34O2 | BW |
| 7.43_339.2898 | 7.43 | 339.2898 | [M+CH3OH+H]+ | 306.2559 | 1 | Isoprenoid derivative (structurally similar to plaunotol) | C20H34O2 | BW |
| 7.43_339.2898 | 7.43 | 339.2898 | [M+CH3OH+H]+ | 306.2559 | 1 | Eicosatrienoic acid | C20H34O2 | BW |
| 7.43_339.2898 | 7.43 | 339.2898 | [M+CH3OH+H]+ | 306.2559 | 1 | Larixol | C20H34O2 | BW |
| 7.43_339.2898 | 7.43 | 339.2898 | [M+CH3OH+H]+ | 306.2559 | 1 | Linolenic Acid ethyl ester | C20H34O2 | BW |
| 7.43_339.2898 | 7.43 | 339.2898 | [M+CH3OH+H]+ | 306.2559 | 1 | 9(Z),11(E),13(E)-Octadecatrienoic Acid ethyl ester | C20H34O2 | BW |
| 7.43_339.2898 | 7.43 | 339.2898 | [M+CH3OH+H]+ | 306.2559 | 1 | Pinolenic Acid ethyl ester | C20H34O2 | BW |
| 7.43_339.2898 | 7.43 | 339.2898 | [M+CH3OH+H]+ | 306.2559 | 1 | 5(Z),8(Z),14(Z)-Eicosatrienoic Acid | C20H34O2 | BW |
| 7.43_339.2898 | 7.43 | 339.2898 | [M+CH3OH+H]+ | 306.2559 | 1 | (5Z,9E,14Z)-icosa-5,9,14-trienoic acid | C20H34O2 | BW |
| 7.43_339.2898 | 7.43 | 339.2898 | [M+CH3OH+H]+ | 306.2559 | 1 | Oncobic acid | C20H34O2 | BW |
| 7.43_339.2898 | 7.43 | 339.2898 | [M+CH3OH+H]+ | 306.2559 | 1 | 3Z,6Z,9Z-Octadecatrienyl acetate | C20H34O2 | BW |
| 7.43_339.2898 | 7.43 | 339.2898 | [M+CH3OH+H]+ | 306.2559 | 1 | 9Z,12Z,15Z-Octadecatrienyl acetate | C20H34O2 | BW |
| 7.43_339.2898 | 7.43 | 339.2898 | [M+CH3OH+H]+ | 306.2559 | 1 | Isoprenoid derivative (structurally similar to plaunotol0 | C20H34O2 | BW |
| 7.43_339.2898 | 7.43 | 339.2898 | [M+Li]+ | 332.2715 | 6 | 4,7,10,13-docosatetraenoic acid | C22H36O2 | BW |
| 7.43_339.2898 | 7.43 | 339.2898 | [M+Li]+ | 332.2715 | 6 | Adrenic Acid | C22H36O2 | BW |
| 7.43_339.2898 | 7.43 | 339.2898 | [M+Li]+ | 332.2715 | 6 | 8,12,16,19-docosatetraenoic acid | C22H36O2 | BW |
| 7.43_339.2898 | 7.43 | 339.2898 | [M+Li]+ | 332.2715 | 6 | Arachidonic acid ethyl ester | C22H36O2 | BW |
| 7.43_339.2898 | 7.43 | 339.2898 | [M+Li]+ | 332.2715 | 6 | cis-4,10,13,16-Docosatetraenoic Acid | C22H36O2 | BW |
| 7.43_339.2898 | 7.43 | 339.2898 | [M+Li]+ | 332.2715 | 6 | omega;-3 Arachidonic Acid ethyl ester | C22H36O2 | BW |
| 7.43_339.2898 | 7.43 | 339.2898 | [M+Li]+ | 332.2715 | 6 | (&plusmn;)-CP 47,497-C8-homolog | C22H36O2 | BW |
| 7.43_339.2898 | 7.43 | 339.2898 | [M+Li]+ | 332.2715 | 6 | 10-[3]-ladderane-decanoic acid | C22H36O2 | BW |
| 7.43_339.2898 | 7.43 | 339.2898 | [M+Li]+ | 332.2715 | 6 | Adrenic acid | C22H36O2 | BW |
| 7.43_339.2898 | 7.43 | 339.2898 | [M+Li]+ | 332.2715 | 6 | 3,7,11,15-Tetramethyl-2E,6E,10E,14-hexadecatetraenyl acetate | C22H36O2 | BW |
| 7.43_339.2898 | 7.43 | 339.2898 | [M+Na]+ | 316.2977 | 8 | Chimyl alcohol | C19H40O3 | BW |
| 6.14_349.3104 | 6.14 | 349.3104 | [M+3H]3+ | 1044.9085 | 0 | TG(22:1(13Z)/22:3(10Z,13Z,16Z)/22:3(10Z,13Z,16Z))[iso3] | C69H120O6 | BW |
| 6.14_349.3104 | 6.14 | 349.3104 | [M+3H]3+ | 1044.9085 | 0 | TG(22:0/22:3(10Z,13Z,16Z)/22:4(7Z,10Z,13Z,16Z))[iso6] | C69H120O6 | BW |
| 6.14_349.3104 | 6.14 | 349.3104 | [M+3H]3+ | 1044.9085 | 0 | TG(22:1(13Z)/22:1(13Z)/22:5(7Z,10Z,13Z,16Z,19Z))[iso3] | C69H120O6 | BW |
| 6.14_349.3104 | 6.14 | 349.3104 | [M+3H]3+ | 1044.9085 | 0 | TG(22:0/22:1(13Z)/22:6(4Z,7Z,10Z,13Z,16Z,19Z))[iso6] | C69H120O6 | BW |
| 6.14_349.3104 | 6.14 | 349.3104 | [M+3H]3+ | 1044.9085 | 0 | TG(22:3(10Z,13Z,16Z)/22:2(13Z,16Z)/22:2(13Z,16Z))[iso3] | C69H120O6 | BW |
| 6.14_349.3104 | 6.14 | 349.3104 | [M+3H]3+ | 1044.9085 | 0 | TG(22:1(13Z)/22:2(13Z,16Z)/22:4(7Z,10Z,13Z,16Z))[iso6] | C69H120O6 | BW |
| 6.14_349.3104 | 6.14 | 349.3104 | [M+3H]3+ | 1044.9085 | 0 | TG(22:0/22:2(13Z,16Z)/22:5(7Z,10Z,13Z,16Z,19Z))[iso6] | C69H120O6 | BW |
| 6.14_349.3104 | 6.14 | 349.3104 | [M+H-2H2O]+ | 384.3239 | 2 | Tricosanedioic acid | C23H44O4 | BW |
| 6.14_349.3104 | 6.14 | 349.3104 | [M+H-2H2O]+ | 384.3240 | 2 | MG(0:0/20:1(11Z)/0:0) | C23H44O4 | BW |
| 6.14_349.3104 | 6.14 | 349.3104 | [M+H-2H2O]+ | 384.3240 | 2 | MG(20:1(11Z)/0:0/0:0) | C23H44O4 | BW |
| 6.14_349.3104 | 6.14 | 349.3104 | [M+2H+Na]3+ | 1022.9241 | 3 | TG(20:0/22:1(13Z)/22:3(10Z,13Z,16Z))[iso6] | C67H122O6 | BW |
| 6.14_349.3104 | 6.14 | 349.3104 | [M+2H+Na]3+ | 1022.9241 | 3 | TG(20:1(11Z)/22:0/22:3(10Z,13Z,16Z))[iso6] | C67H122O6 | BW |
| 6.14_349.3104 | 6.14 | 349.3104 | [M+2H+Na]3+ | 1022.9241 | 3 | TG(20:4(5Z,8Z,11Z,14Z)/22:0/22:0)[iso3] | C67H122O6 | BW |
| 6.14_349.3104 | 6.14 | 349.3104 | [M+2H+Na]3+ | 1022.9241 | 3 | TG(20:2(11Z,14Z)/22:1(13Z)/22:1(13Z))[iso3] | C67H122O6 | BW |
| 6.14_349.3104 | 6.14 | 349.3104 | [M+2H+Na]3+ | 1022.9241 | 3 | TG(20:3(8Z,11Z,14Z)/22:0/22:1(13Z))[iso6] | C67H122O6 | BW |
| 6.14_349.3104 | 6.14 | 349.3104 | [M+2H+Na]3+ | 1022.9241 | 3 | TG(20:0/22:0/22:4(7Z,10Z,13Z,16Z))[iso6] | C67H122O6 | BW |
| 6.14_349.3104 | 6.14 | 349.3104 | [M+2H+Na]3+ | 1022.9241 | 3 | TG(20:1(11Z)/22:1(13Z)/22:2(13Z,16Z))[iso6] | C67H122O6 | BW |
| 6.14_349.3104 | 6.14 | 349.3104 | [M+2H+Na]3+ | 1022.9241 | 3 | TG(20:2(11Z,14Z)/22:0/22:2(13Z,16Z))[iso6] | C67H122O6 | BW |
| 6.14_349.3104 | 6.14 | 349.3104 | [M+2H+Na]3+ | 1022.9241 | 3 | TG(20:0/22:2(13Z,16Z)/22:2(13Z,16Z))[iso3] | C67H122O6 | BW |
| 6.14_349.3104 | 6.14 | 349.3104 | [M+2H+Na]3+ | 1022.9241 | 3 | TG(21:0/21:0/22:4(7Z,10Z,13Z,16Z))[iso3] | C67H122O6 | BW |
| 6.14_349.3104 | 6.14 | 349.3104 | [M+Na]+ | 326.3185 | 7 | 19-methyl-eicosanoic acid | C21H42O2 | BW |
| 6.14_349.3104 | 6.14 | 349.3104 | [M+Na]+ | 326.3185 | 7 | heneicosanoic acid | C21H42O2 | BW |
| 6.14_349.3104 | 6.14 | 349.3104 | [M+Na]+ | 326.3185 | 7 | (+)-18-methyl-eicosanoic acid | C21H42O2 | BW |
| 6.14_349.3104 | 6.14 | 349.3104 | [M+Na]+ | 326.3185 | 7 | 2,6-dimethyl-nonadecanoic acid | C21H42O2 | BW |
| 6.14_349.3104 | 6.14 | 349.3104 | [M+Na]+ | 326.3185 | 7 | Homophytanic acid | C21H42O2 | BW |
| 2.34_353.228 | 2.34 | 353.228 | [M+2Na]2+ | 660.4754 | 3 | DG(20:5(5Z,8Z,11Z,14Z,17Z)/20:5(5Z,8Z,11Z,14Z,17Z)/0:0) | C43H64O5 | BW |
| 2.34_353.228 | 2.34 | 353.228 | [M+2Na]2+ | 660.4754 | 3 | DG(18:4(6Z,9Z,12Z,15Z)/22:6(4Z,7Z,10Z,13Z,16Z,19Z)/0:0) | C43H64O5 | BW |
| 2.34_353.228 | 2.34 | 353.228 | [M+2Na]2+ | 660.4754 | 3 | DG(22:6(4Z,7Z,10Z,13Z,16Z,19Z)/18:4(6Z,9Z,12Z,15Z)/0:0) | C43H64O5 | BW |
| 2.34_353.228 | 2.34 | 353.228 | [M+Na]+ | 330.2406 | 5 | 11,12,13-trihydroxy-9-octadecenoic acid | C18H34O5 | BW |
| 2.34_353.228 | 2.34 | 353.228 | [M+Na]+ | 330.2406 | 5 | 9,10,13-trihydroxy-11-octadecenoic acid | C18H34O5 | BW |
| 2.34_353.228 | 2.34 | 353.228 | [M+Na]+ | 330.2406 | 5 | 9,12,13-trihydroxy-10-octadecenoic acid | C18H34O5 | BW |
| 2.34_353.228 | 2.34 | 353.228 | [M+Na]+ | 330.2406 | 5 | 5,8,12-trihydroxy-9-octadecenoic acid | C18H34O5 | BW |
| 2.34_353.228 | 2.34 | 353.228 | [M+Na]+ | 330.2406 | 5 | 9,10,18-trihydroxy-12-octadecenoic acid | C18H34O5 | BW |
| 2.34_353.228 | 2.34 | 353.228 | [M+Na]+ | 330.2406 | 5 | (+)-9,10,18-trihydroxy-12Z-octadecenoic acid | C18H34O5 | BW |
| 2.34_353.228 | 2.34 | 353.228 | [M+Na]+ | 330.2406 | 5 | 9S,10S,11R-trihydroxy-12Z-octadecenoic acid | C18H34O5 | BW |
| 2.34_353.228 | 2.34 | 353.228 | [M+Na]+ | 330.2406 | 5 | 9S,12S,13S-trihydroxy-10E-octadecenoic acid | C18H34O5 | BW |
| 2.34_353.228 | 2.34 | 353.228 | [M+Li]+ | 346.2144 | 6 | Steroid derivative (structurally similar to corticosterone) | C21H30O4 | BW |
| 2.34_353.228 | 2.34 | 353.228 | [M+Li]+ | 346.2144 | 6 | Steroid derivative (structurally similar to 21-deoxycortisol) | C21H30O4 | BW |
| 2.34_353.228 | 2.34 | 353.228 | [M+Li]+ | 346.2144 | 6 | 1α;,21-dihydroxy-20-oxo-22,23,24,25,26,27-hexanorvitamin D3 / 1α;,21-dihydroxy-20-oxo-22,23,24,25,26,27-hexanorcholecalciferol | C21H30O4 | BW |
| 2.34_353.228 | 2.34 | 353.228 | [M+Li]+ | 346.2144 | 6 | Steroid derivative (structurally similar to 13,14-dehydro-15-cyclohexyl carbaprostacyclin) | C21H30O4 | BW |
| 2.34_353.228 | 2.34 | 353.228 | [M+Li]+ | 346.2144 | 6 | algestone | C21H30O4 | BW |
| 2.34_353.228 | 2.34 | 353.228 | [M+Li]+ | 346.2144 | 6 | 21-Hydroxy-5b-pregnane-3,11,20-trione | C21H30O4 | BW |
| 2.34_353.228 | 2.34 | 353.228 | [M+Li]+ | 346.2144 | 6 | 21-Hydroxy-5β;-pregnane-3,11,20-trione | C21H30O4 | BW |
| 2.34_353.228 | 2.34 | 353.228 | [M+Li]+ | 346.2144 | 6 | α,11-Dihydroxy-D9-tetrahydrocannabinol | C21H30O4 | BW |
| 2.34_353.228 | 2.34 | 353.228 | [M+Li]+ | 346.2144 | 6 | Steroid derivative (structurally similar to cortexolone) | C21H30O4 | BW |
| 2.34_353.228 | 2.34 | 353.228 | [M+Li]+ | 346.2144 | 6 | Steroid derivative (structurally similar to corticosterone) | C21H30O4 | BW |
| 2.34_353.228 | 2.34 | 353.228 | [M+Li]+ | 346.2144 | 6 | Steroid derivative (structurally similar to 21-deoxycortisol) | C21H30O4 | BW |
| 2.34_353.228 | 2.34 | 353.228 | [M+H-2H2O]+ | 388.2379 | 8 | Palmitoleoyl 3-carbacyclic Phosphatidic Acid | C20H37O5P | BW |
| 2.23_361.2844 | 2.23 | 361.2844 | [M+2H]2+ | 720.5543 | 0 | PC(12:0/19:0)[U] | C39H79NO8P | DW |
| 2.23_361.2844 | 2.23 | 361.2844 | [M+2H]2+ | 720.5543 | 0 | PC(13:0/18:0)[U] | C39H79NO8P | DW |
| 2.23_361.2844 | 2.23 | 361.2844 | [M+2H]2+ | 720.5543 | 0 | PC(14:0/17:0)[U] | C39H79NO8P | DW |
| 2.23_361.2844 | 2.23 | 361.2844 | [M+2H]2+ | 720.5543 | 0 | PC(15:0/16:0)[U] | C39H79NO8P | DW |
| 2.23_361.2844 | 2.23 | 361.2844 | [M+2H]2+ | 720.5543 | 0 | PC(16:0/15:0)[U] | C39H79NO8P | DW |
| 2.23_361.2844 | 2.23 | 361.2844 | [M+2H]2+ | 720.5543 | 0 | PC(17:0/14:0)[U] | C39H79NO8P | DW |
| 2.23_361.2844 | 2.23 | 361.2844 | [M+2H]2+ | 720.5543 | 0 | PC(18:0/13:0)[U] | C39H79NO8P | DW |
| 2.23_361.2844 | 2.23 | 361.2844 | [M+CH3OH+H]+ | 328.2515 | 1 | Steroid derivative (structurally similar to stanozolol) | C21H32N2O | DW |
| 2.23_361.2844 | 2.23 | 361.2844 | [M+CH3OH+H]+ | 328.2515 | 1 | Steroid derivative (structurally similar to 17-epistanozolol) | C21H32N2O | DW |
| 2.46_363.3007 | 2.46 | 363.3007 | NC |  |  |  |  | DW |
| 2.25_365.316 | 2.25 | 365.316 | [M+K]+ | 326.3549 | 5 | Behenyl alcohol | C22H46O | DW |
| 2.2_377.2804 | 2.20 | 377.2804 | [M+CH3OH+H]+ | 344.2464 | 1 | Steroid derivative (structurally similar to 3\'-Hydroxystanozolol) | C21H32N2O2 | DW |
| 2.2_377.2804 | 2.20 | 377.2804 | [M+CH3OH+H]+ | 344.2464 | 1 | Steroid derivative (structurally similar to 4β;-Hydroxystanozolol) | C21H32N2O2 | DW |
| 2.2_377.2804 | 2.20 | 377.2804 | [M+CH3OH+H]+ | 344.2464 | 1 | Steroid derivative (structurally similar to 16β;-Hydroxystanozolol) | C21H32N2O2 | DW |
| 2.2_377.2804 | 2.20 | 377.2804 | [M+CH3OH+H]+ | 344.2464 | 1 | Steroid derivative (structurally similar to 16α;-Hydroxystanozolol) | C21H32N2O2 | DW |
| 2.2_377.2804 | 2.20 | 377.2804 | [M+CH3OH+H]+ | 344.2464 | 1 | Steroid derivative (structurally similar to 3\'-Hydroxy-17-epistanozolol) | C21H32N2O2 | DW |
| 2.2_377.2804 | 2.20 | 377.2804 | [M+CH3OH+H]+ | 344.2464 | 1 | Steroid derivative (structurally similar to 16α;-Hydroxy-17-epistanozolol) | C21H32N2O2 | DW |
| 2.2_377.2804 | 2.20 | 377.2804 | [M+H]+ | 376.2742 | 2 | Phytyl phosphate | C20H41O4P | DW |
| 2.2_377.2804 | 2.20 | 377.2804 | [M+K]+ | 338.3185 | 3 | 2,4-dimethyl-2-eicosenoic acid | C22H42O2 | DW |
| 2.2_377.2804 | 2.20 | 377.2804 | [M+K]+ | 338.3185 | 3 | trans-brassidic acid | C22H42O2 | DW |
| 2.2_377.2804 | 2.20 | 377.2804 | [M+K]+ | 338.3185 | 3 | 11-docosenoic acid | C22H42O2 | DW |
| 2.2_377.2804 | 2.20 | 377.2804 | [M+K]+ | 338.3185 | 3 | 15-docosenoic acid | C22H42O2 | DW |
| 2.2_377.2804 | 2.20 | 377.2804 | [M+K]+ | 338.3185 | 3 | 15Z-docosenoic acid | C22H42O2 | DW |
| 2.2_377.2804 | 2.20 | 377.2804 | [M+K]+ | 338.3185 | 3 | 19Z-docosenoic acid | C22H42O2 | DW |
| 2.2_377.2804 | 2.20 | 377.2804 | [M+K]+ | 338.3185 | 3 | 5Z-docosenoic acid | C22H42O2 | DW |
| 2.2_377.2804 | 2.20 | 377.2804 | [M+K]+ | 338.3185 | 3 | cis-cetoleic acid | C22H42O2 | DW |
| 2.2_377.2804 | 2.20 | 377.2804 | [M+K]+ | 338.3185 | 3 | 13(Z)-Docosenoic Acid | C22H42O2 | DW |
| 2.2_377.2804 | 2.20 | 377.2804 | [M+K]+ | 338.3185 | 3 | 11Z-Eicosenyl acetate | C22H42O2 | DW |
| 2.2_377.2804 | 2.20 | 377.2804 | [M+Li]+ | 370.2620 | 6 | Catecholamine derivative (structurally similar to methyldopexamine) | C23H34N2O2 | DW |
| 6.58_377.3415 | 6.58 | 377.3415 | [M+H-2H2O]+ | 412.3553 | 2 | MG(0:0/22:1(13Z)/0:0) | C25H48O4 | BW |
| 6.58_377.3415 | 6.58 | 377.3415 | [M+H-2H2O]+ | 412.3553 | 2 | MG(22:1(13Z)/0:0/0:0) | C25H48O4 | BW |
| 6.58_377.3415 | 6.58 | 377.3415 | [M+Na]+ | 354.3498 | 6 | 21-methyl-docosanoic acid | C23H46O2 | BW |
| 6.58_377.3415 | 6.58 | 377.3415 | [M+Na]+ | 354.3498 | 6 | tricosanoic acid | C23H46O2 | BW |
| 6.58_377.3415 | 6.58 | 377.3415 | [M+Na]+ | 354.3498 | 6 | (+)-20-methyl-docosanoic acid | C23H46O2 | BW |
| 2.3_379.2931 | 2.30 | 379.2931 | [M+Li]+ | 372.2777 | 1 | Steroid derivative (structurally similar to finasteride) | C23H36N2O2 | DW |
| 2.3_379.2931 | 2.30 | 379.2931 | [M+2H]2+ | 756.5751 | 4 | MGDG(16:0/18:1(9Z)) | C43H80O10 | DW |
| 2.24_379.294 | 2.24 | 379.294 | [M+Li]+ | 372.2777 | 0 | Steroid derivative (structurally similar to finasteride) | C23H36N2O2 | DW |
| 2.24_379.294 | 2.24 | 379.294 | [M+2H]2+ | 756.5751 | 2 | MGDG(16:0/18:1(9Z)) | C43H80O10 | DW |
| 2.24_379.294 | 2.24 | 379.294 | [M+Na]+ | 356.3079 | 8 | (-)-Ceriferol 1 | C25H40O | DW |
| 2.24_379.294 | 2.24 | 379.294 | [M+K]+ | 340.3341 | 8 | 19-methyl-heneicosanoic acid | C22H44O2 | DW |
| 2.24_379.294 | 2.24 | 379.294 | [M+K]+ | 340.3341 | 8 | 3-methyl-heneicosanoic acid | C22H44O2 | DW |
| 2.24_379.294 | 2.24 | 379.294 | [M+K]+ | 340.3341 | 8 | Docosanoic acid | C22H44O2 | DW |
| 2.24_379.294 | 2.24 | 379.294 | [M+K]+ | 340.3341 | 8 | Calcifediol | C22H44O2 | DW |
| 2.24_379.294 | 2.24 | 379.294 | [M+K]+ | 340.3341 | 8 | Isobehenic acid | C22H44O2 | DW |
| 2.24_379.294 | 2.24 | 379.294 | [M+K]+ | 340.3341 | 8 | Eicosyl acetate | C22H44O2 | DW |
| 2.38_381.3093 | 2.38 | 381.3093 | [M+Na]+ | 358.3236 | 9 | 3,7,11,15,19-Pentamethyleicosa-2E,6E,10E,14E,18-pentaen-1-ol | C25H42O | DW |
| 2.38_381.3093 | 2.38 | 381.3093 | [M+K]+ | 342.3498 | 9 | 15-methyl-1,2-heneicosanediol | C22H46O2 | DW |
| 2.46_381.3096 | 2.46 | 381.3096 | [M+Na]+ | 358.3236 | 8 | 3,7,11,15,19-Pentamethyleicosa-2E,6E,10E,14E,18-pentaen-1-ol | C25H42O | DW |
| 2.46_381.3096 | 2.46 | 381.3096 | [M+K]+ | 342.3498 | 8 | 15-methyl-1,2-heneicosanediol | C22H46O2 | DW |
| 2.08_393.2754 | 2.08 | 393.2754 | [M+K]+ | 354.3134 | 2 | 10-oxo-docosanoic acid | C22H42O3 | DW |
| 2.08_393.2754 | 2.08 | 393.2754 | [M+K]+ | 354.3134 | 2 | 2-oxo-docosanoic acid | C22H42O3 | DW |
| 2.08_393.2754 | 2.08 | 393.2754 | [M+K]+ | 354.3134 | 2 | 21-oxo-docosanoic acid | C22H42O3 | DW |
| 2.08_393.2754 | 2.08 | 393.2754 | [M+K]+ | 354.3134 | 2 | 3-oxo-docosanoic acid | C22H42O3 | DW |
| 2.08_393.2754 | 2.08 | 393.2754 | [M+K]+ | 354.3134 | 2 | 4-oxo-docosanoic acid | C22H42O3 | DW |
| 2.22_395.2897 | 2.22 | 395.2897 | [M+Li]+ | 388.2726 | 2 | Steroid derivative (structurally similar to o-Hydroxyfinasteride) | C23H36N2O3 | DW |
| 2.22_395.2897 | 2.22 | 395.2897 | [M+Na]+ | 372.3028 | 5 | (+)-Dysideapalaunic acid | C25H40O2 | DW |
| 2.22_395.2897 | 2.22 | 395.2897 | [M+K]+ | 356.3290 | 6 | 2-hydroxy behenic | C22H44O3 | DW |
| 2.22_395.2897 | 2.22 | 395.2897 | [M+K]+ | 356.3290 | 6 | (+)-3-hydroxy behenic | C22H44O3 | DW |
| 2.22_395.2897 | 2.22 | 395.2897 | [M+K]+ | 356.3290 | 6 | omega-hydroxy behenic | C22H44O3 | DW |
| 2.22_395.2897 | 2.22 | 395.2897 | [M+K]+ | 356.3290 | 6 | 13-hydroxy-docosanoic acid | C22H44O3 | DW |
| 6.96_413.3784 | 6.96 | 413.3784 | [M+H-H2O]+ | 430.3811 | 0 | toxisterol3 B1 / (5E)-(10R)-10-ethoxy-10,19-dihydrovitamin D3 / (5E)-(10R)-10-ethoxy-10,19-dihydrocholecalciferol | C29H50O2 | DW |
| 6.96_413.3784 | 6.96 | 413.3784 | [M+H-H2O]+ | 430.3811 | 0 | toxisterol3 B2 / (5E)-(10S)-10-ethoxy-10,19-dihydrovitamin D3 / (5E)-(10S)-10-ethoxy-10,19-dihydrocholecalciferol | C29H50O2 | DW |
| 6.96_413.3784 | 6.96 | 413.3784 | [M+H-H2O]+ | 430.3811 | 0 | toxisterol3 B3 / (10R)-10-ethoxy-10,19-dihydrovitamin D3 / (10R)-10-ethoxy-10,19-dihydrocholecalciferol | C29H50O2 | DW |
| 6.96_413.3784 | 6.96 | 413.3784 | [M+H-H2O]+ | 430.3811 | 0 | (+)-α;-Tocopherol | C29H50O2 | DW |
| 6.96_413.3784 | 6.96 | 413.3784 | [M+H-H2O]+ | 430.3811 | 0 | 4β-(hydroxymethyl)-4α-methyl-5α-cholest-7-en-3β-ol | C29H50O2 | DW |
| 6.96_413.3784 | 6.96 | 413.3784 | [M+H-H2O]+ | 430.3811 | 0 | 4α-hydroxymethyl-4β-methyl-5α-cholesta-8-en-3β-ol | C29H50O2 | DW |
| 6.96_413.3784 | 6.96 | 413.3784 | [M+H-H2O]+ | 430.3811 | 0 | 4α,24β-Dimethyl-5α-cholest-22-en-3β-4β-diol | C29H50O2 | DW |
| 6.96_413.3784 | 6.96 | 413.3784 | [M+H-H2O]+ | 430.3811 | 0 | 4α-hydroxymethyl-4β-methyl-5α-cholesta-8-en-3β-ol | C29H50O2 | DW |
| 6.96_413.3784 | 6.96 | 413.3784 | [M+H]+ | 412.3705 | 1 | 14-Demethyl-lanosterol | C29H48O | DW |
| 6.96_413.3784 | 6.96 | 413.3784 | [M+H]+ | 412.3705 | 1 | 4α;-methyl,24-methylene-cholest-7-en-3β;-ol | C29H48O | DW |
| 6.96_413.3784 | 6.96 | 413.3784 | [M+H]+ | 412.3705 | 1 | 24-methylene,25-methylcholesta-5-en-3β;-ol | C29H48O | DW |
| 6.96_413.3784 | 6.96 | 413.3784 | [M+H]+ | 412.3705 | 1 | 24-methylene-25-methyl-cholest-7-en-3β;-ol | C29H48O | DW |
| 6.96_413.3784 | 6.96 | 413.3784 | [M+H]+ | 412.3705 | 1 | stigmasta-5,7-dien-3β;-ol | C29H48O | DW |
| 6.96_413.3784 | 6.96 | 413.3784 | [M+H]+ | 412.3705 | 1 | 24Z-ethylidene-cholest-5-en-3-ol | C29H48O | DW |
| 6.96_413.3784 | 6.96 | 413.3784 | [M+H]+ | 412.3705 | 1 | stigmasta-5,25-dien-3β;-ol | C29H48O | DW |
| 6.96_413.3784 | 6.96 | 413.3784 | [M+H]+ | 412.3705 | 1 | stigmasta-7,22E-dien-3β;-ol | C29H48O | DW |
| 6.96_413.3784 | 6.96 | 413.3784 | [M+H]+ | 412.3705 | 1 | poriferasta-5,22E-dien-3β;-ol | C29H48O | DW |
| 6.96_413.3784 | 6.96 | 413.3784 | [M+H]+ | 412.3705 | 1 | 24-ethylcholesta-5,24-dien-3β;-ol | C29H48O | DW |
| 6.96_413.3784 | 6.96 | 413.3784 | [M+H]+ | 412.3705 | 1 | 24-ethyl-cholesta-7,24-dien-3β;-ol | C29H48O | DW |
| 6.96_413.3784 | 6.96 | 413.3784 | [M+H]+ | 412.3705 | 1 | stigmasta-7,25-dien-3β;-ol | C29H48O | DW |
| 6.96_413.3784 | 6.96 | 413.3784 | [M+H]+ | 412.3705 | 1 | poriferasta-7,22E-dien-3β;-ol | C29H48O | DW |
| 6.96_413.3784 | 6.96 | 413.3784 | [M+H]+ | 412.3705 | 1 | 24-ethylcholesta-5,23E-dien-3β;-ol | C29H48O | DW |
| 6.96_413.3784 | 6.96 | 413.3784 | [M+H]+ | 412.3705 | 1 | stigmasta-8,22E-dien-3β;-ol | C29H48O | DW |
| 6.96_413.3784 | 6.96 | 413.3784 | [M+H]+ | 412.3705 | 1 | Isofucosterol | C29H48O | DW |
| 6.96_413.3784 | 6.96 | 413.3784 | [M+H]+ | 412.3705 | 1 | Fucosterol | C29H48O | DW |
| 6.96_413.3784 | 6.96 | 413.3784 | [M+H]+ | 412.3705 | 1 | Vitamin D5 | C29H48O | DW |
| 6.96_413.3784 | 6.96 | 413.3784 | [M+H]+ | 412.3705 | 1 | Stigmasterol | C29H48O | DW |
| 6.96_413.3784 | 6.96 | 413.3784 | [M+H]+ | 412.3705 | 1 | Fucosterol | C29H48O | DW |
| 6.96_413.3784 | 6.96 | 413.3784 | [M+H]+ | 412.3705 | 1 | Provitamin D5 | C29H48O | DW |
| 6.96_413.3784 | 6.96 | 413.3784 | [M+H]+ | 412.3705 | 1 | 4,14-Dimethylcholest-8,24-dien-3β-ol | C29H48O | DW |
| 6.96_413.3784 | 6.96 | 413.3784 | [M+H]+ | 412.3705 | 1 | 14-demethyllanosterol | C29H48O | DW |
| 6.96_413.3784 | 6.96 | 413.3784 | [M+H]+ | 412.3705 | 1 | 22,23-Methylene-24-methyl-cholest-5-en-3β-ol | C29H48O | DW |
| 6.96_413.3784 | 6.96 | 413.3784 | [M+H]+ | 412.3705 | 1 | 23-methyl,24-methylene-cholest-5-en-3β-ol | C29H48O | DW |
| 6.96_413.3784 | 6.96 | 413.3784 | [M+H]+ | 412.3705 | 1 | 4α-Methylfecosterol | C29H48O | DW |
| 6.96_413.3784 | 6.96 | 413.3784 | [M+H]+ | 412.3705 | 1 | δ;7-Avenasterol | C29H48O | DW |
| 6.96_413.3784 | 6.96 | 413.3784 | [M+H]+ | 412.3705 | 1 | 4α-Methyl-23-methylene-cholest-5-en-3β-ol | C29H48O | DW |
| 6.96_413.3784 | 6.96 | 413.3784 | [M+H]+ | 412.3705 | 1 | Avenasterol | C29H48O | DW |
| 6.96_413.3784 | 6.96 | 413.3784 | [M+H]+ | 412.3705 | 1 | 24-Methylene lophenol | C29H48O | DW |
| 6.96_413.3784 | 6.96 | 413.3784 | [M+H]+ | 412.3705 | 1 | δ;7-Avenasterol | C29H48O | DW |
| 6.96_413.3784 | 6.96 | 413.3784 | [M+Li]+ | 406.3600 | 5 | 4,4\'-Diapophytofluene | C30H46 | DW |
| 2.21_417.2756 | 2.21 | 417.2756 | [M+Na]+ | 394.2872 | 1 | 25-hydroxy-16,17,23,23,24,24-hexadehydrovitamin D3 / 25-hydroxy-16,17,23,23,24,24-hexadehydrocholecalciferol | C27H38O2 | DW |
| 2.21_417.2756 | 2.21 | 417.2756 | [M+H]+ | 416.2692 | 2 | Oleoyl 3-carbacyclic Phosphatidic Acid | C22H41O5P | DW |
| 2.21_417.2756 | 2.21 | 417.2756 | [M+K]+ | 378.3134 | 2 | C-9,11,21-Trisnor-17-methyl-1α;,25-dihydroxyvitamin D3 | C24H42O3 | DW |
| 2.21_417.2756 | 2.21 | 417.2756 | [M+K]+ | 378.3134 | 2 | 5β;-Cholane-3α;,6α;,24-triol | C24H42O3 | DW |
| 2.21_417.2756 | 2.21 | 417.2756 | [M+K]+ | 378.3134 | 2 | 5β;-Cholane-3α;,7α;,12α;-triol | C24H42O3 | DW |
| 2.21_417.2756 | 2.21 | 417.2756 | [M+K]+ | 378.3134 | 2 | 5β;-Cholane-3α;,7α;,24-triol | C24H42O3 | DW |
| 2.21_417.2756 | 2.21 | 417.2756 | [M+K]+ | 378.3134 | 2 | 5β;-Cholane-3α;,7β;,24-triol | C24H42O3 | DW |
| 2.21_417.2756 | 2.21 | 417.2756 | [M+K]+ | 378.3134 | 2 | 5β;-Cholane-3α;,12α;,24-triol | C24H42O3 | DW |
| 2.21_417.2756 | 2.21 | 417.2756 | [M+2Na-H]+ | 372.3028 | 3 | (+)-Dysideapalaunic acid | C25H40O2 | DW |
| 2.21_417.2756 | 2.21 | 417.2756 | [M+Li]+ | 410.2569 | 6 | Polycylic aromatic hydrocarbon derivative (structurally similar to tilorone) | C25H34N2O3 | DW |
| 2.21_417.2756 | 2.21 | 417.2756 | [M+CH3OH+H]+ | 384.2447 | 6 | Organosulfur compound derivative (structurally similar to ibutilide) | C20H36N2O3S | DW |
| 6.28_427.3589 | 6.28 | 427.3589 | [M+H-2H2O]+ | 462.3709 | 1 | 2α;-(3-Hydroxypropyl)-1α;,25-dihydroxy-19-norvitamin D3 | C29H50O4 | DW |
| 6.28_427.3589 | 6.28 | 427.3589 | [M+H-H2O]+ | 444.3603 | 3 | 11α;-ethyl-1α;,25-dihydroxyvitamin D3 / 11α;-ethyl-1α;,25-dihydroxycholecalciferol | C29H48O3 | DW |
| 6.28_427.3589 | 6.28 | 427.3589 | [M+H-H2O]+ | 444.3603 | 3 | 1α;,25-dihydroxy-26,27-dimethylvitamin D3 / 1α;,25-dihydroxy-26,27-dimethylcholecalciferol | C29H48O3 | DW |
| 6.28_427.3589 | 6.28 | 427.3589 | [M+H-H2O]+ | 444.3603 | 3 | 1α;,25-dihydroxy-24α,24β-dihomovitamin D3 / 1α;,25-dihydroxy-24α,24β-dihomocholecalciferol | C29H48O3 | DW |
| 6.28_427.3589 | 6.28 | 427.3589 | [M+H-H2O]+ | 444.3603 | 3 | 1α;,25-dihydroxy-24α,24β-dihomo-20-epivitamin D3 / 1α;,25-dihydroxy-24α,24β-dihomo-20-epicholecalciferol | C29H48O3 | DW |
| 6.28_427.3589 | 6.28 | 427.3589 | [M+H-H2O]+ | 444.3603 | 3 | 3β-hydroxy-4β-methyl-5α-cholest-7-ene-4α-carboxylic acid | C29H48O3 | DW |
| 6.28_427.3589 | 6.28 | 427.3589 | [M+H-H2O]+ | 444.3603 | 3 | 3β-hydroxy-4α-methyl-5α-cholest-7-ene-4β-carboxylic acid | C29H48O3 | DW |
| 6.28_427.3589 | 6.28 | 427.3589 | [M+H-H2O]+ | 444.3603 | 3 | 4α-carboxy-4β-methyl-5α-cholesta-8-en-3β-ol | C29H48O3 | DW |
| 6.28_427.3589 | 6.28 | 427.3589 | [M+H]+ | 426.3498 | 4 | 1α;-hydroxy-24-methylvitamin D2 / 1α;-hydroxy-24-methylergocalciferol | C29H46O2 | DW |
| 6.28_427.3589 | 6.28 | 427.3589 | [M+H]+ | 426.3498 | 4 | zymosterol intermediate 1c | C29H46O2 | DW |
| 6.28_427.3589 | 6.28 | 427.3589 | [M+H]+ | 426.3498 | 4 | 4α-formyl-4β-methyl-5α-cholesta-8,24-dien-3β-ol | C29H46O2 | DW |
| 6.28_427.3589 | 6.28 | 427.3589 | [M+CH3OH+H]+ | 394.3236 | 4 | Ergosta-5,7,22,24(28)-tetraen-3β;-ol | C28H42O | DW |
| 6.28_427.3589 | 6.28 | 427.3589 | [M+CH3OH+H]+ | 394.3236 | 4 | Ergosta-5,7,24(28)-trienol | C28H42O | DW |
| 6.28_427.3589 | 6.28 | 427.3589 | [M+H]+ | 426.3484 | 7 | 3-Deoxy-3-azido-25-hydroxyvitamin D3 | C27H44N3O | DW |
| 6.28_427.3589 | 6.28 | 427.3589 | [M+Na]+ | 404.3654 | 9 | 5β;-Cholestane-3α;,7α;-diol | C27H48O2 | DW |
| 6.28_427.3589 | 6.28 | 427.3589 | [M+Na]+ | 404.3654 | 9 | 5β;-Cholestane-3α;,12α;-diol | C27H48O2 | DW |
| 6.28_427.3589 | 6.28 | 427.3589 | [M+Na]+ | 404.3654 | 9 | 5β;-Cholestane-3α;,26-diol | C27H48O2 | DW |
| 6.28_427.3589 | 6.28 | 427.3589 | [M+Na]+ | 404.3654 | 9 | 6α-hydroxycholestanol | C27H48O2 | DW |
| 3.75_494.3259 | 3.75 | 494.3259 | [M+CH3OH+H]+ | 461.2930 | 1 | 7-amino-4-methyl-coumarin (AMC) Arachidonoyl Amide | C30H39NO3 | DW |
| 3.75_494.3259 | 3.75 | 494.3259 | [M+H-H2O]+ | 511.3298 | 2 | Sphingolipid derivative (structurally similar to Scyphostatin A) | C31H45NO5 | DW |
| 3.75_494.3259 | 3.75 | 494.3259 | [M+H]+ | 493.3168 | 3 | PC(16:1(9Z)/0:0) | C24H48NO7P | DW |
| 3.75_494.3259 | 3.75 | 494.3259 | [M+H]+ | 493.3168 | 3 | LysoPC(16:1(9Z)) | C24H48NO7P | DW |
| 3.75_494.3259 | 3.75 | 494.3259 | [M+H]+ | 493.3168 | 3 | PC(16:1(9E)/0:0) | C24H48NO7P | DW |
| 3.75_494.3259 | 3.75 | 494.3259 | [M+Na]+ | 471.3349 | 3 | Cervonyl carnitine | C29H45NO4 | DW |
| 3.75_494.3259 | 3.75 | 494.3259 | [M+H]+ | 493.3226 | 7 | Diterpenoid derivative (structurally similar to tiamulin) | C28H47NO4S | DW |
| 3.75_494.3259 | 3.75 | 494.3259 | [M+Li]+ | 487.3062 | 7 | 2-(8-[3]-ladderane-octanyl)-sn-glycero-3-phosphoethanolamine | C25H46NO6P | DW |
| 3.59_518.3227 | 3.59 | 518.3227 | [M+Na]+ | 495.3325 | 1 | PC(O-14:0/2:0) | C24H50NO7P | DW |
| 3.59_518.3227 | 3.59 | 518.3227 | [M+Na]+ | 495.3325 | 1 | PC(16:0/0:0) | C24H50NO7P | DW |
| 3.59_518.3227 | 3.59 | 518.3227 | [M+Na]+ | 495.3325 | 1 | PC(0:0/16:0) | C24H50NO7P | DW |
| 3.59_518.3227 | 3.59 | 518.3227 | [M+Na]+ | 495.3325 | 1 | LysoPC(16:0) | C24H50NO7P | DW |
| 3.59_518.3227 | 3.59 | 518.3227 | [M+Na]+ | 495.3325 | 1 | PC(16:0/0:0)[U] / PC(16:0/0:0)[rac] | C24H50NO7P | DW |
| 3.59_518.3227 | 3.59 | 518.3227 | [M+2Na-H]+ | 473.3505 | 1 | Docosa-4,7,10,13,16-pentaenoyl carnitine | C29H47NO4 | DW |
| 3.59_518.3227 | 3.59 | 518.3227 | [M+2Na-H]+ | 473.3505 | 1 | Clupanodonyl carnitine | C29H47NO4 | DW |
| 3.59_518.3227 | 3.59 | 518.3227 | [M+H]+ | 517.3168 | 2 | PC(18:3(9Z,12Z,15Z)/0:0) | C26H48NO7P | DW |
| 3.59_518.3227 | 3.59 | 518.3227 | [M+H]+ | 517.3168 | 2 | LysoPC(18:3(6Z,9Z,12Z)) | C26H48NO7P | DW |
| 3.59_518.3227 | 3.59 | 518.3227 | [M+H]+ | 517.3168 | 2 | LysoPC(18:3(9Z,12Z,15Z)) | C26H48NO7P | DW |
| 3.59_518.3227 | 3.59 | 518.3227 | [M+H]+ | 517.3168 | 2 | PC(18:3(9Z,12Z,15Z)/0:0)[U] | C26H48NO7P | DW |
| 3.59_518.3227 | 3.59 | 518.3227 | [M+H]+ | 517.3168 | 2 | PC(18:3(6Z,9Z,12Z)/0:0)[U] | C26H48NO7P | DW |
| 3.59_518.3227 | 3.59 | 518.3227 | [M+H-H2O]+ | 535.3274 | 3 | PC(16:1(9Z)/2:0) | C26H50NO8P | DW |
| 3.59_518.3227 | 3.59 | 518.3227 | [M+2Na-H]+ | 473.3539 | 4 | (Z)-2-tetracos-15-enamidoethanesulfonic acid | C26H51NO4S | DW |
| 1.66_533.1525 | 1.66 | 533.1525 | [M+2Na-H]+ | 488.1835 | 4 | Sericetin diacetate | C29H28O7 | DW |
| 7.41_595.5318 | 7.41 | 595.5318 | [M+H]+ | 594.5223 | 3 | DG(18:1(11E)/16:0/0:0) | C37H70O5 | BW |
| 7.41_595.5318 | 7.41 | 595.5318 | [M+H]+ | 594.5223 | 3 | DG(18:1(9Z)/16:0/0:0) | C37H70O5 | BW |
| 7.41_595.5318 | 7.41 | 595.5318 | [M+H]+ | 594.5223 | 3 | DG(16:0/18:1(9Z)/0:0) | C37H70O5 | BW |
| 7.41_595.5318 | 7.41 | 595.5318 | [M+H]+ | 594.5223 | 3 | DG(17:0/17:1(9Z)/0:0)[iso2] | C37H70O5 | BW |
| 7.41_595.5318 | 7.41 | 595.5318 | [M+H]+ | 594.5223 | 3 | DG(16:1(9Z)/18:0/0:0)[iso2] | C37H70O5 | BW |
| 7.41_595.5318 | 7.41 | 595.5318 | [M+H]+ | 594.5223 | 3 | DG(16:0/18:1(11Z)/0:0) | C37H70O5 | BW |
| 7.41_595.5318 | 7.41 | 595.5318 | [M+H]+ | 594.5223 | 3 | DG(14:0/20:1(11Z)/0:0) | C37H70O5 | BW |
| 7.41_595.5318 | 7.41 | 595.5318 | [M+H]+ | 594.5223 | 3 | DG(14:1(9Z)/20:0/0:0) | C37H70O5 | BW |
| 7.41_595.5318 | 7.41 | 595.5318 | [M+H]+ | 594.5223 | 3 | DG(18:0/16:1(9Z)/0:0) | C37H70O5 | BW |
| 7.41_595.5318 | 7.41 | 595.5318 | [M+H]+ | 594.5223 | 3 | DG(18:1(11Z)/16:0/0:0) | C37H70O5 | BW |
| 7.41_595.5318 | 7.41 | 595.5318 | [M+H]+ | 594.5223 | 3 | DG(20:0/14:1(9Z)/0:0) | C37H70O5 | BW |
| 7.41_595.5318 | 7.41 | 595.5318 | [M+H]+ | 594.5223 | 3 | DG(20:1(11Z)/14:0/0:0) | C37H70O5 | BW |
| 7.07_617.5147 | 7.07 | 617.5147 | [M+H]+ | 616.5067 | 1 | DG(18:2(9Z,12Z)/18:2(9Z,12Z)/0:0) | C39H68O5 | BW |
| 7.07_617.5147 | 7.07 | 617.5147 | [M+H]+ | 616.5067 | 1 | DG(18:1(9Z)/18:3(9Z,12Z,15Z)/0:0)[iso2] | C39H68O5 | BW |
| 7.07_617.5147 | 7.07 | 617.5147 | [M+H]+ | 616.5067 | 1 | DG(16:1(9Z)/20:3(8Z,11Z,14Z)/0:0)[iso2] | C39H68O5 | BW |
| 7.07_617.5147 | 7.07 | 617.5147 | [M+H]+ | 616.5067 | 1 | DG(16:0/20:4(5Z,8Z,11Z,14Z)/0:0)[iso2] | C39H68O5 | BW |
| 7.07_617.5147 | 7.07 | 617.5147 | [M+H]+ | 616.5067 | 1 | DG(14:0/22:4(7Z,10Z,13Z,16Z)/0:0) | C39H68O5 | BW |
| 7.07_617.5147 | 7.07 | 617.5147 | [M+H]+ | 616.5067 | 1 | DG(16:0/20:4(8Z,11Z,14Z,17Z)/0:0) | C39H68O5 | BW |
| 7.07_617.5147 | 7.07 | 617.5147 | [M+H]+ | 616.5067 | 1 | DG(16:1(9Z)/20:3(5Z,8Z,11Z)/0:0) | C39H68O5 | BW |
| 7.07_617.5147 | 7.07 | 617.5147 | [M+H]+ | 616.5067 | 1 | DG(18:0/18:4(6Z,9Z,12Z,15Z)/0:0) | C39H68O5 | BW |
| 7.07_617.5147 | 7.07 | 617.5147 | [M+H]+ | 616.5067 | 1 | DG(18:1(11Z)/18:3(6Z,9Z,12Z)/0:0) | C39H68O5 | BW |
| 7.07_617.5147 | 7.07 | 617.5147 | [M+H]+ | 616.5067 | 1 | DG(18:1(11Z)/18:3(9Z,12Z,15Z)/0:0) | C39H68O5 | BW |
| 7.07_617.5147 | 7.07 | 617.5147 | [M+H]+ | 616.5067 | 1 | DG(18:1(9Z)/18:3(6Z,9Z,12Z)/0:0) | C39H68O5 | BW |
| 7.07_617.5147 | 7.07 | 617.5147 | [M+H]+ | 616.5067 | 1 | DG(18:3(6Z,9Z,12Z)/18:1(11Z)/0:0) | C39H68O5 | BW |
| 7.07_617.5147 | 7.07 | 617.5147 | [M+H]+ | 616.5067 | 1 | DG(18:3(6Z,9Z,12Z)/18:1(9Z)/0:0) | C39H68O5 | BW |
| 7.07_617.5147 | 7.07 | 617.5147 | [M+H]+ | 616.5067 | 1 | DG(18:3(9Z,12Z,15Z)/18:1(11Z)/0:0) | C39H68O5 | BW |
| 7.07_617.5147 | 7.07 | 617.5147 | [M+H]+ | 616.5067 | 1 | DG(18:3(9Z,12Z,15Z)/18:1(9Z)/0:0) | C39H68O5 | BW |
| 7.07_617.5147 | 7.07 | 617.5147 | [M+H]+ | 616.5067 | 1 | DG(18:4(6Z,9Z,12Z,15Z)/18:0/0:0) | C39H68O5 | BW |
| 7.07_617.5147 | 7.07 | 617.5147 | [M+H]+ | 616.5067 | 1 | DG(20:3(5Z,8Z,11Z)/16:1(9Z)/0:0) | C39H68O5 | BW |
| 7.07_617.5147 | 7.07 | 617.5147 | [M+H]+ | 616.5067 | 1 | DG(20:3(8Z,11Z,14Z)/16:1(9Z)/0:0) | C39H68O5 | BW |
| 7.07_617.5147 | 7.07 | 617.5147 | [M+H]+ | 616.5067 | 1 | DG(20:4(5Z,8Z,11Z,14Z)/16:0/0:0) | C39H68O5 | BW |
| 7.07_617.5147 | 7.07 | 617.5147 | [M+H]+ | 616.5067 | 1 | DG(20:4(8Z,11Z,14Z,17Z)/16:0/0:0) | C39H68O5 | BW |
| 7.07_617.5147 | 7.07 | 617.5147 | [M+H]+ | 616.5067 | 1 | DG(22:4(7Z,10Z,13Z,16Z)/14:0/0:0) | C39H68O5 | BW |
| 7.07_617.5147 | 7.07 | 617.5147 | [M+Na]+ | 594.5223 | 5 | DG(18:1(11E)/16:0/0:0) | C37H70O5 | BW |
| 7.07_617.5147 | 7.07 | 617.5147 | [M+Na]+ | 594.5223 | 5 | DG(18:1(9Z)/16:0/0:0) | C37H70O5 | BW |
| 7.07_617.5147 | 7.07 | 617.5147 | [M+Na]+ | 594.5223 | 5 | DG(16:0/18:1(9Z)/0:0) | C37H70O5 | BW |
| 7.07_617.5147 | 7.07 | 617.5147 | [M+Na]+ | 594.5223 | 5 | DG(17:0/17:1(9Z)/0:0)[iso2] | C37H70O5 | BW |
| 7.07_617.5147 | 7.07 | 617.5147 | [M+Na]+ | 594.5223 | 5 | DG(16:1(9Z)/18:0/0:0)[iso2] | C37H70O5 | BW |
| 7.07_617.5147 | 7.07 | 617.5147 | [M+Na]+ | 594.5223 | 5 | DG(16:0/18:1(11Z)/0:0) | C37H70O5 | BW |
| 7.07_617.5147 | 7.07 | 617.5147 | [M+Na]+ | 594.5223 | 5 | DG(14:0/20:1(11Z)/0:0) | C37H70O5 | BW |
| 7.07_617.5147 | 7.07 | 617.5147 | [M+Na]+ | 594.5223 | 5 | DG(14:1(9Z)/20:0/0:0) | C37H70O5 | BW |
| 7.07_617.5147 | 7.07 | 617.5147 | [M+Na]+ | 594.5223 | 5 | DG(18:0/16:1(9Z)/0:0) | C37H70O5 | BW |
| 7.07_617.5147 | 7.07 | 617.5147 | [M+Na]+ | 594.5223 | 5 | DG(18:1(11Z)/16:0/0:0) | C37H70O5 | BW |
| 7.07_617.5147 | 7.07 | 617.5147 | [M+Na]+ | 594.5223 | 5 | DG(20:0/14:1(9Z)/0:0) | C37H70O5 | BW |
| 7.07_617.5147 | 7.07 | 617.5147 | [M+Na]+ | 594.5223 | 5 | DG(20:1(11Z)/14:0/0:0) | C37H70O5 | BW |
| 8.5_719.4624 | 8.50 | 719.4624 | [M+Na]+ | 696.4730 | 0 | PA(18:2(9Z,12Z)/18:2(9Z,12Z))[U] | C39H69O8P | BW |
| 8.5_719.4624 | 8.50 | 719.4624 | [M+Na]+ | 696.4730 | 0 | PA(16:0/20:4(5Z,8Z,11Z,14Z)) | C39H69O8P | BW |
| 8.5_719.4624 | 8.50 | 719.4624 | [M+2Na-H]+ | 674.4886 | 3 | PA(16:0/18:1(11Z)) | C37H71O8P | BW |
| 8.5_719.4624 | 8.50 | 719.4624 | [M+2Na-H]+ | 674.4886 | 3 | PA(16:0/18:1(9Z))[U] | C37H71O8P | BW |
| 8.5_719.4624 | 8.50 | 719.4624 | [M+2Na-H]+ | 674.4886 | 3 | PA(18:1(9Z)/16:0)[U] | C37H71O8P | BW |
| 8.5_719.4624 | 8.50 | 719.4624 | [M+2Na-H]+ | 674.4886 | 3 | PA(16:0/18:1(9Z)) | C37H71O8P | BW |
| 8.5_741.4486 | 8.50 | 741.4486 | [M+2Na-H]+ | 696.4730 | 6 | PA(18:2(9Z,12Z)/18:2(9Z,12Z))[U] | C39H69O8P | BW |
| 8.5_741.4486 | 8.50 | 741.4486 | [M+2Na-H]+ | 696.4730 | 6 | PA(16:0/20:4(5Z,8Z,11Z,14Z)) | C39H69O8P | BW |
| 7.07_782.536 | 7.07 | 782.536 | [M+CH3OH+H]+ | 749.4996 | 3 | PC(12:0/22:6(4Z,7Z,10Z,13Z,16Z,19Z)) | C42H72NO8P | BW |
| 7.07_782.536 | 7.07 | 782.536 | [M+CH3OH+H]+ | 749.4996 | 3 | PC(14:1(9Z)/20:5(5Z,8Z,11Z,14Z,17Z)) | C42H72NO8P | BW |
| 7.07_782.536 | 7.07 | 782.536 | [M+CH3OH+H]+ | 749.4996 | 3 | PC(20:5(5Z,8Z,11Z,14Z,17Z)/14:1(9Z)) | C42H72NO8P | BW |
| 7.07_782.536 | 7.07 | 782.536 | [M+CH3OH+H]+ | 749.4996 | 3 | PE(15:0/22:6(4Z,7Z,10Z,13Z,16Z,19Z)) | C42H72NO8P | BW |
| 7.07_782.536 | 7.07 | 782.536 | [M+CH3OH+H]+ | 749.4996 | 3 | PE(22:6(4Z,7Z,10Z,13Z,16Z,19Z)/15:0) | C42H72NO8P | BW |
| 7.07_782.536 | 7.07 | 782.536 | [M+Li]+ | 775.5152 | 6 | PC(14:1(9Z)/22:6(4Z,7Z,10Z,13Z,16Z,19Z)) | C44H74NO8P | BW |
| 7.07_782.536 | 7.07 | 782.536 | [M+Li]+ | 775.5152 | 6 | PC(18:3(6Z,9Z,12Z)/18:4(6Z,9Z,12Z,15Z)) | C44H74NO8P | BW |
| 7.07_782.536 | 7.07 | 782.536 | [M+Li]+ | 775.5152 | 6 | PC(18:3(9Z,12Z,15Z)/18:4(6Z,9Z,12Z,15Z)) | C44H74NO8P | BW |
| 7.07_782.536 | 7.07 | 782.536 | [M+Li]+ | 775.5152 | 6 | PC(18:4(6Z,9Z,12Z,15Z)/18:3(6Z,9Z,12Z)) | C44H74NO8P | BW |
| 7.07_782.536 | 7.07 | 782.536 | [M+Li]+ | 775.5152 | 6 | PC(18:4(6Z,9Z,12Z,15Z)/18:3(9Z,12Z,15Z)) | C44H74NO8P | BW |
| 7.07_782.536 | 7.07 | 782.536 | [M+Li]+ | 775.5152 | 6 | PC(22:6(4Z,7Z,10Z,13Z,16Z,19Z)/14:1(9Z)) | C44H74NO8P | BW |
| 7.42_803.5648 | 7.42 | 803.5648 | [M+H]+ | 802.5598 | 2 | PG(17:0/20:4(5Z,8Z,11Z,14Z)) | C43H81NO10P | BW |
| 7.42_803.5648 | 7.42 | 803.5648 | [M+Na]+ | 780.5693 | 7 | Siphonaxanthin ester/ Siphonaxanthin dodecenoate/ (Siphonein) | C52H76O5 | BW |
| 7.4_936.6635 | 7.40 | 936.6635 | NC |  |  |  |  | BW |
| 7.42_937.6763 | 7.42 | 937.6763 | [M+K]+ | 898.7050 | 8 | TG(18:3(9Z,12Z,15Z)/18:3(9Z,12Z,15Z)/20:4(5Z,8Z,11Z,14Z))[iso3] | C59H94O6 | BW |
| 7.42_937.6763 | 7.42 | 937.6763 | [M+K]+ | 898.7050 | 8 | TG(16:0/20:5(5Z,8Z,11Z,14Z,17Z)/20:5(5Z,8Z,11Z,14Z,17Z))[iso3] | C59H94O6 | BW |
| 7.42_937.6763 | 7.42 | 937.6763 | [M+K]+ | 898.7050 | 8 | TG(16:1(9Z)/20:4(5Z,8Z,11Z,14Z)/20:5(5Z,8Z,11Z,14Z,17Z))[iso6] | C59H94O6 | BW |
| 7.42_937.6763 | 7.42 | 937.6763 | [M+K]+ | 898.7050 | 8 | TG(18:2(9Z,12Z)/18:3(9Z,12Z,15Z)/20:5(5Z,8Z,11Z,14Z,17Z))[iso6] | C59H94O6 | BW |
| 7.42_937.6763 | 7.42 | 937.6763 | [M+K]+ | 898.7050 | 8 | TG(17:2(9Z,12Z)/17:2(9Z,12Z)/22:6(4Z,7Z,10Z,13Z,16Z,19Z))[iso3] | C59H94O6 | BW |
| 7.42_937.6763 | 7.42 | 937.6763 | [M+K]+ | 898.7050 | 8 | TG(16:1(9Z)/18:3(9Z,12Z,15Z)/22:6(4Z,7Z,10Z,13Z,16Z,19Z))[iso6] | C59H94O6 | BW |
| 7.42_937.6763 | 7.42 | 937.6763 | [M+K]+ | 898.7050 | 8 | TG(18:3(9Z,12Z,15Z)/18:2(9Z,12Z)/20:5(5Z,8Z,11Z,14Z,17Z))[iso6] | C59H94O6 | BW |
| 7.42_937.6763 | 7.42 | 937.6763 | [M+K]+ | 898.7050 | 8 | TG(18:3(9Z,12Z,15Z)/20:4(5Z,8Z,11Z,14Z)/18:3(9Z,12Z,15Z))[iso3] | C59H94O6 | BW |
| 7.4_941.6199 | 7.40 | 941.6199 | NC |  |  |  |  | BW |
| 7.08_958.6475 | 7.08 | 958.6475 | NC |  |  |  |  | BW |
| 7.09_963.6014 | 7.09 | 963.6014 | NC |  |  |  |  | BW |
| 3.94_1039.664 | 3.94 | 1039.664 | [M+2Na-H]+ | 994.6944 | 1 | Dodecaprenyl diphosphate | C60H100O7P2 | DW |
