# Supplementary material for: Metabolite Profiling of a Diverse Collection of Wheat Lines Using Ultraperformance Liquid Chromatography Coupled with Time-of-Flight Mass Spectrometry
Source: PLoS One. 2012 Aug 30;7(8):e44179. doi: 10.1371/journal.pone.0044179 (PMC3431305; doi:10.1371/journal.pone.0044179)
Supplement: Table S3 — Best-choice tentative identities of discriminatory ions in the hexaploid HBW vs. SBW OPLS-DA model. The METLIN: Metabolite and Tandem MS Database was used to assign tentative compound identities and empirical formulas to the 31 influential ions with maximal m/z error set at 10 ppm under positive ionization adduct scan modes. Table columns: Ion Identifier (used in Figure S3, Panel A); Ion RT = ion retention time in minutes; Ion m/z = ion mass-to-charge ratio in daltons; Adduct = positive ionization adduct; Adduct Mass = mass of ion + adduct; Dppm = change (Δ, or d) in ppm from Ion m/z; Tentative Identity = identifier from METLIN; Empirical Formula = derived from Adduct Mass and Dppm; Class = class assignation according to the Lipid Maps Lipidomics Gateway as: 1) fatty acyls (FA), 2) glycerolipids (GL), 3) glycerophospholipids (GP), 4) sphingolipids (SP), 5) sterol lipids (ST), 6) prenol lipids (PR), 7) saccharolipids (SL), 8) polyketides (PK), additional classes ON (organonitrogen) and OS (organosulfur compounds), with NC classification if tentative identities could not be matched to the ion m/z; Overexpressed in: = ion spectral intensity higher in hard bread wheat (HBW) or soft bread wheat (SBW) as indicated. (DOCX) [file pone.0044179.s006.docx]

**Table S3. Best-choice tentative identities of discriminatory ions in the hexaploid HBW vs. SBW OPLS-DA model.**

| **Ion Identifier** | **Ion RT** | **Ion m/z** | **Adduct** | **Adduct Mass** | **Dppm** | **Tentative Identity** | **Empirical Formula** | **Class** | **Overexpressed In:** | **Broad Class** |
| --- | --- | --- | --- | --- | --- | --- | --- | --- | --- | --- |
| 6.70_647.4886 | 6.70 | 647.4886 | NC |  |  |  |  |  | HBW |  |
| 6.14_691.5134 | 6.14 | 691.5134 | NC |  |  |  |  |  | HBW |  |
| 6.99_573.4888 | 6.99 | 573.4888 | [M+H-H2O]+ | 590.4910 | 0 | 13 DG derivatives | C37H66O5 | GL | HBW | PL |
| 7.01_591.4992 | 7.01 | 591.4992 | [M+H]+ | 590.4910 | 1 | 13 DG derivatives | C37H66O5 | GL | HBW | PL |
| 6.05_665.4967 | 6.05 | 665.4967 | [M+H-2H2O]+ | 700.5043 | 7 | 7 PA derivatives | C39H73O8P | GL | HBW | PL |
| 6.81_655.4929 | 6.81 | 655.4929 | [M+Li]+ | 648.4730 | 5 | PA(16:0/16:0)[rac] | C35H69O8P | GP | HBW | PL |
| 5.81_689.4986 | 5.81 | 689.4986 | [M+H-2H2O]+ | 724.5129 | 2 | PG(17:0/14:1(9Z)) | C37H75NO10P | GP | HBW | PL |
| 6.40_756.5555 | 6.40 | 756.5555 | [M+H-2H2O]+ | 791.5676 | 0 | 12 PE derivatives, 1 PE N-methyl derivative, 26 PC derivatives | C42H82NO10P | GP | HBW | PL |
| 0.53_268.1037 | 0.53 | 268.1037 | [M+H]+ | 267.0968 | 1 | 5 nucleosides and derivatives | C10H13N5O4 | ON | HBW | PL |
| 4.20_496.3345 | 4.20 | 496.3345 | [M+CH3OH+H]+ | 463.3006 | 0 | Aminoglycoside (structurally similar to Gentamicin C2) | C20H41N5O7 | ON | HBW | PL |
| 3.95_520.3216 | 3.95 | 520.3216 | [M+Na]+ | 497.3353 | 5 | Tumonoic Acid I | C27H47NO7 | ON | HBW | PL |
| 3.94_1039.664 | 3.94 | 1039.6640 | [M+2Na-H]+ | 994.6944 | 1 | Dodecaprenyl diphosphate | C60H100O7P2 | PR | HBW | PL |
| 7.04_938.5852 | 7.04 | 938.5852 | NC |  |  |  |  |  | SBW |  |
| 6.83_962.5886 | 6.83 | 962.5886 | NC |  |  |  |  |  | SBW |  |
| 6.42_979.5973 | 6.42 | 979.5973 | NC |  |  |  |  |  | SBW |  |
| 6.21_375.3266 | 6.21 | 375.3266 | [M+H-2H2O]+ | 410.3396 | 0 | 2 MG derivatives | C25H46O4 | GL | SBW | PL |
| 6.90_738.5075 | 6.90 | 738.5075 | [M+H]+ | 737.4996 | 0 | 21 PE derivatives | C41H72NO8P | GP | SBW | PL |
| 7.15_740.5198 | 7.15 | 740.5198 | [M+Na]+ | 717.5308 | 0 | 21 PE derivatives | C39H76NO8P | GP | SBW | PL |
| 7.03_756.5511 | 7.03 | 756.5511 | [M+Na]+ | 733.5621 | 0 | PE(17:0/18:0)[U] | C40H80NO8P | GP | SBW | PL |
| 6.90_760.4936 | 6.90 | 760.4936 | [M+H]+ | 759.4839 | 3 | 8 PE derivatives | C43H70NO8P | GP | SBW | PL |
| 6.82_780.5501 | 6.82 | 780.5501 | [M+Na]+ | 757.5621 | 1 | 4 PE N-methyl derivatives, 2 PE derivatives, 37 PC derivatives | C42H80NO8P | GP | SBW | PL |
| 7.07_782.536 | 7.07 | 782.5360 | [M+CH3OH+H]+ | 749.4996 | 3 | 3 PC derivatives, 2 PE derivatives | C42H72NO8P | GP | SBW | PL |
| 1.71_144.0678 | 1.71 | 144.0678 | [M+H-2H2O]+ | 179.0807 | 1 | 7-Aminomethyl-7-carbaguanine | C7H9N5O | ON | SBW | PL |
| 0.58_279.0486 | 0.58 | 279.0486 | [M+CH3OH+H]+ | 246.0149 | 0 | Oxdemetonmethyl | C6H15O4PS2 | OS | SBW | PL |
| 0.58_279.0486 | 0.58 | 279.0486 | [M+H-2H2O]+ | 314.0613 | 0 | Organosulfur derivative (structurally similar to Rofecoxib) | C17H14O4S | OS | SBW | PL |
| 1.71_144.0678 | 1.71 | 144.0678 | [M+2H]2+ | 286.1205 | 1 | 24 flavonoids/ methylflavonoids and derivatives | C17H18O4 | PK | SBW | PL |
| 0.58_279.0486 | 0.58 | 279.0486 | [M+H+Na]2+ | 534.1010 | 0 | 12 flavonoid glucosides and derivatives | C24H22O14 | PK | SBW | PL |
| 0.49_381.0751 | 0.49 | 381.0751 | [M+Na]+ | 358.0841 | 4 | 5,7-Dihydroxyflavone 7-benzoate | C22H14O5 | PK | SBW | PL |
| 0.49_381.0751 | 0.49 | 381.0751 | [M+H-2H2O]+ | 416.0896 | 4 | Flavonoid derivative (structurally similar to Calomelanol D) | C24H16O7 | PK | SBW | PL |
| 0.49_381.0751 | 0.49 | 381.0751 | [M+K]+ | 342.1103 | 4 | 25 tetramethylflavonoids and derivatives | C19H18O6 | PK | SBW | PL |
| 0.58_441.1012 | 0.58 | 441.1012 | [M+H+Na]2+ | 858.2066 | 0 | 3 flavonoid glucosides and derivatives | C36H42O24 | PK | SBW | PL |
| 0.58_441.1012 | 0.58 | 441.1012 | [M+Li]+ | 434.0849 | 0 | 16 flavonoid glucosides and derivatives | C20H18O11 | PK | SBW | PL |
| 0.57_783.2171 | 0.57 | 783.2171 | [M+H-H2O]+ | 800.2164 | 4 | 5 flavonoid glucosides and derivatives | C38H40O19 | PK | SBW | PL |
| 6.83_961.5853 | 6.83 | 961.5853 | [M+H]+ | 960.5769 | 1 | Megalomicin C1 | C48H84N2O17 | PK | SBW | PL |
| 0.53_118.0751 | 0.53 | 118.0751 | [M+2Na]2+ | 190.1722 | 1 | Caryophyllene [t(-)] | C14H22 | PR | SBW | PL |
| 7.02_937.5846 | 7.02 | 937.5846 | [M+H]+ | 936.5810 | 3 | 1,2-Di-(9Z,12Z,15Z-octadecatrienoyl)-3-(Galactosyl-alpha-1-6-Galactosyl-beta-1)-glycerol | C51H84O15 | SL | SBW | PL |
| 5.84_463.3213 | 5.84 | 463.3213 | [M+Li]+ | 456.3051 | 0 | 24,24-Difluoro-1,25,26-trihydroxyvitamin D3 | C26H42F2O4 | ST | SBW | NP |
| 5.84_463.3213 | 5.84 | 463.3213 | [M+H-H2O]+ | 480.3239 | 0 | Vitamin D derivative (structurally similar to EB 1213) | C31H44O4 | ST | SBW | NP |
