# Supplementary material for: Metabolite Profiling of a Diverse Collection of Wheat Lines Using Ultraperformance Liquid Chromatography Coupled with Time-of-Flight Mass Spectrometry
Source: PLoS One. 2012 Aug 30;7(8):e44179. doi: 10.1371/journal.pone.0044179 (PMC3431305; doi:10.1371/journal.pone.0044179)
Supplement: Table S4 — Characteristics and all possible tentative identities for discriminatory ions in the hexaploid HBW vs. SBW OPLS-DA model. The METLIN: Metabolite and Tandem MS Database was used to assign tentative compound identities and empirical formulas to the 31 influential ions with maximal m/z error set at 10 ppm under positive ionization adduct scan modes. Table columns: Ion Identifier; Ion RT = ion retention time in minutes; Ion m/z = ion mass-to-charge ratio in daltons; Adduct = positive ionization adduct; Adduct Mass = mass of ion + adduct; Dppm = change (Δ, or d) in ppm from Ion m/z; Tentative Identity = identifier from METLIN; Empirical Formula = derived from Adduct Mass and Dppm. NC = not classified; no tentative compound identity hits within limits of METLIN search. (DOCX) [file pone.0044179.s007.docx]

**Table S4. Characteristics and all possible tentative identities for discriminatory ions in the hexaploid HBW vs. SBW OPLS-DA model.**

| **Ion Identifier** | **Ion RT** | **Ion m/z** | **Adduct** | **Adduct Mass** | **Dppm** | **Tentative Identity** | **Empirical Formula** | **Overexpressed In:** |
| --- | --- | --- | --- | --- | --- | --- | --- | --- |
| 0.53_118.0751 | 0.53 | 118.0751 | [M+2Na]2+ | 190.1722 | 1 | Caryophyllene [t(-)] | C14H22 | SBW |
| 0.53_118.0751 | 0.53 | 118.0751 | [M+3H]3+ | 351.2046 | 3 | Dipivefrin | C19H29NO5 | SBW |
| 0.53_118.0751 | 0.53 | 118.0751 | [M+2H]2+ | 234.1368 | 4 | 2H-Indol-2-one, 1,3-dihydro-7-hydroxy-4-[2-(propylamino)ethyl]- | C13H18N2O2 | SBW |
| 1.71_144.0678 | 1.71 | 144.0678 | [M+H-2H2O]+ | 179.0807 | 1 | 7-Aminomethyl-7-carbaguanine | C7H9N5O | SBW |
| 1.71_144.0678 | 1.71 | 144.0678 | [M+2H]2+ | 286.1205 | 1 | Phenylpropanoid derivative (structurally similar to CAY10616) | C17H18O4 | SBW |
| 1.71_144.0678 | 1.71 | 144.0678 | [M+2H]2+ | 286.1205 | 1 | Flavonoid derivative (structurally similar to Tupichinol A) | C17H18O4 | SBW |
| 1.71_144.0678 | 1.71 | 144.0678 | [M+2H]2+ | 286.1205 | 1 | 7,3\'-Dihydroxy-4\'-methoxy-8-methylflavan | C17H18O4 | SBW |
| 1.71_144.0678 | 1.71 | 144.0678 | [M+2H]2+ | 286.1205 | 1 | 7,4\'-Dihydroxy-3\'-methoxy-8-methylflavan | C17H18O4 | SBW |
| 1.71_144.0678 | 1.71 | 144.0678 | [M+2H]2+ | 286.1205 | 1 | 4\'-Hydroxy-7,3\'-dimethoxyflavan | C17H18O4 | SBW |
| 1.71_144.0678 | 1.71 | 144.0678 | [M+2H]2+ | 286.1205 | 1 | 7-Hydroxy-5,4\'-dimethoxyflavan | C17H18O4 | SBW |
| 1.71_144.0678 | 1.71 | 144.0678 | [M+2H]2+ | 286.1205 | 1 | 5,4\'-Dihydroxy-7\'-methoxy-8-methylflavan | C17H18O4 | SBW |
| 1.71_144.0678 | 1.71 | 144.0678 | [M+2H]2+ | 286.1205 | 1 | 4\'-Hydroxy-5,7-dimethoxyflavan | C17H18O4 | SBW |
| 1.71_144.0678 | 1.71 | 144.0678 | [M+2H]2+ | 286.1205 | 1 | (-)-Sativan | C17H18O4 | SBW |
| 1.71_144.0678 | 1.71 | 144.0678 | [M+2H]2+ | 286.1205 | 1 | Isosativan | C17H18O4 | SBW |
| 1.71_144.0678 | 1.71 | 144.0678 | [M+2H]2+ | 286.1205 | 1 | Arvensan | C17H18O4 | SBW |
| 1.71_144.0678 | 1.71 | 144.0678 | [M+2H]2+ | 286.1205 | 1 | Latifolin | C17H18O4 | SBW |
| 1.71_144.0678 | 1.71 | 144.0678 | [M+2H]2+ | 286.1205 | 1 | (S)-5,3\'-Dihydroxy-2,4-dimethoxydalbergiquinol | C17H18O4 | SBW |
| 1.71_144.0678 | 1.71 | 144.0678 | [M+2H]2+ | 286.1205 | 1 | (R)-2,5-Dihydroxy-3,4-dimethoxydalbergiquinol | C17H18O4 | SBW |
| 1.71_144.0678 | 1.71 | 144.0678 | [M+2H]2+ | 286.1205 | 1 | 4-Hydroxy-2\',4\'-dimethoxydihydrochalcone | C17H18O4 | SBW |
| 1.71_144.0678 | 1.71 | 144.0678 | [M+2H]2+ | 286.1205 | 1 | 4\'-Hydroxy-2,6-dimethoxydihydrochalcone | C17H18O4 | SBW |
| 1.71_144.0678 | 1.71 | 144.0678 | [M+2H]2+ | 286.1205 | 1 | Myrigalone H | C17H18O4 | SBW |
| 1.71_144.0678 | 1.71 | 144.0678 | [M+2H]2+ | 286.1205 | 1 | 2\',6\'-Dihydroxy-4\'-methoxy-3\'-methyldihydrochalcone | C17H18O4 | SBW |
| 1.71_144.0678 | 1.71 | 144.0678 | [M+2H]2+ | 286.1205 | 1 | Dihydroflavokawin B | C17H18O4 | SBW |
| 1.71_144.0678 | 1.71 | 144.0678 | [M+2H]2+ | 286.1205 | 1 | Flavonoid derivative (structurally similar to Loureirin A) | C17H18O4 | SBW |
| 1.71_144.0678 | 1.71 | 144.0678 | [M+2H]2+ | 286.1205 | 1 | 2-O-Methylangolensin | C17H18O4 | SBW |
| 1.71_144.0678 | 1.71 | 144.0678 | [M+2H]2+ | 286.1205 | 1 | 4-O-Methylangolensin | C17H18O4 | SBW |
| 1.71_144.0678 | 1.71 | 144.0678 | [M+2H]2+ | 286.1205 | 1 | Flavonoid derivative (structurally similar to Bifemelane (M5)) | C17H18O4 | SBW |
| 1.71_144.0678 | 1.71 | 144.0678 | [M+2H]2+ | 286.1205 | 1 | Flavonoid derivative (structurally similar to Bifemelane (M4)) | C17H18O4 | SBW |
| 1.71_144.0678 | 1.71 | 144.0678 | [M+H-2H2O]+ | 179.0794 | 8 | Fructosamine | C6H13NO5 | SBW |
| 1.71_144.0678 | 1.71 | 144.0678 | [M+H-2H2O]+ | 179.0794 | 8 | D-Galactosamine | C6H13NO5 | SBW |
| 1.71_144.0678 | 1.71 | 144.0678 | [M+H-2H2O]+ | 179.0794 | 8 | D-Glucosamine | C6H13NO5 | SBW |
| 1.71_144.0678 | 1.71 | 144.0678 | [M+H-2H2O]+ | 179.0794 | 8 | Amine monosaccharide (structurally similar to kanosamine) | C6H13NO5 | SBW |
| 1.71_144.0678 | 1.71 | 144.0678 | [M+H-2H2O]+ | 179.0794 | 8 | 1-Amino-1-deoxy-scyllo-inositol | C6H13NO5 | SBW |
| 1.71_144.0678 | 1.71 | 144.0678 | [M+H-2H2O]+ | 179.0794 | 8 | Glucosamine | C6H13NO5 | SBW |
| 0.53_268.1037 | 0.53 | 268.1037 | [M+H]+ | 267.0968 | 1 | Adenosine | C10H13N5O4 | HBW |
| 0.53_268.1037 | 0.53 | 268.1037 | [M+H]+ | 267.0968 | 1 | Purine nucleoside (structurally similar to vidarabine) | C10H13N5O4 | HBW |
| 0.53_268.1037 | 0.53 | 268.1037 | [M+H]+ | 267.0968 | 1 | Pyrimidine nucleoside (structurally similar to zidovudine) | C10H13N5O4 | HBW |
| 0.53_268.1037 | 0.53 | 268.1037 | [M+H]+ | 267.0968 | 1 | Deoxyguanosine | C10H13N5O4 | HBW |
| 0.53_268.1037 | 0.53 | 268.1037 | [M+H]+ | 267.0968 | 1 | 3-Deoxyguanosine | C10H13N5O4 | HBW |
| 0.53_268.1037 | 0.53 | 268.1037 | [M+H]+ | 267.0954 | 3 | Neuraminic acid | C9H17NO8 | HBW |
| 0.53_268.1037 | 0.53 | 268.1037 | [M+Na]+ | 245.1164 | 7 | Acetyltryptophanamide | C13H15N3O2 | HBW |
| 0.58_279.0486 | 0.58 | 279.0486 | [M+H-2H2O]+ | 314.0613 | 0 | Organosulfur derivative (structurally similar to rofecoxib) | C17H14O4S | SBW |
| 0.58_279.0486 | 0.58 | 279.0486 | [M+H+Na]2+ | 534.1010 | 0 | Cyanidin 3-(3\'\'-malonylglucoside) | C24H22O14 | SBW |
| 0.58_279.0486 | 0.58 | 279.0486 | [M+H+Na]2+ | 534.1010 | 0 | Cyanidin-3-O-(6-O-malonyl-beta-D-glucopyranoside) | C24H22O14 | SBW |
| 0.58_279.0486 | 0.58 | 279.0486 | [M+H+Na]2+ | 534.1010 | 0 | Orobol 7-O-(6\'\'-malonylglucoside) | C24H22O14 | SBW |
| 0.58_279.0486 | 0.58 | 279.0486 | [M+H+Na]2+ | 534.1010 | 0 | Luteolin 7-O-(6\'\'-malonylglucoside) | C24H22O14 | SBW |
| 0.58_279.0486 | 0.58 | 279.0486 | [M+H+Na]2+ | 534.1010 | 0 | Luteolin 5-(6\'\'-malonylglucoside) | C24H22O14 | SBW |
| 0.58_279.0486 | 0.58 | 279.0486 | [M+H+Na]2+ | 534.1010 | 0 | Luteolin 7-(2\'\'-glucuronosyllactate) | C24H22O14 | SBW |
| 0.58_279.0486 | 0.58 | 279.0486 | [M+H+Na]2+ | 534.1010 | 0 | Scutellarein 7-(6\'\'-malonylglucoside) | C24H22O14 | SBW |
| 0.58_279.0486 | 0.58 | 279.0486 | [M+H+Na]2+ | 534.1010 | 0 | Kaempferol 3-(6\'\'-malonylgalactoside) | C24H22O14 | SBW |
| 0.58_279.0486 | 0.58 | 279.0486 | [M+H+Na]2+ | 534.1010 | 0 | Kaempferol 3-(6\'\'-malonylglucoside) | C24H22O14 | SBW |
| 0.58_279.0486 | 0.58 | 279.0486 | [M+H+Na]2+ | 534.1010 | 0 | Quercetin 3-(4\'\'-malonylrhamnoside) | C24H22O14 | SBW |
| 0.58_279.0486 | 0.58 | 279.0486 | [M+H+Na]2+ | 534.1010 | 0 | 5,4\'-Dihydroxy-3,3\'-dimethoxy-6,7-methylenedioxyflavone 4\'-glucuronide | C24H22O14 | SBW |
| 0.58_279.0486 | 0.58 | 279.0486 | [M+H+Na]2+ | 534.1010 | 0 | Cyanidin 3-O-(6-O-malonyl-&beta;-D-glucoside) | C24H22O14 | SBW |
| 0.58_279.0486 | 0.58 | 279.0486 | [M+CH3OH+H]+ | 246.0149 | 0 | Organophosphorus derivative (structurally similar to oxdemetonmethyl) | C6H15O4PS2 | SBW |
| 0.58_279.0486 | 0.58 | 279.0486 | [M+H-H2O]+ | 296.0509 | 1 | Organosulfur derivative (structurally similar to disulfiram) | C10H20N2S4 | SBW |
| 0.58_279.0486 | 0.58 | 279.0486 | [M+Li]+ | 272.0321 | 1 | Quinone derivative (structurally similar to quinalizarin) | C14H8O6 | SBW |
| 0.58_279.0486 | 0.58 | 279.0486 | [M+Na]+ | 256.0583 | 3 | Piscidic acid | C11H12O7 | SBW |
| 0.58_279.0486 | 0.58 | 279.0486 | [M+H-2H2O]+ | 314.0589 | 8 | Organophosphorus derivative (structurally similar to malaoxon) | C10H19O7PS | SBW |
| 0.58_279.0486 | 0.58 | 279.0486 | [M+H-2H2O]+ | 314.0638 | 8 | Salicyl phenolic glucuronide | C13H14O9 | SBW |
| 0.58_279.0486 | 0.58 | 279.0486 | [M+H-2H2O]+ | 314.0638 | 8 | Salicyl acyl glucuronide | C13H14O9 | SBW |
| 6.21_375.3266 | 6.21 | 375.3266 | [M+H-2H2O]+ | 410.3396 | 0 | MG(0:0/22:2(13Z,16Z)/0:0) | C25H46O4 | SBW |
| 6.21_375.3266 | 6.21 | 375.3266 | [M+H-2H2O]+ | 410.3396 | 0 | MG(22:2(13Z,16Z)/0:0/0:0) | C25H46O4 | SBW |
| 6.21_375.3266 | 6.21 | 375.3266 | [M+H]+ | 374.3185 | 2 | 12,14-Pentacosadiynoic acid | C25H42O2 | SBW |
| 6.21_375.3266 | 6.21 | 375.3266 | [M+H]+ | 374.3185 | 2 | Dolichoic acid-[18-20] | C25H42O2 | SBW |
| 6.21_375.3266 | 6.21 | 375.3266 | [M+Na]+ | 352.3341 | 8 | 22-tricosenoic acid | C23H44O2 | SBW |
| 6.21_375.3266 | 6.21 | 375.3266 | [M+Na]+ | 352.3341 | 8 | 14E-tricosenoic acid | C23H44O2 | SBW |
| 6.21_375.3266 | 6.21 | 375.3266 | [M+Na]+ | 352.3341 | 8 | 14Z-tricosenoic acid | C23H44O2 | SBW |
| 6.21_375.3266 | 6.21 | 375.3266 | [M+Na]+ | 352.3341 | 8 | 16Z-tricosenoic acid | C23H44O2 | SBW |
| 6.21_375.3266 | 6.21 | 375.3266 | [M+Na]+ | 352.3341 | 8 | 17Z-tricosenoic acid | C23H44O2 | SBW |
| 6.21_375.3266 | 6.21 | 375.3266 | [M+Na]+ | 352.3341 | 8 | 18Z-tricosenoic acid | C23H44O2 | SBW |
| 0.49_381.0751 | 0.49 | 381.0751 | [M+Na]+ | 358.0841 | 4 | 5,7-Dihydroxyflavone 7-benzoate | C22H14O5 | SBW |
| 0.49_381.0751 | 0.49 | 381.0751 | [M+H-2H2O]+ | 416.0896 | 4 | Calomelanol D | C24H16O7 | SBW |
| 0.49_381.0751 | 0.49 | 381.0751 | [M+K]+ | 342.1103 | 4 | Mundoserone | C19H18O6 | SBW |
| 0.49_381.0751 | 0.49 | 381.0751 | [M+K]+ | 342.1103 | 4 | Isotectorigenin trimethyl ether | C19H18O6 | SBW |
| 0.49_381.0751 | 0.49 | 381.0751 | [M+K]+ | 342.1103 | 4 | 12α-hydroxy-5-deoxydehydomundoserone | C19H18O6 | SBW |
| 0.49_381.0751 | 0.49 | 381.0751 | [M+K]+ | 342.1103 | 4 | 7,2\',4\',5\'-Tetramethoxyisoflavone | C19H18O6 | SBW |
| 0.49_381.0751 | 0.49 | 381.0751 | [M+K]+ | 342.1103 | 4 | 6,7,3\',4\'-Tetramethoxyisoflavone | C19H18O6 | SBW |
| 0.49_381.0751 | 0.49 | 381.0751 | [M+K]+ | 342.1103 | 4 | Bryacarpene 3 | C19H18O6 | SBW |
| 0.49_381.0751 | 0.49 | 381.0751 | [M+K]+ | 342.1103 | 4 | 7,2\',4\',5\'-Tetramethoxyflavone | C19H18O6 | SBW |
| 0.49_381.0751 | 0.49 | 381.0751 | [M+K]+ | 342.1103 | 4 | Zapotin | C19H18O6 | SBW |
| 0.49_381.0751 | 0.49 | 381.0751 | [M+K]+ | 342.1103 | 4 | Cerrosillin | C19H18O6 | SBW |
| 0.49_381.0751 | 0.49 | 381.0751 | [M+K]+ | 342.1103 | 4 | 5,7,2\',5\'-tetramethoxyflavone | C19H18O6 | SBW |
| 0.49_381.0751 | 0.49 | 381.0751 | [M+K]+ | 342.1103 | 4 | 5,7-Dihydroxy-3\',4\'-dimethoxy-6,8-dimethylflavone | C19H18O6 | SBW |
| 0.49_381.0751 | 0.49 | 381.0751 | [M+K]+ | 342.1103 | 4 | Norartocarpetin 5,7,2\',4\'-tetramethyl ether | C19H18O6 | SBW |
| 0.49_381.0751 | 0.49 | 381.0751 | [M+K]+ | 342.1103 | 4 | Luteolin 5,7,3\',4\'-tetramethyl ether | C19H18O6 | SBW |
| 0.49_381.0751 | 0.49 | 381.0751 | [M+K]+ | 342.1103 | 4 | Scutellarein 5,6,7,4\'-tetramethyl ether | C19H18O6 | SBW |
| 0.49_381.0751 | 0.49 | 381.0751 | [M+K]+ | 342.1103 | 4 | 6-Demethoxytangeritin | C19H18O6 | SBW |
| 0.49_381.0751 | 0.49 | 381.0751 | [M+K]+ | 342.1103 | 4 | 5,6,7,8-Tetramethoxyflavone | C19H18O6 | SBW |
| 0.49_381.0751 | 0.49 | 381.0751 | [M+K]+ | 342.1103 | 4 | Fisetin tetramethyl ether | C19H18O6 | SBW |
| 0.49_381.0751 | 0.49 | 381.0751 | [M+K]+ | 342.1103 | 4 | 6,8-Di-C-methylkaempferol 3,7-dimethyl ether | C19H18O6 | SBW |
| 0.49_381.0751 | 0.49 | 381.0751 | [M+K]+ | 342.1103 | 4 | 8-Desmethylkalmiatin | C19H18O6 | SBW |
| 0.49_381.0751 | 0.49 | 381.0751 | [M+K]+ | 342.1103 | 4 | Tetramethylkaempferol | C19H18O6 | SBW |
| 0.49_381.0751 | 0.49 | 381.0751 | [M+K]+ | 342.1103 | 4 | 3,5,6,7-Tetramethoxyflavone | C19H18O6 | SBW |
| 0.49_381.0751 | 0.49 | 381.0751 | [M+K]+ | 342.1103 | 4 | 3,5,7,8-Tetramethoxyflavone | C19H18O6 | SBW |
| 0.49_381.0751 | 0.49 | 381.0751 | [M+K]+ | 342.1103 | 4 | 3\'-(2-Hydroxy-3-methylbut-3-enyl)-4,2\',4\'-trihydroxychalcone | C19H18O6 | SBW |
| 0.49_381.0751 | 0.49 | 381.0751 | [M+K]+ | 342.1103 | 4 | 3,4-Methylenedioxy-2\',4\',6\'-trimethoxychalcone | C19H18O6 | SBW |
| 0.49_381.0751 | 0.49 | 381.0751 | [M+K]+ | 342.1103 | 4 | Tinosporinone | C19H18O6 | SBW |
| 0.49_381.0751 | 0.49 | 381.0751 | [M+K]+ | 342.1103 | 4 | Rengasin trimethyl ether | C19H18O6 | SBW |
| 0.58_441.1012 | 0.58 | 441.1012 | [M+H+Na]2+ | 858.2066 | 0 | Cyanidin 3-(6\'\'-malonylsophoroside)-5-glucoside | C36H42O24 | SBW |
| 0.58_441.1012 | 0.58 | 441.1012 | [M+H+Na]2+ | 858.2066 | 0 | Cyanidin 3-(3-glucosyl-6-malonylglucoside)-4\'-glucoside | C36H42O24 | SBW |
| 0.58_441.1012 | 0.58 | 441.1012 | [M+H+Na]2+ | 858.2066 | 0 | Delphinidin 3-neohesperidoside-7-(6-malonylglucoside) | C36H42O24 | SBW |
| 0.58_441.1012 | 0.58 | 441.1012 | [M+Li]+ | 434.0849 | 0 | Tricetin 3\'-xyloside | C20H18O11 | SBW |
| 0.58_441.1012 | 0.58 | 441.1012 | [M+Li]+ | 434.0849 | 0 | Isoetin 2\'-xyloside | C20H18O11 | SBW |
| 0.58_441.1012 | 0.58 | 441.1012 | [M+Li]+ | 434.0849 | 0 | 6-Hydroxyluteolin 6-xyloside | C20H18O11 | SBW |
| 0.58_441.1012 | 0.58 | 441.1012 | [M+Li]+ | 434.0849 | 0 | 6-Hydroxyluteolin 7-arabinopyranoside | C20H18O11 | SBW |
| 0.58_441.1012 | 0.58 | 441.1012 | [M+Li]+ | 434.0849 | 0 | 6-Hydroxyluteolin 7-xyloside | C20H18O11 | SBW |
| 0.58_441.1012 | 0.58 | 441.1012 | [M+Li]+ | 434.0849 | 0 | 6-Hydroxyluteolin 7-apioside | C20H18O11 | SBW |
| 0.58_441.1012 | 0.58 | 441.1012 | [M+Li]+ | 434.0849 | 0 | 8-Hydroxyluteolin 7-xyloside | C20H18O11 | SBW |
| 0.58_441.1012 | 0.58 | 441.1012 | [M+Li]+ | 434.0849 | 0 | Avicularin | C20H18O11 | SBW |
| 0.58_441.1012 | 0.58 | 441.1012 | [M+Li]+ | 434.0849 | 0 | Guaijaverin | C20H18O11 | SBW |
| 0.58_441.1012 | 0.58 | 441.1012 | [M+Li]+ | 434.0849 | 0 | Quercetin 3-beta-L-arabinopyranoside | C20H18O11 | SBW |
| 0.58_441.1012 | 0.58 | 441.1012 | [M+Li]+ | 434.0849 | 0 | Quercetin 7-xyloside | C20H18O11 | SBW |
| 0.58_441.1012 | 0.58 | 441.1012 | [M+Li]+ | 434.0849 | 0 | Quercetin 3\'-xyloside | C20H18O11 | SBW |
| 0.58_441.1012 | 0.58 | 441.1012 | [M+Li]+ | 434.0849 | 0 | Quercetin 3-O-alpha-D-arabinopyranoside | C20H18O11 | SBW |
| 0.58_441.1012 | 0.58 | 441.1012 | [M+Li]+ | 434.0849 | 0 | Herbacetin 7-beta-L-arabinopyranoside | C20H18O11 | SBW |
| 0.58_441.1012 | 0.58 | 441.1012 | [M+Li]+ | 434.0849 | 0 | Herbacetin 8-alpha-L-arabinopyranoside | C20H18O11 | SBW |
| 0.58_441.1012 | 0.58 | 441.1012 | [M+Li]+ | 434.0849 | 0 | Herbacetin 8-xyloside | C20H18O11 | SBW |
| 5.84_463.3213 | 5.84 | 463.3213 | [M+H-H2O]+ | 480.3239 | 0 | Steroid derivative (structurally similar to EB 1213) | C31H44O4 | SBW |
| 5.84_463.3213 | 5.84 | 463.3213 | [M+Li]+ | 456.3051 | 0 | 24,24-Difluoro-1,25,26-trihydroxyvitamin D3 | C26H42F2O4 | SBW |
| 5.84_463.3213 | 5.84 | 463.3213 | [M+H]+ | 462.3168 | 5 | (6R)-6-methylvitamin D3 6,19-sulfur dioxide adduct / (6R)-6-methylcholecalciferol 6,19-sulfur dioxide adduct | C28H46O3S | SBW |
| 5.84_463.3213 | 5.84 | 463.3213 | [M+H]+ | 462.3168 | 5 | (6S)-6-methylvitamin D3 6,19-sulfur dioxide adduct / (6S)-6-methylcholecalciferol 6,19-sulfur dioxide adduct | C28H46O3S | SBW |
| 5.84_463.3213 | 5.84 | 463.3213 | [M+H]+ | 462.3168 | 5 | 1α;,25-dihydroxy-24α,24β-didihomo-22-thia-20-epivitamin D3 / 1α;,25-dihydroxy-24α,24β-didihomo-22-thia-20-epicholecalciferol | C28H46O3S | SBW |
| 5.84_463.3213 | 5.84 | 463.3213 | [M+H]+ | 462.3168 | 5 | 1α;,25-dihydroxy-24α,2β-dihomo-22-thiavitamin D3 / 1α;,25-dihydroxy-24α,24β-dihomo-22-thiacholecalciferol | C28H46O3S | SBW |
| 5.84_463.3213 | 5.84 | 463.3213 | [M+H]+ | 462.3168 | 5 | 1α;,25-dihydroxy-26,27-dimethyl-22-thiavitamin D3 / 1α;,25-dihydroxy-26,27-dimethyl-22-thiacholecalciferol | C28H46O3S | SBW |
| 5.84_463.3213 | 5.84 | 463.3213 | [M+H]+ | 462.3168 | 5 | 1α;,25-dihydroxy-26,27-dimethyl-22-thia-20-epivitamin D3 / 1α;,25-dihydroxy-26,27-dimethyl-22-thia-20-epicholecalciferol | C28H46O3S | SBW |
| 5.84_463.3213 | 5.84 | 463.3213 | [M+Na]+ | 440.3290 | 6 | 11α;-ethynyl-1α;,25-dihydroxyvitamin D3 / 11α;-ethynyl-1α;,25-dihydroxycholecalciferol | C29H44O3 | SBW |
| 5.84_463.3213 | 5.84 | 463.3213 | [M+Na]+ | 440.3290 | 6 | (22E,24E)-1α;,25-dihydroxy-22,23,24,24α-tetradehydro-24α,24β-dihomovitamin D3 / (22E,24E)-1α;,25-dihydroxy-22,23,24,24α-tetradehydro-24α,24β-dihomocholecalciferol | C29H44O3 | SBW |
| 5.84_463.3213 | 5.84 | 463.3213 | [M+Na]+ | 440.3290 | 6 | 1α;,25-dihydroxy-26,27-dimethyl-22,22,23,23-tetradehydrovitamin D3 / 1α;,25-dihydroxy-26,27-dimethyl-22,22,23,23-tetradehydro-cholecalciferol | C29H44O3 | SBW |
| 5.84_463.3213 | 5.84 | 463.3213 | [M+Na]+ | 440.3290 | 6 | 1α;,25-dihydroxy-26,27-dimethyl-22,22,23,23-tetradehydro-20-epivitamin D3 / 1α;,25-dihydroxy-26,27-dimethyl-22,22,23,23-tetradehydro-20-epicholecalciferol | C29H44O3 | SBW |
| 5.84_463.3213 | 5.84 | 463.3213 | [M+Na]+ | 440.3290 | 6 | (20S)-20-cyclopropyl-1α;,25-dihydroxy-16,17-didehydro-21-norvitamin D3 / (20S)-20-cyclopropyl-1α;,25-dihydroxy-16,17-didehydro-21-norcholecalciferol | C29H44O3 | SBW |
| 5.84_463.3213 | 5.84 | 463.3213 | [M+Na]+ | 440.3290 | 6 | 24α,24β-Dihomo-9,10-secocholesta-5,7,10(19),24α-tetraen-1α;,3,25-triol | C29H44O3 | SBW |
| 4.2_496.3345 | 4.20 | 496.3345 | [M+CH3OH+H]+ | 463.3006 | 0 | Aminoglycoside derivative (structurally similar to Gentamicin C2) | C20H41N5O7 | HBW |
| 3.95_520.3216 | 3.95 | 520.3216 | [M+Na]+ | 497.3353 | 5 | Acyl proline derivative (structurally similar to Tumonoic Acid I) | C27H47NO7 | HBW |
| 6.99_573.4888 | 6.99 | 573.4888 | [M+H-H2O]+ | 590.4910 | 0 | DG(17:1(9Z)/17:2(9Z,12Z)/0:0)[iso2] | C37H66O5 | HBW |
| 6.99_573.4888 | 6.99 | 573.4888 | [M+H-H2O]+ | 590.4910 | 0 | DG(16:1(9Z)/18:2(9Z,12Z)/0:0)[iso2] | C37H66O5 | HBW |
| 6.99_573.4888 | 6.99 | 573.4888 | [M+H-H2O]+ | 590.4910 | 0 | DG(16:0/18:3(9Z,12Z,15Z)/0:0)[iso2] | C37H66O5 | HBW |
| 6.99_573.4888 | 6.99 | 573.4888 | [M+H-H2O]+ | 590.4910 | 0 | DG(14:0/20:3(5Z,8Z,11Z)/0:0) | C37H66O5 | HBW |
| 6.99_573.4888 | 6.99 | 573.4888 | [M+H-H2O]+ | 590.4910 | 0 | DG(14:0/20:3(8Z,11Z,14Z)/0:0) | C37H66O5 | HBW |
| 6.99_573.4888 | 6.99 | 573.4888 | [M+H-H2O]+ | 590.4910 | 0 | DG(14:1(9Z)/20:2(11Z,14Z)/0:0) | C37H66O5 | HBW |
| 6.99_573.4888 | 6.99 | 573.4888 | [M+H-H2O]+ | 590.4910 | 0 | DG(16:0/18:3(6Z,9Z,12Z)/0:0) | C37H66O5 | HBW |
| 6.99_573.4888 | 6.99 | 573.4888 | [M+H-H2O]+ | 590.4910 | 0 | DG(18:2(9Z,12Z)/16:1(9Z)/0:0) | C37H66O5 | HBW |
| 6.99_573.4888 | 6.99 | 573.4888 | [M+H-H2O]+ | 590.4910 | 0 | DG(18:3(6Z,9Z,12Z)/16:0/0:0) | C37H66O5 | HBW |
| 6.99_573.4888 | 6.99 | 573.4888 | [M+H-H2O]+ | 590.4910 | 0 | DG(18:3(9Z,12Z,15Z)/16:0/0:0) | C37H66O5 | HBW |
| 6.99_573.4888 | 6.99 | 573.4888 | [M+H-H2O]+ | 590.4910 | 0 | DG(20:2(11Z,14Z)/14:1(9Z)/0:0) | C37H66O5 | HBW |
| 6.99_573.4888 | 6.99 | 573.4888 | [M+H-H2O]+ | 590.4910 | 0 | DG(20:3(5Z,8Z,11Z)/14:0/0:0) | C37H66O5 | HBW |
| 6.99_573.4888 | 6.99 | 573.4888 | [M+H-H2O]+ | 590.4910 | 0 | DG(20:3(8Z,11Z,14Z)/14:0/0:0) | C37H66O5 | HBW |
| 6.99_573.4888 | 6.99 | 573.4888 | [M+Na]+ | 550.4961 | 6 | DG(P-14:0/18:1(9Z)) | C35H66O4 | HBW |
| 7.01_591.4992 | 7.01 | 591.4992 | [M+H]+ | 590.4910 | 1 | DG(17:1(9Z)/17:2(9Z,12Z)/0:0)[iso2] | C37H66O5 | HBW |
| 7.01_591.4992 | 7.01 | 591.4992 | [M+H]+ | 590.4910 | 1 | DG(16:1(9Z)/18:2(9Z,12Z)/0:0)[iso2] | C37H66O5 | HBW |
| 7.01_591.4992 | 7.01 | 591.4992 | [M+H]+ | 590.4910 | 1 | DG(16:0/18:3(9Z,12Z,15Z)/0:0)[iso2] | C37H66O5 | HBW |
| 7.01_591.4992 | 7.01 | 591.4992 | [M+H]+ | 590.4910 | 1 | DG(14:0/20:3(5Z,8Z,11Z)/0:0) | C37H66O5 | HBW |
| 7.01_591.4992 | 7.01 | 591.4992 | [M+H]+ | 590.4910 | 1 | DG(14:0/20:3(8Z,11Z,14Z)/0:0) | C37H66O5 | HBW |
| 7.01_591.4992 | 7.01 | 591.4992 | [M+H]+ | 590.4910 | 1 | DG(14:1(9Z)/20:2(11Z,14Z)/0:0) | C37H66O5 | HBW |
| 7.01_591.4992 | 7.01 | 591.4992 | [M+H]+ | 590.4910 | 1 | DG(16:0/18:3(6Z,9Z,12Z)/0:0) | C37H66O5 | HBW |
| 7.01_591.4992 | 7.01 | 591.4992 | [M+H]+ | 590.4910 | 1 | DG(18:2(9Z,12Z)/16:1(9Z)/0:0) | C37H66O5 | HBW |
| 7.01_591.4992 | 7.01 | 591.4992 | [M+H]+ | 590.4910 | 1 | DG(18:3(6Z,9Z,12Z)/16:0/0:0) | C37H66O5 | HBW |
| 7.01_591.4992 | 7.01 | 591.4992 | [M+H]+ | 590.4910 | 1 | DG(18:3(9Z,12Z,15Z)/16:0/0:0) | C37H66O5 | HBW |
| 7.01_591.4992 | 7.01 | 591.4992 | [M+H]+ | 590.4910 | 1 | DG(20:2(11Z,14Z)/14:1(9Z)/0:0) | C37H66O5 | HBW |
| 7.01_591.4992 | 7.01 | 591.4992 | [M+H]+ | 590.4910 | 1 | DG(20:3(5Z,8Z,11Z)/14:0/0:0) | C37H66O5 | HBW |
| 7.01_591.4992 | 7.01 | 591.4992 | [M+H]+ | 590.4910 | 1 | DG(20:3(8Z,11Z,14Z)/14:0/0:0) | C37H66O5 | HBW |
| 7.01_591.4992 | 7.01 | 591.4992 | [M+Na]+ | 568.5067 | 5 | DG(16:0/16:0/0:0) | C35H68O5 | HBW |
| 7.01_591.4992 | 7.01 | 591.4992 | [M+Na]+ | 568.5067 | 5 | DG(14:0/18:0/0:0) | C35H68O5 | HBW |
| 7.01_591.4992 | 7.01 | 591.4992 | [M+Na]+ | 568.5067 | 5 | DG(18:0/14:0/0:0) | C35H68O5 | HBW |
| 6.70_647.4886 | 6.70 | 647.4886 | NC |  |  |  |  | HBW |
| 6.81_655.4929 | 6.81 | 655.4929 | [M+Li]+ | 648.4730 | 5 | PA(16:0/16:0)[rac] | C35H69O8P | HBW |
| 6.05_665.4967 | 6.05 | 665.4967 | [M+H-2H2O]+ | 700.5043 | 7 | PA(18:1(9Z)/18:1(9Z))[U] | C39H73O8P | HBW |
| 6.05_665.4967 | 6.05 | 665.4967 | [M+H-2H2O]+ | 700.5043 | 7 | PA(18:0/18:2(9Z,12Z)) | C39H73O8P | HBW |
| 6.05_665.4967 | 6.05 | 665.4967 | [M+H-2H2O]+ | 700.5043 | 7 | 1,2-dioleoyl-sn-Glycero-3-Phosphate | C39H73O8P | HBW |
| 6.05_665.4967 | 6.05 | 665.4967 | [M+H-2H2O]+ | 700.5043 | 7 | PA(18:1(11Z)/18:1(11Z)) | C39H73O8P | HBW |
| 6.05_665.4967 | 6.05 | 665.4967 | [M+H-2H2O]+ | 700.5043 | 7 | PA(18:1(11Z)/18:1(9Z)) | C39H73O8P | HBW |
| 6.05_665.4967 | 6.05 | 665.4967 | [M+H-2H2O]+ | 700.5043 | 7 | PA(18:1(9Z)/18:1(11Z)) | C39H73O8P | HBW |
| 6.05_665.4967 | 6.05 | 665.4967 | [M+H-2H2O]+ | 700.5043 | 7 | Dioleoylphosphatidic acid | C39H73O8P | HBW |
| 5.81_689.4986 | 5.81 | 689.4986 | [M+H-2H2O]+ | 724.5129 | 2 | PG(17:0/14:1(9Z)) | C37H75NO10P | HBW |
| 6.14_691.5134 | 6.14 | 691.5134 | NC |  |  |  |  | HBW |
| 6.9_738.5075 | 6.90 | 738.5075 | [M+H]+ | 737.4996 | 0 | PE(14:0/22:5(4Z,7Z,10Z,13Z,16Z)) | C41H72NO8P | SBW |
| 6.9_738.5075 | 6.90 | 738.5075 | [M+H]+ | 737.4996 | 0 | PE(14:0/22:5(7Z,10Z,13Z,16Z,19Z)) | C41H72NO8P | SBW |
| 6.9_738.5075 | 6.90 | 738.5075 | [M+H]+ | 737.4996 | 0 | PE(14:1(9Z)/22:4(7Z,10Z,13Z,16Z)) | C41H72NO8P | SBW |
| 6.9_738.5075 | 6.90 | 738.5075 | [M+H]+ | 737.4996 | 0 | PE(16:0/20:5(5Z,8Z,11Z,14Z,17Z)) | C41H72NO8P | SBW |
| 6.9_738.5075 | 6.90 | 738.5075 | [M+H]+ | 737.4996 | 0 | PE(16:1(9Z)/20:4(5Z,8Z,11Z,14Z)) | C41H72NO8P | SBW |
| 6.9_738.5075 | 6.90 | 738.5075 | [M+H]+ | 737.4996 | 0 | PE(16:1(9Z)/20:4(8Z,11Z,14Z,17Z)) | C41H72NO8P | SBW |
| 6.9_738.5075 | 6.90 | 738.5075 | [M+H]+ | 737.4996 | 0 | PE(18:1(11Z)/18:4(6Z,9Z,12Z,15Z)) | C41H72NO8P | SBW |
| 6.9_738.5075 | 6.90 | 738.5075 | [M+H]+ | 737.4996 | 0 | PE(18:1(9Z)/18:4(6Z,9Z,12Z,15Z)) | C41H72NO8P | SBW |
| 6.9_738.5075 | 6.90 | 738.5075 | [M+H]+ | 737.4996 | 0 | PE(18:2(9Z,12Z)/18:3(6Z,9Z,12Z)) | C41H72NO8P | SBW |
| 6.9_738.5075 | 6.90 | 738.5075 | [M+H]+ | 737.4996 | 0 | PE(18:2(9Z,12Z)/18:3(9Z,12Z,15Z)) | C41H72NO8P | SBW |
| 6.9_738.5075 | 6.90 | 738.5075 | [M+H]+ | 737.4996 | 0 | PE(18:3(6Z,9Z,12Z)/18:2(9Z,12Z)) | C41H72NO8P | SBW |
| 6.9_738.5075 | 6.90 | 738.5075 | [M+H]+ | 737.4996 | 0 | PE(18:3(9Z,12Z,15Z)/18:2(9Z,12Z)) | C41H72NO8P | SBW |
| 6.9_738.5075 | 6.90 | 738.5075 | [M+H]+ | 737.4996 | 0 | PE(18:4(6Z,9Z,12Z,15Z)/18:1(11Z)) | C41H72NO8P | SBW |
| 6.9_738.5075 | 6.90 | 738.5075 | [M+H]+ | 737.4996 | 0 | PE(18:4(6Z,9Z,12Z,15Z)/18:1(9Z)) | C41H72NO8P | SBW |
| 6.9_738.5075 | 6.90 | 738.5075 | [M+H]+ | 737.4996 | 0 | PE(20:4(5Z,8Z,11Z,14Z)/16:1(9Z)) | C41H72NO8P | SBW |
| 6.9_738.5075 | 6.90 | 738.5075 | [M+H]+ | 737.4996 | 0 | PE(20:4(8Z,11Z,14Z,17Z)/16:1(9Z)) | C41H72NO8P | SBW |
| 6.9_738.5075 | 6.90 | 738.5075 | [M+H]+ | 737.4996 | 0 | PE(20:5(5Z,8Z,11Z,14Z,17Z)/16:0) | C41H72NO8P | SBW |
| 6.9_738.5075 | 6.90 | 738.5075 | [M+H]+ | 737.4996 | 0 | PE(22:4(7Z,10Z,13Z,16Z)/14:1(9Z)) | C41H72NO8P | SBW |
| 6.9_738.5075 | 6.90 | 738.5075 | [M+H]+ | 737.4996 | 0 | PE(22:5(4Z,7Z,10Z,13Z,16Z)/14:0) | C41H72NO8P | SBW |
| 6.9_738.5075 | 6.90 | 738.5075 | [M+H]+ | 737.4996 | 0 | PE(22:5(7Z,10Z,13Z,16Z,19Z)/14:0) | C41H72NO8P | SBW |
| 6.9_738.5075 | 6.90 | 738.5075 | [M+Na]+ | 715.5152 | 4 | PE(16:0/18:2(9Z,12Z)) | C39H74NO8P | SBW |
| 6.9_738.5075 | 6.90 | 738.5075 | [M+Na]+ | 715.5152 | 4 | 1-Palmitoyl-2-linoleoyl PE | C39H74NO8P | SBW |
| 6.9_738.5075 | 6.90 | 738.5075 | [M+Na]+ | 715.5152 | 4 | PE(14:0/20:2(11Z,14Z)) | C39H74NO8P | SBW |
| 6.9_738.5075 | 6.90 | 738.5075 | [M+Na]+ | 715.5152 | 4 | PE(14:1(9Z)/20:1(11Z)) | C39H74NO8P | SBW |
| 6.9_738.5075 | 6.90 | 738.5075 | [M+Na]+ | 715.5152 | 4 | PE(16:1(9Z)/18:1(11Z)) | C39H74NO8P | SBW |
| 6.9_738.5075 | 6.90 | 738.5075 | [M+Na]+ | 715.5152 | 4 | PE(16:1(9Z)/18:1(9Z)) | C39H74NO8P | SBW |
| 6.9_738.5075 | 6.90 | 738.5075 | [M+Na]+ | 715.5152 | 4 | PE(18:1(11Z)/16:1(9Z)) | C39H74NO8P | SBW |
| 6.9_738.5075 | 6.90 | 738.5075 | [M+Na]+ | 715.5152 | 4 | PE(18:1(9Z)/16:1(9Z)) | C39H74NO8P | SBW |
| 6.9_738.5075 | 6.90 | 738.5075 | [M+Na]+ | 715.5152 | 4 | PE(18:2(9Z,12Z)/16:0) | C39H74NO8P | SBW |
| 6.9_738.5075 | 6.90 | 738.5075 | [M+Na]+ | 715.5152 | 4 | PE(20:1(11Z)/14:1(9Z)) | C39H74NO8P | SBW |
| 6.9_738.5075 | 6.90 | 738.5075 | [M+Na]+ | 715.5152 | 4 | PE(20:2(11Z,14Z)/14:0) | C39H74NO8P | SBW |
| 7.15_740.5198 | 7.15 | 740.5198 | [M+Na]+ | 717.5308 | 0 | PE(16:0/18:1(9Z)) | C39H76NO8P | SBW |
| 7.15_740.5198 | 7.15 | 740.5198 | [M+Na]+ | 717.5308 | 0 | PE(18:0/18:1(11Z)) | C39H76NO8P | SBW |
| 7.15_740.5198 | 7.15 | 740.5198 | [M+Na]+ | 717.5308 | 0 | PE(18:1(9Z)/16:0) | C39H76NO8P | SBW |
| 7.15_740.5198 | 7.15 | 740.5198 | [M+Na]+ | 717.5308 | 0 | PE(18:1(9Z)/16:0)[U] | C39H76NO8P | SBW |
| 7.15_740.5198 | 7.15 | 740.5198 | [M+Na]+ | 717.5308 | 0 | PE(16:0/18:1(13Z))[U] | C39H76NO8P | SBW |
| 7.15_740.5198 | 7.15 | 740.5198 | [M+Na]+ | 717.5308 | 0 | PE(16:0/18:1(7Z)) | C39H76NO8P | SBW |
| 7.15_740.5198 | 7.15 | 740.5198 | [M+Na]+ | 717.5309 | 0 | PC(16:0/15:1(14)) | C39H76NO8P | SBW |
| 7.15_740.5198 | 7.15 | 740.5198 | [M+Na]+ | 717.5309 | 0 | PC(17:0/14:1(9Z)) | C39H76NO8P | SBW |
| 7.15_740.5198 | 7.15 | 740.5198 | [M+Na]+ | 717.5309 | 0 | PC(15:0/16:1(7Z))[U] | C39H76NO8P | SBW |
| 7.15_740.5198 | 7.15 | 740.5198 | [M+Na]+ | 717.5309 | 0 | PC(15:0/16:1(9Z)) | C39H76NO8P | SBW |
| 7.15_740.5198 | 7.15 | 740.5198 | [M+Na]+ | 717.5309 | 0 | PC(15:0/16:1(9Z))[U] | C39H76NO8P | SBW |
| 7.15_740.5198 | 7.15 | 740.5198 | [M+Na]+ | 717.5309 | 0 | PC(15:0/16:1(9Z)) | C39H76NO8P | SBW |
| 7.15_740.5198 | 7.15 | 740.5198 | [M+Na]+ | 717.5309 | 0 | PC(16:1(9Z)/15:0) | C39H76NO8P | SBW |
| 7.15_740.5198 | 7.15 | 740.5198 | [M+Na]+ | 717.5309 | 0 | PE(14:0/20:1(11Z)) | C39H76NO8P | SBW |
| 7.15_740.5198 | 7.15 | 740.5198 | [M+Na]+ | 717.5309 | 0 | PE(14:1(9Z)/20:0) | C39H76NO8P | SBW |
| 7.15_740.5198 | 7.15 | 740.5198 | [M+Na]+ | 717.5309 | 0 | PE(16:1(9Z)/18:0) | C39H76NO8P | SBW |
| 7.15_740.5198 | 7.15 | 740.5198 | [M+Na]+ | 717.5309 | 0 | PE(18:0/16:1(9Z)) | C39H76NO8P | SBW |
| 7.15_740.5198 | 7.15 | 740.5198 | [M+Na]+ | 717.5309 | 0 | PE(18:1(11Z)/16:0) | C39H76NO8P | SBW |
| 7.15_740.5198 | 7.15 | 740.5198 | [M+Na]+ | 717.5309 | 0 | PE(20:0/14:1(9Z)) | C39H76NO8P | SBW |
| 7.15_740.5198 | 7.15 | 740.5198 | [M+Na]+ | 717.5309 | 0 | PE(20:1(11Z)/14:0) | C39H76NO8P | SBW |
| 7.15_740.5198 | 7.15 | 740.5198 | [M+Na]+ | 717.5309 | 0 | PE(16:0/18:1(9Z)) | C39H76NO8P | SBW |
| 7.15_740.5198 | 7.15 | 740.5198 | [M+H]+ | 739.5152 | 3 | PE(18:2(9Z,12Z)/18:2(9Z,12Z))[U] | C41H74NO8P | SBW |
| 7.15_740.5198 | 7.15 | 740.5198 | [M+H]+ | 739.5152 | 3 | PE(18:2(9Z,11Z)/18:2(9Z,11Z))[U] | C41H74NO8P | SBW |
| 7.15_740.5198 | 7.15 | 740.5198 | [M+H]+ | 739.5152 | 3 | PE(16:0/20:4(5Z,8Z,11Z,14Z)) | C41H74NO8P | SBW |
| 7.15_740.5198 | 7.15 | 740.5198 | [M+H]+ | 739.5152 | 3 | PE(18:2(9Z,12Z)/18:2(9Z,12Z)) | C41H74NO8P | SBW |
| 7.15_740.5198 | 7.15 | 740.5198 | [M+H]+ | 739.5152 | 3 | PE(18:2(6Z,9Z)/18:2(6Z,9Z)) | C41H74NO8P | SBW |
| 7.15_740.5198 | 7.15 | 740.5198 | [M+H]+ | 739.5152 | 3 | PE(18:2(6Z,9Z)/18:2(6Z,9Z))[U] | C41H74NO8P | SBW |
| 7.15_740.5198 | 7.15 | 740.5198 | [M+H]+ | 739.5152 | 3 | PC(15:0/18:4(6Z,9Z,12Z,15Z)) | C41H74NO8P | SBW |
| 7.15_740.5198 | 7.15 | 740.5198 | [M+H]+ | 739.5152 | 3 | PC(18:4(6Z,9Z,12Z,15Z)/15:0) | C41H74NO8P | SBW |
| 7.15_740.5198 | 7.15 | 740.5198 | [M+H]+ | 739.5152 | 3 | PE(14:0/22:4(7Z,10Z,13Z,16Z)) | C41H74NO8P | SBW |
| 7.15_740.5198 | 7.15 | 740.5198 | [M+H]+ | 739.5152 | 3 | PE(16:0/20:4(8Z,11Z,14Z,17Z)) | C41H74NO8P | SBW |
| 7.15_740.5198 | 7.15 | 740.5198 | [M+H]+ | 739.5152 | 3 | PE(16:1(9Z)/20:3(5Z,8Z,11Z)) | C41H74NO8P | SBW |
| 7.15_740.5198 | 7.15 | 740.5198 | [M+H]+ | 739.5152 | 3 | PE(18:0/18:4(6Z,9Z,12Z,15Z)) | C41H74NO8P | SBW |
| 7.15_740.5198 | 7.15 | 740.5198 | [M+H]+ | 739.5152 | 3 | PE(18:1(11Z)/18:3(6Z,9Z,12Z)) | C41H74NO8P | SBW |
| 7.15_740.5198 | 7.15 | 740.5198 | [M+H]+ | 739.5152 | 3 | PE(18:1(11Z)/18:3(9Z,12Z,15Z)) | C41H74NO8P | SBW |
| 7.15_740.5198 | 7.15 | 740.5198 | [M+H]+ | 739.5152 | 3 | PE(18:1(9Z)/18:3(6Z,9Z,12Z)) | C41H74NO8P | SBW |
| 7.15_740.5198 | 7.15 | 740.5198 | [M+H]+ | 739.5152 | 3 | PE(18:1(9Z)/18:3(9Z,12Z,15Z)) | C41H74NO8P | SBW |
| 7.15_740.5198 | 7.15 | 740.5198 | [M+H]+ | 739.5152 | 3 | PE(18:3(6Z,9Z,12Z)/18:1(11Z)) | C41H74NO8P | SBW |
| 7.15_740.5198 | 7.15 | 740.5198 | [M+H]+ | 739.5152 | 3 | PE(18:3(6Z,9Z,12Z)/18:1(9Z)) | C41H74NO8P | SBW |
| 7.15_740.5198 | 7.15 | 740.5198 | [M+H]+ | 739.5152 | 3 | PE(18:3(9Z,12Z,15Z)/18:1(11Z)) | C41H74NO8P | SBW |
| 7.15_740.5198 | 7.15 | 740.5198 | [M+H]+ | 739.5152 | 3 | PE(18:3(9Z,12Z,15Z)/18:1(9Z)) | C41H74NO8P | SBW |
| 7.15_740.5198 | 7.15 | 740.5198 | [M+H]+ | 739.5152 | 3 | PE(18:4(6Z,9Z,12Z,15Z)/18:0) | C41H74NO8P | SBW |
| 7.15_740.5198 | 7.15 | 740.5198 | [M+H]+ | 739.5152 | 3 | PE(20:3(5Z,8Z,11Z)/16:1(9Z)) | C41H74NO8P | SBW |
| 7.15_740.5198 | 7.15 | 740.5198 | [M+H]+ | 739.5152 | 3 | PE(20:3(8Z,11Z,14Z)/16:1(9Z)) | C41H74NO8P | SBW |
| 7.15_740.5198 | 7.15 | 740.5198 | [M+H]+ | 739.5152 | 3 | PE(20:4(5Z,8Z,11Z,14Z)/16:0) | C41H74NO8P | SBW |
| 7.15_740.5198 | 7.15 | 740.5198 | [M+H]+ | 739.5152 | 3 | PE(20:4(8Z,11Z,14Z,17Z)/16:0) | C41H74NO8P | SBW |
| 7.15_740.5198 | 7.15 | 740.5198 | [M+H]+ | 739.5152 | 3 | PE(22:4(7Z,10Z,13Z,16Z)/14:0) | C41H74NO8P | SBW |
| 7.15_740.5198 | 7.15 | 740.5198 | [M+2H]2+ | 1479.0192 | 3 | CL(20:2(11Z,14Z)/18:2(9Z,12Z)/18:2(9Z,12Z)/18:1(11Z)) | C83H148O17P2 | SBW |
| 7.15_740.5198 | 7.15 | 740.5198 | [M+2H]2+ | 1479.0192 | 3 | CL(20:2(11Z,14Z)/18:2(9Z,12Z)/18:2(9Z,12Z)/18:1(9Z)) | C83H148O17P2 | SBW |
| 7.03_756.5511 | 7.03 | 756.5511 | [M+Na]+ | 733.5621 | 0 | PE(17:0/18:0)[U] | C40H80NO8P | SBW |
| 7.03_756.5511 | 7.03 | 756.5511 | [M+Na]+ | 733.5621 | 0 | PE(19:0/16:0) | C40H80NO8P | SBW |
| 7.03_756.5511 | 7.03 | 756.5511 | [M+Na]+ | 733.5621 | 0 | PE(19:0/16:0)[U] | C40H80NO8P | SBW |
| 7.03_756.5511 | 7.03 | 756.5511 | [M+Na]+ | 733.5621 | 0 | PE(16:0/19:0) | C40H80NO8P | SBW |
| 7.03_756.5511 | 7.03 | 756.5511 | [M+Na]+ | 733.5621 | 0 | PE(16:0/19:0)[U] | C40H80NO8P | SBW |
| 7.03_756.5511 | 7.03 | 756.5511 | [M+Na]+ | 733.5621 | 0 | PE(20:0/15:0)[U] | C40H80NO8P | SBW |
| 7.03_756.5511 | 7.03 | 756.5511 | [M+Na]+ | 733.5621 | 0 | PE(18:0/17:0)[U] | C40H80NO8P | SBW |
| 7.03_756.5511 | 7.03 | 756.5511 | [M+Na]+ | 733.5621 | 0 | PE(13:0/22:0)[U] | C40H80NO8P | SBW |
| 7.03_756.5511 | 7.03 | 756.5511 | [M+Na]+ | 733.5621 | 0 | PE(14:0/21:0)[U] | C40H80NO8P | SBW |
| 7.03_756.5511 | 7.03 | 756.5511 | [M+Na]+ | 733.5621 | 0 | PE-NMe(17:0/17:0)[U] | C40H80NO8P | SBW |
| 7.03_756.5511 | 7.03 | 756.5511 | [M+Na]+ | 733.5622 | 0 | PC(10:0/22:0) | C40H80NO8P | SBW |
| 7.03_756.5511 | 7.03 | 756.5511 | [M+Na]+ | 733.5622 | 0 | PC(10:0/22:0)[U] | C40H80NO8P | SBW |
| 7.03_756.5511 | 7.03 | 756.5511 | [M+Na]+ | 733.5622 | 0 | PC(11:0/21:0) | C40H80NO8P | SBW |
| 7.03_756.5511 | 7.03 | 756.5511 | [M+Na]+ | 733.5622 | 0 | PC(12:0/20:0) | C40H80NO8P | SBW |
| 7.03_756.5511 | 7.03 | 756.5511 | [M+Na]+ | 733.5622 | 0 | PC(12:0/20:0)[U] | C40H80NO8P | SBW |
| 7.03_756.5511 | 7.03 | 756.5511 | [M+Na]+ | 733.5622 | 0 | PC(13:0/19:0) | C40H80NO8P | SBW |
| 7.03_756.5511 | 7.03 | 756.5511 | [M+Na]+ | 733.5622 | 0 | PC(13:0/19:0)[U] | C40H80NO8P | SBW |
| 7.03_756.5511 | 7.03 | 756.5511 | [M+Na]+ | 733.5622 | 0 | PC(14:0/18:0) | C40H80NO8P | SBW |
| 7.03_756.5511 | 7.03 | 756.5511 | [M+Na]+ | 733.5622 | 0 | PC(14:0/18:0)[U] | C40H80NO8P | SBW |
| 7.03_756.5511 | 7.03 | 756.5511 | [M+Na]+ | 733.5622 | 0 | PC(15:0/17:0) | C40H80NO8P | SBW |
| 7.03_756.5511 | 7.03 | 756.5511 | [M+Na]+ | 733.5622 | 0 | PC(15:0/17:0)[U] | C40H80NO8P | SBW |
| 7.03_756.5511 | 7.03 | 756.5511 | [M+Na]+ | 733.5622 | 0 | PC(16:0/16:0) | C40H80NO8P | SBW |
| 7.03_756.5511 | 7.03 | 756.5511 | [M+Na]+ | 733.5622 | 0 | PC(16:0/16:0)[U] | C40H80NO8P | SBW |
| 7.03_756.5511 | 7.03 | 756.5511 | [M+Na]+ | 733.5622 | 0 | PC(17:0/15:0) | C40H80NO8P | SBW |
| 7.03_756.5511 | 7.03 | 756.5511 | [M+Na]+ | 733.5622 | 0 | PC(17:0/15:0)[U] | C40H80NO8P | SBW |
| 7.03_756.5511 | 7.03 | 756.5511 | [M+Na]+ | 733.5622 | 0 | PC(18:0/14:0) | C40H80NO8P | SBW |
| 7.03_756.5511 | 7.03 | 756.5511 | [M+Na]+ | 733.5622 | 0 | PC(18:0/14:0)[U] | C40H80NO8P | SBW |
| 7.03_756.5511 | 7.03 | 756.5511 | [M+Na]+ | 733.5622 | 0 | PC(19:0/13:0) | C40H80NO8P | SBW |
| 7.03_756.5511 | 7.03 | 756.5511 | [M+Na]+ | 733.5622 | 0 | PC(20:0/12:0) | C40H80NO8P | SBW |
| 7.03_756.5511 | 7.03 | 756.5511 | [M+Na]+ | 733.5622 | 0 | PC(20:0/12:0)[U] | C40H80NO8P | SBW |
| 7.03_756.5511 | 7.03 | 756.5511 | [M+Na]+ | 733.5622 | 0 | PC(21:0/11:0) | C40H80NO8P | SBW |
| 7.03_756.5511 | 7.03 | 756.5511 | [M+Na]+ | 733.5622 | 0 | PC(22:0/10:0) | C40H80NO8P | SBW |
| 7.03_756.5511 | 7.03 | 756.5511 | [M+Na]+ | 733.5622 | 0 | PC(9:0/23:0) | C40H80NO8P | SBW |
| 7.03_756.5511 | 7.03 | 756.5511 | [M+Na]+ | 733.5622 | 0 | 1,2-dipalmitoyl-sn-glycero-3-PC | C40H80NO8P | SBW |
| 7.03_756.5511 | 7.03 | 756.5511 | [M+Na]+ | 733.5622 | 0 | PE(18:0(10(R)Me)/16:0) | C40H80NO8P | SBW |
| 7.03_756.5511 | 7.03 | 756.5511 | [M+Na]+ | 733.5622 | 0 | PC(14:0/18:0) | C40H80NO8P | SBW |
| 7.03_756.5511 | 7.03 | 756.5511 | [M+Na]+ | 733.5622 | 0 | PC(18:0/14:0) | C40H80NO8P | SBW |
| 7.03_756.5511 | 7.03 | 756.5511 | [M+Na]+ | 733.5622 | 0 | PE(15:0/20:0) | C40H80NO8P | SBW |
| 7.03_756.5511 | 7.03 | 756.5511 | [M+Na]+ | 733.5622 | 0 | PE(20:0/15:0) | C40H80NO8P | SBW |
| 7.03_756.5511 | 7.03 | 756.5511 | [M+Li]+ | 749.5359 | 1 | PE(20:4(5Z,8Z,11Z,14Z)/P-18:1(11Z)) | C43H76NO7P | SBW |
| 7.03_756.5511 | 7.03 | 756.5511 | [M+Li]+ | 749.5359 | 1 | PE(20:4(5Z,8Z,11Z,14Z)/P-18:1(9Z)) | C43H76NO7P | SBW |
| 7.03_756.5511 | 7.03 | 756.5511 | [M+Li]+ | 749.5359 | 1 | PE(20:4(8Z,11Z,14Z,17Z)/P-18:1(11Z)) | C43H76NO7P | SBW |
| 7.03_756.5511 | 7.03 | 756.5511 | [M+Li]+ | 749.5359 | 1 | PE(20:4(8Z,11Z,14Z,17Z)/P-18:1(9Z)) | C43H76NO7P | SBW |
| 7.03_756.5511 | 7.03 | 756.5511 | [M+Li]+ | 749.5359 | 1 | PE(20:5(5Z,8Z,11Z,14Z,17Z)/P-18:0) | C43H76NO7P | SBW |
| 7.03_756.5511 | 7.03 | 756.5511 | [M+Li]+ | 749.5359 | 1 | PE(22:5(4Z,7Z,10Z,13Z,16Z)/P-16:0) | C43H76NO7P | SBW |
| 7.03_756.5511 | 7.03 | 756.5511 | [M+Li]+ | 749.5359 | 1 | PE(22:5(7Z,10Z,13Z,16Z,19Z)/P-16:0) | C43H76NO7P | SBW |
| 7.03_756.5511 | 7.03 | 756.5511 | [M+Li]+ | 749.5359 | 1 | PE(P-16:0/22:5(4Z,7Z,10Z,13Z,16Z)) | C43H76NO7P | SBW |
| 7.03_756.5511 | 7.03 | 756.5511 | [M+Li]+ | 749.5359 | 1 | PE(P-16:0/22:5(7Z,10Z,13Z,16Z,19Z)) | C43H76NO7P | SBW |
| 7.03_756.5511 | 7.03 | 756.5511 | [M+Li]+ | 749.5359 | 1 | PE(P-18:0/20:5(5Z,8Z,11Z,14Z,17Z)) | C43H76NO7P | SBW |
| 7.03_756.5511 | 7.03 | 756.5511 | [M+Li]+ | 749.5359 | 1 | PE(P-18:1(11Z)/20:4(5Z,8Z,11Z,14Z)) | C43H76NO7P | SBW |
| 7.03_756.5511 | 7.03 | 756.5511 | [M+Li]+ | 749.5359 | 1 | PE(P-18:1(11Z)/20:4(8Z,11Z,14Z,17Z)) | C43H76NO7P | SBW |
| 7.03_756.5511 | 7.03 | 756.5511 | [M+Li]+ | 749.5359 | 1 | PE(P-18:1(9Z)/20:4(5Z,8Z,11Z,14Z)) | C43H76NO7P | SBW |
| 7.03_756.5511 | 7.03 | 756.5511 | [M+Li]+ | 749.5359 | 1 | PE(P-18:1(9Z)/20:4(8Z,11Z,14Z,17Z)) | C43H76NO7P | SBW |
| 7.03_756.5511 | 7.03 | 756.5511 | [M+H]+ | 755.5465 | 3 | PC(15:0/19:3(9Z,12Z,15Z))[U] | C42H78NO8P | SBW |
| 7.03_756.5511 | 7.03 | 756.5511 | [M+H]+ | 755.5465 | 3 | PC(16:0/18:3(5E,9Z,12Z))[U] | C42H78NO8P | SBW |
| 7.03_756.5511 | 7.03 | 756.5511 | [M+H]+ | 755.5465 | 3 | PC(16:0/18:3(6Z,9Z,12Z)) | C42H78NO8P | SBW |
| 7.03_756.5511 | 7.03 | 756.5511 | [M+H]+ | 755.5465 | 3 | PC(16:0/18:3(6Z,9Z,12Z))[U] | C42H78NO8P | SBW |
| 7.03_756.5511 | 7.03 | 756.5511 | [M+H]+ | 755.5465 | 3 | PC(16:0/18:3(9E,12E,15E))[U] | C42H78NO8P | SBW |
| 7.03_756.5511 | 7.03 | 756.5511 | [M+H]+ | 755.5465 | 3 | PC(16:0/18:3(9Z,12Z,15Z)) | C42H78NO8P | SBW |
| 7.03_756.5511 | 7.03 | 756.5511 | [M+H]+ | 755.5465 | 3 | PC(16:0/18:3(9Z,12Z,15Z))[U] | C42H78NO8P | SBW |
| 7.03_756.5511 | 7.03 | 756.5511 | [M+H]+ | 755.5465 | 3 | PC(16:1(7Z)/18:2(9Z,12Z))[U] | C42H78NO8P | SBW |
| 7.03_756.5511 | 7.03 | 756.5511 | [M+H]+ | 755.5465 | 3 | PC(16:1(9Z)/18:2(9Z,12Z)) | C42H78NO8P | SBW |
| 7.03_756.5511 | 7.03 | 756.5511 | [M+H]+ | 755.5465 | 3 | PC(16:1(9Z)/18:2(9Z,12Z))[U] | C42H78NO8P | SBW |
| 7.03_756.5511 | 7.03 | 756.5511 | [M+H]+ | 755.5465 | 3 | PC(14:0/20:3(5Z,8Z,11Z)) | C42H78NO8P | SBW |
| 7.03_756.5511 | 7.03 | 756.5511 | [M+H]+ | 755.5465 | 3 | PC(14:0/20:3(8Z,11Z,14Z)) | C42H78NO8P | SBW |
| 7.03_756.5511 | 7.03 | 756.5511 | [M+H]+ | 755.5465 | 3 | PC(14:1(9Z)/20:2(11Z,14Z)) | C42H78NO8P | SBW |
| 7.03_756.5511 | 7.03 | 756.5511 | [M+H]+ | 755.5465 | 3 | PC(16:0/18:3(6Z,9Z,12Z)) | C42H78NO8P | SBW |
| 7.03_756.5511 | 7.03 | 756.5511 | [M+H]+ | 755.5465 | 3 | PC(16:0/18:3(9Z,12Z,15Z)) | C42H78NO8P | SBW |
| 7.03_756.5511 | 7.03 | 756.5511 | [M+H]+ | 755.5465 | 3 | PC(16:1(9Z)/18:2(9Z,12Z)) | C42H78NO8P | SBW |
| 7.03_756.5511 | 7.03 | 756.5511 | [M+H]+ | 755.5465 | 3 | PC(18:2(9Z,12Z)/16:1(9Z)) | C42H78NO8P | SBW |
| 7.03_756.5511 | 7.03 | 756.5511 | [M+H]+ | 755.5465 | 3 | PC(18:3(6Z,9Z,12Z)/16:0) | C42H78NO8P | SBW |
| 7.03_756.5511 | 7.03 | 756.5511 | [M+H]+ | 755.5465 | 3 | PC(18:3(9Z,12Z,15Z)/16:0) | C42H78NO8P | SBW |
| 7.03_756.5511 | 7.03 | 756.5511 | [M+H]+ | 755.5465 | 3 | PC(20:2(11Z,14Z)/14:1(9Z)) | C42H78NO8P | SBW |
| 7.03_756.5511 | 7.03 | 756.5511 | [M+H]+ | 755.5465 | 3 | PC(20:3(5Z,8Z,11Z)/14:0) | C42H78NO8P | SBW |
| 7.03_756.5511 | 7.03 | 756.5511 | [M+H]+ | 755.5465 | 3 | PC(20:3(8Z,11Z,14Z)/14:0) | C42H78NO8P | SBW |
| 7.03_756.5511 | 7.03 | 756.5511 | [M+CH3OH+H]+ | 723.5203 | 3 | PE(18:3(6Z,9Z,12Z)/P-18:1(11Z)) | C41H74NO7P | SBW |
| 7.03_756.5511 | 7.03 | 756.5511 | [M+CH3OH+H]+ | 723.5203 | 3 | PE(18:3(6Z,9Z,12Z)/P-18:1(9Z)) | C41H74NO7P | SBW |
| 7.03_756.5511 | 7.03 | 756.5511 | [M+CH3OH+H]+ | 723.5203 | 3 | PE(18:3(9Z,12Z,15Z)/P-18:1(11Z)) | C41H74NO7P | SBW |
| 7.03_756.5511 | 7.03 | 756.5511 | [M+CH3OH+H]+ | 723.5203 | 3 | PE(18:3(9Z,12Z,15Z)/P-18:1(9Z)) | C41H74NO7P | SBW |
| 7.03_756.5511 | 7.03 | 756.5511 | [M+CH3OH+H]+ | 723.5203 | 3 | PE(18:4(6Z,9Z,12Z,15Z)/P-18:0) | C41H74NO7P | SBW |
| 7.03_756.5511 | 7.03 | 756.5511 | [M+CH3OH+H]+ | 723.5203 | 3 | PE(20:4(5Z,8Z,11Z,14Z)/P-16:0) | C41H74NO7P | SBW |
| 7.03_756.5511 | 7.03 | 756.5511 | [M+CH3OH+H]+ | 723.5203 | 3 | PE(20:4(8Z,11Z,14Z,17Z)/P-16:0) | C41H74NO7P | SBW |
| 7.03_756.5511 | 7.03 | 756.5511 | [M+CH3OH+H]+ | 723.5203 | 3 | PE(P-16:0/20:4(5Z,8Z,11Z,14Z)) | C41H74NO7P | SBW |
| 7.03_756.5511 | 7.03 | 756.5511 | [M+CH3OH+H]+ | 723.5203 | 3 | PE(P-16:0/20:4(8Z,11Z,14Z,17Z)) | C41H74NO7P | SBW |
| 7.03_756.5511 | 7.03 | 756.5511 | [M+CH3OH+H]+ | 723.5203 | 3 | PE(P-18:0/18:4(6Z,9Z,12Z,15Z)) | C41H74NO7P | SBW |
| 7.03_756.5511 | 7.03 | 756.5511 | [M+CH3OH+H]+ | 723.5203 | 3 | PE(P-18:1(11Z)/18:3(6Z,9Z,12Z)) | C41H74NO7P | SBW |
| 7.03_756.5511 | 7.03 | 756.5511 | [M+CH3OH+H]+ | 723.5203 | 3 | PE(P-18:1(11Z)/18:3(9Z,12Z,15Z)) | C41H74NO7P | SBW |
| 7.03_756.5511 | 7.03 | 756.5511 | [M+CH3OH+H]+ | 723.5203 | 3 | PE(P-18:1(9Z)/18:3(6Z,9Z,12Z)) | C41H74NO7P | SBW |
| 7.03_756.5511 | 7.03 | 756.5511 | [M+CH3OH+H]+ | 723.5203 | 3 | PE(P-18:1(9Z)/18:3(9Z,12Z,15Z)) | C41H74NO7P | SBW |
| 7.03_756.5511 | 7.03 | 756.5511 | [M+H-2H2O]+ | 791.5676 | 4 | PS(18:0/18:0)[U] | C42H82NO10P | SBW |
| 7.03_756.5511 | 7.03 | 756.5511 | [M+H-2H2O]+ | 791.5676 | 4 | PS(16:0/20:0) | C42H82NO10P | SBW |
| 7.03_756.5511 | 7.03 | 756.5511 | [M+H-2H2O]+ | 791.5676 | 4 | PS(18:0/18:0) | C42H82NO10P | SBW |
| 7.03_756.5511 | 7.03 | 756.5511 | [M+H-2H2O]+ | 791.5676 | 4 | 1,2-Distearoyl phosphatidyl serine | C42H82NO10P | SBW |
| 7.03_756.5511 | 7.03 | 756.5511 | [M+Li]+ | 749.5393 | 5 | Palmitoyl thio-PC | C40H80NO7PS | SBW |
| 6.4_756.5555 | 6.40 | 756.5555 | [M+H-2H2O]+ | 791.5676 | 0 | PS(18:0/18:0)[U] | C42H82NO10P | HBW |
| 6.4_756.5555 | 6.40 | 756.5555 | [M+H-2H2O]+ | 791.5676 | 0 | PS(16:0/20:0) | C42H82NO10P | HBW |
| 6.4_756.5555 | 6.40 | 756.5555 | [M+H-2H2O]+ | 791.5676 | 0 | PS(18:0/18:0) | C42H82NO10P | HBW |
| 6.4_756.5555 | 6.40 | 756.5555 | [M+H-2H2O]+ | 791.5676 | 0 | 1,2-Distearoyl phosphatidyl serine | C42H82NO10P | HBW |
| 6.4_756.5555 | 6.40 | 756.5555 | [M+Li]+ | 749.5393 | 0 | Palmitoyl thio-PC | C40H80NO7PS | HBW |
| 6.4_756.5555 | 6.40 | 756.5555 | [M+H]+ | 755.5465 | 2 | PC(15:0/19:3(9Z,12Z,15Z))[U] | C42H78NO8P | HBW |
| 6.4_756.5555 | 6.40 | 756.5555 | [M+H]+ | 755.5465 | 2 | PC(16:0/18:3(5E,9Z,12Z))[U] | C42H78NO8P | HBW |
| 6.4_756.5555 | 6.40 | 756.5555 | [M+H]+ | 755.5465 | 2 | PC(16:0/18:3(6Z,9Z,12Z)) | C42H78NO8P | HBW |
| 6.4_756.5555 | 6.40 | 756.5555 | [M+H]+ | 755.5465 | 2 | PC(16:0/18:3(6Z,9Z,12Z))[U] | C42H78NO8P | HBW |
| 6.4_756.5555 | 6.40 | 756.5555 | [M+H]+ | 755.5465 | 2 | PC(16:0/18:3(9E,12E,15E))[U] | C42H78NO8P | HBW |
| 6.4_756.5555 | 6.40 | 756.5555 | [M+H]+ | 755.5465 | 2 | PC(16:0/18:3(9Z,12Z,15Z)) | C42H78NO8P | HBW |
| 6.4_756.5555 | 6.40 | 756.5555 | [M+H]+ | 755.5465 | 2 | PC(16:0/18:3(9Z,12Z,15Z))[U] | C42H78NO8P | HBW |
| 6.4_756.5555 | 6.40 | 756.5555 | [M+H]+ | 755.5465 | 2 | PC(16:1(7Z)/18:2(9Z,12Z))[U] | C42H78NO8P | HBW |
| 6.4_756.5555 | 6.40 | 756.5555 | [M+H]+ | 755.5465 | 2 | PC(16:1(9Z)/18:2(9Z,12Z)) | C42H78NO8P | HBW |
| 6.4_756.5555 | 6.40 | 756.5555 | [M+H]+ | 755.5465 | 2 | PC(16:1(9Z)/18:2(9Z,12Z))[U] | C42H78NO8P | HBW |
| 6.4_756.5555 | 6.40 | 756.5555 | [M+H]+ | 755.5465 | 2 | PC(14:0/20:3(5Z,8Z,11Z)) | C42H78NO8P | HBW |
| 6.4_756.5555 | 6.40 | 756.5555 | [M+H]+ | 755.5465 | 2 | PC(14:0/20:3(8Z,11Z,14Z)) | C42H78NO8P | HBW |
| 6.4_756.5555 | 6.40 | 756.5555 | [M+H]+ | 755.5465 | 2 | PC(14:1(9Z)/20:2(11Z,14Z)) | C42H78NO8P | HBW |
| 6.4_756.5555 | 6.40 | 756.5555 | [M+H]+ | 755.5465 | 2 | PC(16:0/18:3(6Z,9Z,12Z)) | C42H78NO8P | HBW |
| 6.4_756.5555 | 6.40 | 756.5555 | [M+H]+ | 755.5465 | 2 | PC(16:0/18:3(9Z,12Z,15Z)) | C42H78NO8P | HBW |
| 6.4_756.5555 | 6.40 | 756.5555 | [M+H]+ | 755.5465 | 2 | PC(16:1(9Z)/18:2(9Z,12Z)) | C42H78NO8P | HBW |
| 6.4_756.5555 | 6.40 | 756.5555 | [M+H]+ | 755.5465 | 2 | PC(18:2(9Z,12Z)/16:1(9Z)) | C42H78NO8P | HBW |
| 6.4_756.5555 | 6.40 | 756.5555 | [M+H]+ | 755.5465 | 2 | PC(18:3(6Z,9Z,12Z)/16:0) | C42H78NO8P | HBW |
| 6.4_756.5555 | 6.40 | 756.5555 | [M+H]+ | 755.5465 | 2 | PC(18:3(9Z,12Z,15Z)/16:0) | C42H78NO8P | HBW |
| 6.4_756.5555 | 6.40 | 756.5555 | [M+H]+ | 755.5465 | 2 | PC(20:2(11Z,14Z)/14:1(9Z)) | C42H78NO8P | HBW |
| 6.4_756.5555 | 6.40 | 756.5555 | [M+H]+ | 755.5465 | 2 | PC(20:3(5Z,8Z,11Z)/14:0) | C42H78NO8P | HBW |
| 6.4_756.5555 | 6.40 | 756.5555 | [M+H]+ | 755.5465 | 2 | PC(20:3(8Z,11Z,14Z)/14:0) | C42H78NO8P | HBW |
| 6.4_756.5555 | 6.40 | 756.5555 | [M+CH3OH+H]+ | 723.5203 | 2 | PE(18:3(6Z,9Z,12Z)/P-18:1(11Z)) | C41H74NO7P | HBW |
| 6.4_756.5555 | 6.40 | 756.5555 | [M+CH3OH+H]+ | 723.5203 | 2 | PE(18:3(6Z,9Z,12Z)/P-18:1(9Z)) | C41H74NO7P | HBW |
| 6.4_756.5555 | 6.40 | 756.5555 | [M+CH3OH+H]+ | 723.5203 | 2 | PE(18:3(9Z,12Z,15Z)/P-18:1(11Z)) | C41H74NO7P | HBW |
| 6.4_756.5555 | 6.40 | 756.5555 | [M+CH3OH+H]+ | 723.5203 | 2 | PE(18:3(9Z,12Z,15Z)/P-18:1(9Z)) | C41H74NO7P | HBW |
| 6.4_756.5555 | 6.40 | 756.5555 | [M+CH3OH+H]+ | 723.5203 | 2 | PE(18:4(6Z,9Z,12Z,15Z)/P-18:0) | C41H74NO7P | HBW |
| 6.4_756.5555 | 6.40 | 756.5555 | [M+CH3OH+H]+ | 723.5203 | 2 | PE(20:4(5Z,8Z,11Z,14Z)/P-16:0) | C41H74NO7P | HBW |
| 6.4_756.5555 | 6.40 | 756.5555 | [M+CH3OH+H]+ | 723.5203 | 2 | PE(20:4(8Z,11Z,14Z,17Z)/P-16:0) | C41H74NO7P | HBW |
| 6.4_756.5555 | 6.40 | 756.5555 | [M+CH3OH+H]+ | 723.5203 | 2 | PE(P-16:0/20:4(5Z,8Z,11Z,14Z)) | C41H74NO7P | HBW |
| 6.4_756.5555 | 6.40 | 756.5555 | [M+CH3OH+H]+ | 723.5203 | 2 | PE(P-16:0/20:4(8Z,11Z,14Z,17Z)) | C41H74NO7P | HBW |
| 6.4_756.5555 | 6.40 | 756.5555 | [M+CH3OH+H]+ | 723.5203 | 2 | PE(P-18:0/18:4(6Z,9Z,12Z,15Z)) | C41H74NO7P | HBW |
| 6.4_756.5555 | 6.40 | 756.5555 | [M+CH3OH+H]+ | 723.5203 | 2 | PE(P-18:1(11Z)/18:3(6Z,9Z,12Z)) | C41H74NO7P | HBW |
| 6.4_756.5555 | 6.40 | 756.5555 | [M+CH3OH+H]+ | 723.5203 | 2 | PE(P-18:1(11Z)/18:3(9Z,12Z,15Z)) | C41H74NO7P | HBW |
| 6.4_756.5555 | 6.40 | 756.5555 | [M+CH3OH+H]+ | 723.5203 | 2 | PE(P-18:1(9Z)/18:3(6Z,9Z,12Z)) | C41H74NO7P | HBW |
| 6.4_756.5555 | 6.40 | 756.5555 | [M+CH3OH+H]+ | 723.5203 | 2 | PE(P-18:1(9Z)/18:3(9Z,12Z,15Z)) | C41H74NO7P | HBW |
| 6.4_756.5555 | 6.40 | 756.5555 | [M+Li]+ | 749.5359 | 4 | PE(20:4(5Z,8Z,11Z,14Z)/P-18:1(11Z)) | C43H76NO7P | HBW |
| 6.4_756.5555 | 6.40 | 756.5555 | [M+Li]+ | 749.5359 | 4 | PE(20:4(5Z,8Z,11Z,14Z)/P-18:1(9Z)) | C43H76NO7P | HBW |
| 6.4_756.5555 | 6.40 | 756.5555 | [M+Li]+ | 749.5359 | 4 | PE(20:4(8Z,11Z,14Z,17Z)/P-18:1(11Z)) | C43H76NO7P | HBW |
| 6.4_756.5555 | 6.40 | 756.5555 | [M+Li]+ | 749.5359 | 4 | PE(20:4(8Z,11Z,14Z,17Z)/P-18:1(9Z)) | C43H76NO7P | HBW |
| 6.4_756.5555 | 6.40 | 756.5555 | [M+Li]+ | 749.5359 | 4 | PE(20:5(5Z,8Z,11Z,14Z,17Z)/P-18:0) | C43H76NO7P | HBW |
| 6.4_756.5555 | 6.40 | 756.5555 | [M+Li]+ | 749.5359 | 4 | PE(22:5(4Z,7Z,10Z,13Z,16Z)/P-16:0) | C43H76NO7P | HBW |
| 6.4_756.5555 | 6.40 | 756.5555 | [M+Li]+ | 749.5359 | 4 | PE(22:5(7Z,10Z,13Z,16Z,19Z)/P-16:0) | C43H76NO7P | HBW |
| 6.4_756.5555 | 6.40 | 756.5555 | [M+Li]+ | 749.5359 | 4 | PE(P-16:0/22:5(4Z,7Z,10Z,13Z,16Z)) | C43H76NO7P | HBW |
| 6.4_756.5555 | 6.40 | 756.5555 | [M+Li]+ | 749.5359 | 4 | PE(P-16:0/22:5(7Z,10Z,13Z,16Z,19Z)) | C43H76NO7P | HBW |
| 6.4_756.5555 | 6.40 | 756.5555 | [M+Li]+ | 749.5359 | 4 | PE(P-18:0/20:5(5Z,8Z,11Z,14Z,17Z)) | C43H76NO7P | HBW |
| 6.4_756.5555 | 6.40 | 756.5555 | [M+Li]+ | 749.5359 | 4 | PE(P-18:1(11Z)/20:4(5Z,8Z,11Z,14Z)) | C43H76NO7P | HBW |
| 6.4_756.5555 | 6.40 | 756.5555 | [M+Li]+ | 749.5359 | 4 | PE(P-18:1(11Z)/20:4(8Z,11Z,14Z,17Z)) | C43H76NO7P | HBW |
| 6.4_756.5555 | 6.40 | 756.5555 | [M+Li]+ | 749.5359 | 4 | PE(P-18:1(9Z)/20:4(5Z,8Z,11Z,14Z)) | C43H76NO7P | HBW |
| 6.4_756.5555 | 6.40 | 756.5555 | [M+Li]+ | 749.5359 | 4 | PE(P-18:1(9Z)/20:4(8Z,11Z,14Z,17Z)) | C43H76NO7P | HBW |
| 6.4_756.5555 | 6.40 | 756.5555 | [M+Na]+ | 733.5621 | 5 | PE(17:0/18:0)[U] | C40H80NO8P | HBW |
| 6.4_756.5555 | 6.40 | 756.5555 | [M+Na]+ | 733.5621 | 5 | PE(19:0/16:0) | C40H80NO8P | HBW |
| 6.4_756.5555 | 6.40 | 756.5555 | [M+Na]+ | 733.5621 | 5 | PE(19:0/16:0)[U] | C40H80NO8P | HBW |
| 6.4_756.5555 | 6.40 | 756.5555 | [M+Na]+ | 733.5621 | 5 | PE(16:0/19:0) | C40H80NO8P | HBW |
| 6.4_756.5555 | 6.40 | 756.5555 | [M+Na]+ | 733.5621 | 5 | PE(16:0/19:0)[U] | C40H80NO8P | HBW |
| 6.4_756.5555 | 6.40 | 756.5555 | [M+Na]+ | 733.5621 | 5 | PE(20:0/15:0)[U] | C40H80NO8P | HBW |
| 6.4_756.5555 | 6.40 | 756.5555 | [M+Na]+ | 733.5621 | 5 | PE(18:0/17:0)[U] | C40H80NO8P | HBW |
| 6.4_756.5555 | 6.40 | 756.5555 | [M+Na]+ | 733.5621 | 5 | PE(13:0/22:0)[U] | C40H80NO8P | HBW |
| 6.4_756.5555 | 6.40 | 756.5555 | [M+Na]+ | 733.5621 | 5 | PE(14:0/21:0)[U] | C40H80NO8P | HBW |
| 6.4_756.5555 | 6.40 | 756.5555 | [M+Na]+ | 733.5621 | 5 | PE-NMe(17:0/17:0)[U] | C40H80NO8P | HBW |
| 6.4_756.5555 | 6.40 | 756.5555 | [M+Na]+ | 733.5622 | 5 | PC(10:0/22:0) | C40H80NO8P | HBW |
| 6.4_756.5555 | 6.40 | 756.5555 | [M+Na]+ | 733.5622 | 5 | PC(10:0/22:0)[U] | C40H80NO8P | HBW |
| 6.4_756.5555 | 6.40 | 756.5555 | [M+Na]+ | 733.5622 | 5 | PC(11:0/21:0) | C40H80NO8P | HBW |
| 6.4_756.5555 | 6.40 | 756.5555 | [M+Na]+ | 733.5622 | 5 | PC(12:0/20:0) | C40H80NO8P | HBW |
| 6.4_756.5555 | 6.40 | 756.5555 | [M+Na]+ | 733.5622 | 5 | PC(12:0/20:0)[U] | C40H80NO8P | HBW |
| 6.4_756.5555 | 6.40 | 756.5555 | [M+Na]+ | 733.5622 | 5 | PC(13:0/19:0) | C40H80NO8P | HBW |
| 6.4_756.5555 | 6.40 | 756.5555 | [M+Na]+ | 733.5622 | 5 | PC(13:0/19:0)[U] | C40H80NO8P | HBW |
| 6.4_756.5555 | 6.40 | 756.5555 | [M+Na]+ | 733.5622 | 5 | PC(14:0/18:0) | C40H80NO8P | HBW |
| 6.4_756.5555 | 6.40 | 756.5555 | [M+Na]+ | 733.5622 | 5 | PC(14:0/18:0)[U] | C40H80NO8P | HBW |
| 6.4_756.5555 | 6.40 | 756.5555 | [M+Na]+ | 733.5622 | 5 | PC(15:0/17:0) | C40H80NO8P | HBW |
| 6.4_756.5555 | 6.40 | 756.5555 | [M+Na]+ | 733.5622 | 5 | PC(15:0/17:0)[U] | C40H80NO8P | HBW |
| 6.4_756.5555 | 6.40 | 756.5555 | [M+Na]+ | 733.5622 | 5 | PC(16:0/16:0) | C40H80NO8P | HBW |
| 6.4_756.5555 | 6.40 | 756.5555 | [M+Na]+ | 733.5622 | 5 | PC(16:0/16:0)[U] | C40H80NO8P | HBW |
| 6.4_756.5555 | 6.40 | 756.5555 | [M+Na]+ | 733.5622 | 5 | PC(17:0/15:0) | C40H80NO8P | HBW |
| 6.4_756.5555 | 6.40 | 756.5555 | [M+Na]+ | 733.5622 | 5 | PC(17:0/15:0)[U] | C40H80NO8P | HBW |
| 6.4_756.5555 | 6.40 | 756.5555 | [M+Na]+ | 733.5622 | 5 | PC(18:0/14:0) | C40H80NO8P | HBW |
| 6.4_756.5555 | 6.40 | 756.5555 | [M+Na]+ | 733.5622 | 5 | PC(18:0/14:0)[U] | C40H80NO8P | HBW |
| 6.4_756.5555 | 6.40 | 756.5555 | [M+Na]+ | 733.5622 | 5 | PC(19:0/13:0) | C40H80NO8P | HBW |
| 6.4_756.5555 | 6.40 | 756.5555 | [M+Na]+ | 733.5622 | 5 | PC(20:0/12:0) | C40H80NO8P | HBW |
| 6.4_756.5555 | 6.40 | 756.5555 | [M+Na]+ | 733.5622 | 5 | PC(20:0/12:0)[U] | C40H80NO8P | HBW |
| 6.4_756.5555 | 6.40 | 756.5555 | [M+Na]+ | 733.5622 | 5 | PC(21:0/11:0) | C40H80NO8P | HBW |
| 6.4_756.5555 | 6.40 | 756.5555 | [M+Na]+ | 733.5622 | 5 | PC(22:0/10:0) | C40H80NO8P | HBW |
| 6.4_756.5555 | 6.40 | 756.5555 | [M+Na]+ | 733.5622 | 5 | PC(9:0/23:0) | C40H80NO8P | HBW |
| 6.4_756.5555 | 6.40 | 756.5555 | [M+Na]+ | 733.5622 | 5 | 1,2-dipalmitoyl-sn-glycero-3-PC | C40H80NO8P | HBW |
| 6.4_756.5555 | 6.40 | 756.5555 | [M+Na]+ | 733.5622 | 5 | PE(18:0(10(R)Me)/16:0) | C40H80NO8P | HBW |
| 6.4_756.5555 | 6.40 | 756.5555 | [M+Na]+ | 733.5622 | 5 | PC(14:0/18:0) | C40H80NO8P | HBW |
| 6.4_756.5555 | 6.40 | 756.5555 | [M+Na]+ | 733.5622 | 5 | PC(18:0/14:0) | C40H80NO8P | HBW |
| 6.4_756.5555 | 6.40 | 756.5555 | [M+Na]+ | 733.5622 | 5 | PE(15:0/20:0) | C40H80NO8P | HBW |
| 6.4_756.5555 | 6.40 | 756.5555 | [M+Na]+ | 733.5622 | 5 | PE(20:0/15:0) | C40H80NO8P | HBW |
| 6.4_756.5555 | 6.40 | 756.5555 | [M+CH3OH+H]+ | 723.5285 | 8 | Steroid derivative (structurally similar to) bacteriohopane-31,32,33,34-tetrol-35-cyclitol | C41H73NO9 | HBW |
| 6.9_760.4936 | 6.90 | 760.4936 | [M+H]+ | 759.4839 | 3 | PE(18:3(6Z,9Z,12Z)/20:5(5Z,8Z,11Z,14Z,17Z)) | C43H70NO8P | SBW |
| 6.9_760.4936 | 6.90 | 760.4936 | [M+H]+ | 759.4839 | 3 | PE(18:3(9Z,12Z,15Z)/20:5(5Z,8Z,11Z,14Z,17Z)) | C43H70NO8P | SBW |
| 6.9_760.4936 | 6.90 | 760.4936 | [M+H]+ | 759.4839 | 3 | PE(18:4(6Z,9Z,12Z,15Z)/20:4(5Z,8Z,11Z,14Z)) | C43H70NO8P | SBW |
| 6.9_760.4936 | 6.90 | 760.4936 | [M+H]+ | 759.4839 | 3 | PE(18:4(6Z,9Z,12Z,15Z)/20:4(8Z,11Z,14Z,17Z)) | C43H70NO8P | SBW |
| 6.9_760.4936 | 6.90 | 760.4936 | [M+H]+ | 759.4839 | 3 | PE(20:4(5Z,8Z,11Z,14Z)/18:4(6Z,9Z,12Z,15Z)) | C43H70NO8P | SBW |
| 6.9_760.4936 | 6.90 | 760.4936 | [M+H]+ | 759.4839 | 3 | PE(20:4(8Z,11Z,14Z,17Z)/18:4(6Z,9Z,12Z,15Z)) | C43H70NO8P | SBW |
| 6.9_760.4936 | 6.90 | 760.4936 | [M+H]+ | 759.4839 | 3 | PE(20:5(5Z,8Z,11Z,14Z,17Z)/18:3(6Z,9Z,12Z)) | C43H70NO8P | SBW |
| 6.9_760.4936 | 6.90 | 760.4936 | [M+H]+ | 759.4839 | 3 | PE(20:5(5Z,8Z,11Z,14Z,17Z)/18:3(9Z,12Z,15Z)) | C43H70NO8P | SBW |
| 6.9_760.4936 | 6.90 | 760.4936 | [M+Na]+ | 737.5081 | 4 | PS(17:0/14:1(9Z)) | C37H74N2O10P | SBW |
| 6.9_760.4936 | 6.90 | 760.4936 | [M+Na]+ | 737.4996 | 6 | PE(14:0/22:5(4Z,7Z,10Z,13Z,16Z)) | C41H72NO8P | SBW |
| 6.9_760.4936 | 6.90 | 760.4936 | [M+Na]+ | 737.4996 | 6 | PE(14:0/22:5(7Z,10Z,13Z,16Z,19Z)) | C41H72NO8P | SBW |
| 6.9_760.4936 | 6.90 | 760.4936 | [M+Na]+ | 737.4996 | 6 | PE(14:1(9Z)/22:4(7Z,10Z,13Z,16Z)) | C41H72NO8P | SBW |
| 6.9_760.4936 | 6.90 | 760.4936 | [M+Na]+ | 737.4996 | 6 | PE(16:0/20:5(5Z,8Z,11Z,14Z,17Z)) | C41H72NO8P | SBW |
| 6.9_760.4936 | 6.90 | 760.4936 | [M+Na]+ | 737.4996 | 6 | PE(16:1(9Z)/20:4(5Z,8Z,11Z,14Z)) | C41H72NO8P | SBW |
| 6.9_760.4936 | 6.90 | 760.4936 | [M+Na]+ | 737.4996 | 6 | PE(16:1(9Z)/20:4(8Z,11Z,14Z,17Z)) | C41H72NO8P | SBW |
| 6.9_760.4936 | 6.90 | 760.4936 | [M+Na]+ | 737.4996 | 6 | PE(18:1(11Z)/18:4(6Z,9Z,12Z,15Z)) | C41H72NO8P | SBW |
| 6.9_760.4936 | 6.90 | 760.4936 | [M+Na]+ | 737.4996 | 6 | PE(18:1(9Z)/18:4(6Z,9Z,12Z,15Z)) | C41H72NO8P | SBW |
| 6.9_760.4936 | 6.90 | 760.4936 | [M+Na]+ | 737.4996 | 6 | PE(18:2(9Z,12Z)/18:3(6Z,9Z,12Z)) | C41H72NO8P | SBW |
| 6.9_760.4936 | 6.90 | 760.4936 | [M+Na]+ | 737.4996 | 6 | PE(18:2(9Z,12Z)/18:3(9Z,12Z,15Z)) | C41H72NO8P | SBW |
| 6.9_760.4936 | 6.90 | 760.4936 | [M+Na]+ | 737.4996 | 6 | PE(18:3(6Z,9Z,12Z)/18:2(9Z,12Z)) | C41H72NO8P | SBW |
| 6.9_760.4936 | 6.90 | 760.4936 | [M+Na]+ | 737.4996 | 6 | PE(18:3(9Z,12Z,15Z)/18:2(9Z,12Z)) | C41H72NO8P | SBW |
| 6.9_760.4936 | 6.90 | 760.4936 | [M+Na]+ | 737.4996 | 6 | PE(18:4(6Z,9Z,12Z,15Z)/18:1(11Z)) | C41H72NO8P | SBW |
| 6.9_760.4936 | 6.90 | 760.4936 | [M+Na]+ | 737.4996 | 6 | PE(18:4(6Z,9Z,12Z,15Z)/18:1(9Z)) | C41H72NO8P | SBW |
| 6.9_760.4936 | 6.90 | 760.4936 | [M+Na]+ | 737.4996 | 6 | PE(20:4(5Z,8Z,11Z,14Z)/16:1(9Z)) | C41H72NO8P | SBW |
| 6.9_760.4936 | 6.90 | 760.4936 | [M+Na]+ | 737.4996 | 6 | PE(20:4(8Z,11Z,14Z,17Z)/16:1(9Z)) | C41H72NO8P | SBW |
| 6.9_760.4936 | 6.90 | 760.4936 | [M+Na]+ | 737.4996 | 6 | PE(20:5(5Z,8Z,11Z,14Z,17Z)/16:0) | C41H72NO8P | SBW |
| 6.9_760.4936 | 6.90 | 760.4936 | [M+Na]+ | 737.4996 | 6 | PE(22:4(7Z,10Z,13Z,16Z)/14:1(9Z)) | C41H72NO8P | SBW |
| 6.9_760.4936 | 6.90 | 760.4936 | [M+Na]+ | 737.4996 | 6 | PE(22:5(4Z,7Z,10Z,13Z,16Z)/14:0) | C41H72NO8P | SBW |
| 6.9_760.4936 | 6.90 | 760.4936 | [M+Na]+ | 737.4996 | 6 | PE(22:5(7Z,10Z,13Z,16Z,19Z)/14:0) | C41H72NO8P | SBW |
| 6.9_760.4936 | 6.90 | 760.4936 | [M+2Na-H]+ | 715.5152 | 9 | PE(16:0/18:2(9Z,12Z)) | C39H74NO8P | SBW |
| 6.9_760.4936 | 6.90 | 760.4936 | [M+2Na-H]+ | 715.5152 | 9 | 1-Palmitoyl-2-linoleoyl PE | C39H74NO8P | SBW |
| 6.9_760.4936 | 6.90 | 760.4936 | [M+2Na-H]+ | 715.5152 | 9 | PE(14:0/20:2(11Z,14Z)) | C39H74NO8P | SBW |
| 6.9_760.4936 | 6.90 | 760.4936 | [M+2Na-H]+ | 715.5152 | 9 | PE(14:1(9Z)/20:1(11Z)) | C39H74NO8P | SBW |
| 6.9_760.4936 | 6.90 | 760.4936 | [M+2Na-H]+ | 715.5152 | 9 | PE(16:1(9Z)/18:1(11Z)) | C39H74NO8P | SBW |
| 6.9_760.4936 | 6.90 | 760.4936 | [M+2Na-H]+ | 715.5152 | 9 | PE(16:1(9Z)/18:1(9Z)) | C39H74NO8P | SBW |
| 6.9_760.4936 | 6.90 | 760.4936 | [M+2Na-H]+ | 715.5152 | 9 | PE(18:1(11Z)/16:1(9Z)) | C39H74NO8P | SBW |
| 6.9_760.4936 | 6.90 | 760.4936 | [M+2Na-H]+ | 715.5152 | 9 | PE(18:1(9Z)/16:1(9Z)) | C39H74NO8P | SBW |
| 6.9_760.4936 | 6.90 | 760.4936 | [M+2Na-H]+ | 715.5152 | 9 | PE(18:2(9Z,12Z)/16:0) | C39H74NO8P | SBW |
| 6.9_760.4936 | 6.90 | 760.4936 | [M+2Na-H]+ | 715.5152 | 9 | PE(20:1(11Z)/14:1(9Z)) | C39H74NO8P | SBW |
| 6.9_760.4936 | 6.90 | 760.4936 | [M+2Na-H]+ | 715.5152 | 9 | PE(20:2(11Z,14Z)/14:0) | C39H74NO8P | SBW |
| 6.82_780.5501 | 6.82 | 780.5501 | [M+Na]+ | 757.5621 | 1 | PE-NMe(18:1(9E)/18:1(9E)) | C42H80NO8P | SBW |
| 6.82_780.5501 | 6.82 | 780.5501 | [M+Na]+ | 757.5621 | 1 | PE-NMe(18:1(9Z)/18:1(9Z))[U] | C42H80NO8P | SBW |
| 6.82_780.5501 | 6.82 | 780.5501 | [M+Na]+ | 757.5621 | 1 | PE-NMe(18:1(9Z)/18:1(9Z)) | C42H80NO8P | SBW |
| 6.82_780.5501 | 6.82 | 780.5501 | [M+Na]+ | 757.5621 | 1 | PE-NMe(18:1(9E)/18:1(9E))[U] | C42H80NO8P | SBW |
| 6.82_780.5501 | 6.82 | 780.5501 | [M+Na]+ | 757.5622 | 1 | PC(16:0/18:2(10E,12Z)) | C42H80NO8P | SBW |
| 6.82_780.5501 | 6.82 | 780.5501 | [M+Na]+ | 757.5622 | 1 | PC(16:0/18:2(11Z,13Z)) | C42H80NO8P | SBW |
| 6.82_780.5501 | 6.82 | 780.5501 | [M+Na]+ | 757.5622 | 1 | PC(16:0/18:2(2E,4E)) | C42H80NO8P | SBW |
| 6.82_780.5501 | 6.82 | 780.5501 | [M+Na]+ | 757.5622 | 1 | PC(16:0/18:2(2Z,4Z)) | C42H80NO8P | SBW |
| 6.82_780.5501 | 6.82 | 780.5501 | [M+Na]+ | 757.5622 | 1 | PC(16:0/18:2(6Z,9Z)) | C42H80NO8P | SBW |
| 6.82_780.5501 | 6.82 | 780.5501 | [M+Na]+ | 757.5622 | 1 | PC(16:0/18:2(9E,11E)) | C42H80NO8P | SBW |
| 6.82_780.5501 | 6.82 | 780.5501 | [M+Na]+ | 757.5622 | 1 | PC(16:0/18:2(9E,11Z)) | C42H80NO8P | SBW |
| 6.82_780.5501 | 6.82 | 780.5501 | [M+Na]+ | 757.5622 | 1 | PC(16:0/18:2(9E,12E)) | C42H80NO8P | SBW |
| 6.82_780.5501 | 6.82 | 780.5501 | [M+Na]+ | 757.5622 | 1 | PC(16:0/18:2(9E,12E))[U] | C42H80NO8P | SBW |
| 6.82_780.5501 | 6.82 | 780.5501 | [M+Na]+ | 757.5622 | 1 | PC(16:0/18:2(9Z,12Z)) | C42H80NO8P | SBW |
| 6.82_780.5501 | 6.82 | 780.5501 | [M+Na]+ | 757.5622 | 1 | PC(16:0/18:2(9Z,12Z))[S] | C42H80NO8P | SBW |
| 6.82_780.5501 | 6.82 | 780.5501 | [M+Na]+ | 757.5622 | 1 | PC(16:0/18:2(9Z,12Z))[U] | C42H80NO8P | SBW |
| 6.82_780.5501 | 6.82 | 780.5501 | [M+Na]+ | 757.5622 | 1 | PC(16:1(2Z)/18:1(9Z)) | C42H80NO8P | SBW |
| 6.82_780.5501 | 6.82 | 780.5501 | [M+Na]+ | 757.5622 | 1 | PC(16:1(7Z)/18:1(9Z))[U] | C42H80NO8P | SBW |
| 6.82_780.5501 | 6.82 | 780.5501 | [M+Na]+ | 757.5622 | 1 | PC(16:1(9Z)/18:1(11Z)) | C42H80NO8P | SBW |
| 6.82_780.5501 | 6.82 | 780.5501 | [M+Na]+ | 757.5622 | 1 | PC(16:1(9Z)/18:1(9Z)) | C42H80NO8P | SBW |
| 6.82_780.5501 | 6.82 | 780.5501 | [M+Na]+ | 757.5622 | 1 | PC(16:1(9Z)/18:1(9Z))[U] | C42H80NO8P | SBW |
| 6.82_780.5501 | 6.82 | 780.5501 | [M+Na]+ | 757.5622 | 1 | PC(17:1(10E)/17:1(10E))[U] | C42H80NO8P | SBW |
| 6.82_780.5501 | 6.82 | 780.5501 | [M+Na]+ | 757.5622 | 1 | PC(17:1(10Z)/17:1(10Z)) | C42H80NO8P | SBW |
| 6.82_780.5501 | 6.82 | 780.5501 | [M+Na]+ | 757.5622 | 1 | PC(17:1(9Z)/17:1(9Z)) | C42H80NO8P | SBW |
| 6.82_780.5501 | 6.82 | 780.5501 | [M+Na]+ | 757.5622 | 1 | PC(18:0/16:2(2E,4E)) | C42H80NO8P | SBW |
| 6.82_780.5501 | 6.82 | 780.5501 | [M+Na]+ | 757.5622 | 1 | PC(18:1(9Z)/16:1(3E))[U] | C42H80NO8P | SBW |
| 6.82_780.5501 | 6.82 | 780.5501 | [M+Na]+ | 757.5622 | 1 | PC(18:1(9Z)/16:1(9Z)) | C42H80NO8P | SBW |
| 6.82_780.5501 | 6.82 | 780.5501 | [M+Na]+ | 757.5622 | 1 | PC(18:2(2E,4E)/16:0) | C42H80NO8P | SBW |
| 6.82_780.5501 | 6.82 | 780.5501 | [M+Na]+ | 757.5622 | 1 | PC(18:2(6Z,9Z)/16:0) | C42H80NO8P | SBW |
| 6.82_780.5501 | 6.82 | 780.5501 | [M+Na]+ | 757.5622 | 1 | PC(18:2(9Z,12Z)/16:0) | C42H80NO8P | SBW |
| 6.82_780.5501 | 6.82 | 780.5501 | [M+Na]+ | 757.5622 | 1 | PC(18:2(9Z,12Z)/16:0)[U] | C42H80NO8P | SBW |
| 6.82_780.5501 | 6.82 | 780.5501 | [M+Na]+ | 757.5622 | 1 | PC(14:0/20:2(11Z,14Z)) | C42H80NO8P | SBW |
| 6.82_780.5501 | 6.82 | 780.5501 | [M+Na]+ | 757.5622 | 1 | PC(14:1(9Z)/20:1(11Z)) | C42H80NO8P | SBW |
| 6.82_780.5501 | 6.82 | 780.5501 | [M+Na]+ | 757.5622 | 1 | PC(16:0/18:2(9Z,12Z)) | C42H80NO8P | SBW |
| 6.82_780.5501 | 6.82 | 780.5501 | [M+Na]+ | 757.5622 | 1 | PC(16:1(9Z)/18:1(11Z)) | C42H80NO8P | SBW |
| 6.82_780.5501 | 6.82 | 780.5501 | [M+Na]+ | 757.5622 | 1 | PC(16:1(9Z)/18:1(9Z)) | C42H80NO8P | SBW |
| 6.82_780.5501 | 6.82 | 780.5501 | [M+Na]+ | 757.5622 | 1 | PC(18:1(11Z)/16:1(9Z)) | C42H80NO8P | SBW |
| 6.82_780.5501 | 6.82 | 780.5501 | [M+Na]+ | 757.5622 | 1 | PC(18:1(9Z)/16:1(9Z)) | C42H80NO8P | SBW |
| 6.82_780.5501 | 6.82 | 780.5501 | [M+Na]+ | 757.5622 | 1 | PC(18:2(9Z,12Z)/16:0) | C42H80NO8P | SBW |
| 6.82_780.5501 | 6.82 | 780.5501 | [M+Na]+ | 757.5622 | 1 | PC(20:1(11Z)/14:1(9Z)) | C42H80NO8P | SBW |
| 6.82_780.5501 | 6.82 | 780.5501 | [M+Na]+ | 757.5622 | 1 | PC(20:2(11Z,14Z)/14:0) | C42H80NO8P | SBW |
| 6.82_780.5501 | 6.82 | 780.5501 | [M+Na]+ | 757.5622 | 1 | PE(15:0/22:2(13Z,16Z)) | C42H80NO8P | SBW |
| 6.82_780.5501 | 6.82 | 780.5501 | [M+Na]+ | 757.5622 | 1 | PE(22:2(13Z,16Z)/15:0) | C42H80NO8P | SBW |
| 6.82_780.5501 | 6.82 | 780.5501 | [M+Li]+ | 773.5359 | 2 | 1-(8-[3]-ladderane-octanoyl)-2-(8-[3]-ladderane-octanyl)-sn-glycerophosphoethanolamine | C45H76NO7P | SBW |
| 6.82_780.5501 | 6.82 | 780.5501 | [M+Li]+ | 773.5359 | 2 | PE(22:6(4Z,7Z,10Z,13Z,16Z,19Z)/P-18:1(11Z)) | C45H76NO7P | SBW |
| 6.82_780.5501 | 6.82 | 780.5501 | [M+Li]+ | 773.5359 | 2 | PE(22:6(4Z,7Z,10Z,13Z,16Z,19Z)/P-18:1(9Z)) | C45H76NO7P | SBW |
| 6.82_780.5501 | 6.82 | 780.5501 | [M+Li]+ | 773.5359 | 2 | PE(P-18:1(11Z)/22:6(4Z,7Z,10Z,13Z,16Z,19Z)) | C45H76NO7P | SBW |
| 6.82_780.5501 | 6.82 | 780.5501 | [M+Li]+ | 773.5359 | 2 | PE(P-18:1(9Z)/22:6(4Z,7Z,10Z,13Z,16Z,19Z)) | C45H76NO7P | SBW |
| 6.82_780.5501 | 6.82 | 780.5501 | [M+CH3OH+H]+ | 747.5203 | 4 | PE(P-16:0/22:6(4Z,7Z,10Z,13Z,16Z,19Z)) | C43H74NO7P | SBW |
| 6.82_780.5501 | 6.82 | 780.5501 | [M+H]+ | 779.5465 | 4 | PC(16:0/20:5(5E,8E,11E,14E,17E))[U] | C44H78NO8P | SBW |
| 6.82_780.5501 | 6.82 | 780.5501 | [M+H]+ | 779.5465 | 4 | PC(16:0/20:5(5Z,8Z,11Z,14Z,17Z)) | C44H78NO8P | SBW |
| 6.82_780.5501 | 6.82 | 780.5501 | [M+H]+ | 779.5465 | 4 | PC(16:0/20:5(5Z,8Z,11Z,14Z,17Z))[U] | C44H78NO8P | SBW |
| 6.82_780.5501 | 6.82 | 780.5501 | [M+H]+ | 779.5465 | 4 | PC(16:1(9Z)/20:4(5Z,8Z,11Z,14Z))[U] | C44H78NO8P | SBW |
| 6.82_780.5501 | 6.82 | 780.5501 | [M+H]+ | 779.5465 | 4 | PC(16:1(9Z)/20:4(5Z,8Z,11Z,14Z)) | C44H78NO8P | SBW |
| 6.82_780.5501 | 6.82 | 780.5501 | [M+H]+ | 779.5465 | 4 | PC(18:4(2E,4E,6E,11Z)/18:1(11Z))[U] | C44H78NO8P | SBW |
| 6.82_780.5501 | 6.82 | 780.5501 | [M+H]+ | 779.5465 | 4 | PC(16:1(7Z)/20:4(5Z,8Z,11Z,14Z)) | C44H78NO8P | SBW |
| 6.82_780.5501 | 6.82 | 780.5501 | [M+H]+ | 779.5465 | 4 | PC(14:0/22:5(4Z,7Z,10Z,13Z,16Z)) | C44H78NO8P | SBW |
| 6.82_780.5501 | 6.82 | 780.5501 | [M+H]+ | 779.5465 | 4 | PC(14:0/22:5(7Z,10Z,13Z,16Z,19Z)) | C44H78NO8P | SBW |
| 6.82_780.5501 | 6.82 | 780.5501 | [M+H]+ | 779.5465 | 4 | PC(14:1(9Z)/22:4(7Z,10Z,13Z,16Z)) | C44H78NO8P | SBW |
| 6.82_780.5501 | 6.82 | 780.5501 | [M+H]+ | 779.5465 | 4 | PC(16:0/20:5(5Z,8Z,11Z,14Z,17Z)) | C44H78NO8P | SBW |
| 6.82_780.5501 | 6.82 | 780.5501 | [M+H]+ | 779.5465 | 4 | PC(16:1(9Z)/20:4(5Z,8Z,11Z,14Z)) | C44H78NO8P | SBW |
| 6.82_780.5501 | 6.82 | 780.5501 | [M+H]+ | 779.5465 | 4 | PC(16:1(9Z)/20:4(8Z,11Z,14Z,17Z)) | C44H78NO8P | SBW |
| 6.82_780.5501 | 6.82 | 780.5501 | [M+H]+ | 779.5465 | 4 | PC(18:1(11Z)/18:4(6Z,9Z,12Z,15Z)) | C44H78NO8P | SBW |
| 6.82_780.5501 | 6.82 | 780.5501 | [M+H]+ | 779.5465 | 4 | PC(18:1(9Z)/18:4(6Z,9Z,12Z,15Z)) | C44H78NO8P | SBW |
| 6.82_780.5501 | 6.82 | 780.5501 | [M+H]+ | 779.5465 | 4 | PC(18:2(9Z,12Z)/18:3(6Z,9Z,12Z)) | C44H78NO8P | SBW |
| 6.82_780.5501 | 6.82 | 780.5501 | [M+H]+ | 779.5465 | 4 | PC(18:2(9Z,12Z)/18:3(9Z,12Z,15Z)) | C44H78NO8P | SBW |
| 6.82_780.5501 | 6.82 | 780.5501 | [M+H]+ | 779.5465 | 4 | PC(18:3(6Z,9Z,12Z)/18:2(9Z,12Z)) | C44H78NO8P | SBW |
| 6.82_780.5501 | 6.82 | 780.5501 | [M+H]+ | 779.5465 | 4 | PC(18:3(9Z,12Z,15Z)/18:2(9Z,12Z)) | C44H78NO8P | SBW |
| 6.82_780.5501 | 6.82 | 780.5501 | [M+H]+ | 779.5465 | 4 | PC(18:4(6Z,9Z,12Z,15Z)/18:1(11Z)) | C44H78NO8P | SBW |
| 6.82_780.5501 | 6.82 | 780.5501 | [M+H]+ | 779.5465 | 4 | PC(18:4(6Z,9Z,12Z,15Z)/18:1(9Z)) | C44H78NO8P | SBW |
| 6.82_780.5501 | 6.82 | 780.5501 | [M+H]+ | 779.5465 | 4 | PC(20:4(5Z,8Z,11Z,14Z)/16:1(9Z)) | C44H78NO8P | SBW |
| 6.82_780.5501 | 6.82 | 780.5501 | [M+H]+ | 779.5465 | 4 | PC(20:4(8Z,11Z,14Z,17Z)/16:1(9Z)) | C44H78NO8P | SBW |
| 6.82_780.5501 | 6.82 | 780.5501 | [M+H]+ | 779.5465 | 4 | PC(20:5(5Z,8Z,11Z,14Z,17Z)/16:0) | C44H78NO8P | SBW |
| 6.82_780.5501 | 6.82 | 780.5501 | [M+H]+ | 779.5465 | 4 | PC(22:4(7Z,10Z,13Z,16Z)/14:1(9Z)) | C44H78NO8P | SBW |
| 6.82_780.5501 | 6.82 | 780.5501 | [M+H]+ | 779.5465 | 4 | PC(22:5(4Z,7Z,10Z,13Z,16Z)/14:0) | C44H78NO8P | SBW |
| 6.82_780.5501 | 6.82 | 780.5501 | [M+H]+ | 779.5465 | 4 | PC(22:5(7Z,10Z,13Z,16Z,19Z)/14:0) | C44H78NO8P | SBW |
| 6.82_780.5501 | 6.82 | 780.5501 | [M+CH3OH+H]+ | 747.5203 | 4 | PE(20:5(5Z,8Z,11Z,14Z,17Z)/P-18:1(11Z)) | C43H74NO7P | SBW |
| 6.82_780.5501 | 6.82 | 780.5501 | [M+CH3OH+H]+ | 747.5203 | 4 | PE(20:5(5Z,8Z,11Z,14Z,17Z)/P-18:1(9Z)) | C43H74NO7P | SBW |
| 6.82_780.5501 | 6.82 | 780.5501 | [M+CH3OH+H]+ | 747.5203 | 4 | PE(22:6(4Z,7Z,10Z,13Z,16Z,19Z)/P-16:0) | C43H74NO7P | SBW |
| 6.82_780.5501 | 6.82 | 780.5501 | [M+CH3OH+H]+ | 747.5203 | 4 | PE(P-18:1(11Z)/20:5(5Z,8Z,11Z,14Z,17Z)) | C43H74NO7P | SBW |
| 6.82_780.5501 | 6.82 | 780.5501 | [M+CH3OH+H]+ | 747.5203 | 4 | PE(P-18:1(9Z)/20:5(5Z,8Z,11Z,14Z,17Z)) | C43H74NO7P | SBW |
| 6.82_780.5501 | 6.82 | 780.5501 | [M+H-2H2O]+ | 815.5551 | 9 | PS(17:0/20:4(5Z,8Z,11Z,14Z)) | C43H80N2O10P | SBW |
| 7.07_782.536 | 7.07 | 782.5360 | [M+CH3OH+H]+ | 749.4996 | 3 | PC(12:0/22:6(4Z,7Z,10Z,13Z,16Z,19Z)) | C42H72NO8P | SBW |
| 7.07_782.536 | 7.07 | 782.5360 | [M+CH3OH+H]+ | 749.4996 | 3 | PC(14:1(9Z)/20:5(5Z,8Z,11Z,14Z,17Z)) | C42H72NO8P | SBW |
| 7.07_782.536 | 7.07 | 782.5360 | [M+CH3OH+H]+ | 749.4996 | 3 | PC(20:5(5Z,8Z,11Z,14Z,17Z)/14:1(9Z)) | C42H72NO8P | SBW |
| 7.07_782.536 | 7.07 | 782.5360 | [M+CH3OH+H]+ | 749.4996 | 3 | PE(15:0/22:6(4Z,7Z,10Z,13Z,16Z,19Z)) | C42H72NO8P | SBW |
| 7.07_782.536 | 7.07 | 782.5360 | [M+CH3OH+H]+ | 749.4996 | 3 | PE(22:6(4Z,7Z,10Z,13Z,16Z,19Z)/15:0) | C42H72NO8P | SBW |
| 7.07_782.536 | 7.07 | 782.5360 | [M+Li]+ | 775.5152 | 6 | PC(14:1(9Z)/22:6(4Z,7Z,10Z,13Z,16Z,19Z)) | C44H74NO8P | SBW |
| 7.07_782.536 | 7.07 | 782.5360 | [M+Li]+ | 775.5152 | 6 | PC(18:3(6Z,9Z,12Z)/18:4(6Z,9Z,12Z,15Z)) | C44H74NO8P | SBW |
| 7.07_782.536 | 7.07 | 782.5360 | [M+Li]+ | 775.5152 | 6 | PC(18:3(9Z,12Z,15Z)/18:4(6Z,9Z,12Z,15Z)) | C44H74NO8P | SBW |
| 7.07_782.536 | 7.07 | 782.5360 | [M+Li]+ | 775.5152 | 6 | PC(18:4(6Z,9Z,12Z,15Z)/18:3(6Z,9Z,12Z)) | C44H74NO8P | SBW |
| 7.07_782.536 | 7.07 | 782.5360 | [M+Li]+ | 775.5152 | 6 | PC(18:4(6Z,9Z,12Z,15Z)/18:3(9Z,12Z,15Z)) | C44H74NO8P | SBW |
| 7.07_782.536 | 7.07 | 782.5360 | [M+Li]+ | 775.5152 | 6 | PC(22:6(4Z,7Z,10Z,13Z,16Z,19Z)/14:1(9Z)) | C44H74NO8P | SBW |
| 0.57_783.2171 | 0.57 | 783.2171 | [M+H-H2O]+ | 800.2164 | 4 | Tibouchinin | C38H40O19 | SBW |
| 0.57_783.2171 | 0.57 | 783.2171 | [M+H-H2O]+ | 800.2164 | 4 | Malvidin 3-O-(6-O-(Z)-p-coumalonyl-beta-glucopyranoside)-5-O-beta-glucopyranoside | C38H40O19 | SBW |
| 0.57_783.2171 | 0.57 | 783.2171 | [M+H-H2O]+ | 800.2164 | 4 | Malvidin 3-O-(6-O-(4-O-caffeoyl-alpha-rhamnopyranosyl)-beta-glucopyranoside) | C38H40O19 | SBW |
| 0.57_783.2171 | 0.57 | 783.2171 | [M+H-H2O]+ | 800.2164 | 4 | Isoscoparin 2\'\'-(6-(E)-ferulylglucoside) | C38H40O19 | SBW |
| 0.57_783.2171 | 0.57 | 783.2171 | [M+H-H2O]+ | 800.2164 | 4 | Isorhamnetin 3-(3\'\'\'-ferulylrobinobioside) | C38H40O19 | SBW |
| 0.57_783.2171 | 0.57 | 783.2171 | [M+H]+ | 782.2058 | 5 | Pelargonidin 3-(6\'\'-p-coumarylglucoside)-5-(6\'\'\'-acetylglucoside) | C38H38O18 | SBW |
| 0.57_783.2171 | 0.57 | 783.2171 | [M+H]+ | 782.2058 | 5 | Pelargonidin 3-(6\'\'-p-coumarylglucoside)-5-(6\'\'\'-acetylglucoside) | C38H38O18 | SBW |
| 7.02_937.5846 | 7.02 | 937.5846 | [M+H]+ | 936.5810 | 3 | 1,2-Di-(9Z,12Z,15Z-octadecatrienoyl)-3-(Galactosyl-alpha-1-6-Galactosyl-beta-1)-glycerol | C51H84O15 | SBW |
| 7.02_937.5846 | 7.02 | 937.5846 | [M+Na]+ | 914.5884 | 7 | PI(18:0/22:4(10Z,13Z,16Z,19Z)) | C49H87O13P | SBW |
| 7.02_937.5846 | 7.02 | 937.5846 | [M+Na]+ | 914.5884 | 7 | PI(18:0/22:4(7Z,10Z,13Z,16Z)) | C49H87O13P | SBW |
| 7.02_937.5846 | 7.02 | 937.5846 | [M+Na]+ | 914.5884 | 7 | PI(18:2(9Z,12Z)/22:2(13Z,16Z)) | C49H87O13P | SBW |
| 7.02_937.5846 | 7.02 | 937.5846 | [M+Na]+ | 914.5884 | 7 | PI(20:0/20:3(8Z,11Z,14Z)) | C49H87O13P | SBW |
| 7.02_937.5846 | 7.02 | 937.5846 | [M+Na]+ | 914.5884 | 7 | PI(20:0/20:4(5Z,8Z,11Z,14Z)) | C49H87O13P | SBW |
| 7.02_937.5846 | 7.02 | 937.5846 | [M+Na]+ | 914.5884 | 7 | PI(20:2(11Z,14Z)/20:2(11Z,14Z)) | C49H87O13P | SBW |
| 7.02_937.5846 | 7.02 | 937.5846 | [M+Na]+ | 914.5884 | 7 | PI(20:4(5Z,8Z,11Z,14Z)/20:0) | C49H87O13P | SBW |
| 7.02_937.5846 | 7.02 | 937.5846 | [M+Na]+ | 914.5884 | 7 | PI(20:4(8Z,11Z,14Z,17Z)/20:0) | C49H87O13P | SBW |
| 7.02_937.5846 | 7.02 | 937.5846 | [M+Na]+ | 914.5884 | 7 | PI(22:2(13Z,16Z)/18:2(9Z,12Z)) | C49H87O13P | SBW |
| 7.02_937.5846 | 7.02 | 937.5846 | [M+Na]+ | 914.5884 | 7 | PI(22:4(10Z,13Z,16Z,19Z)/18:0) | C49H87O13P | SBW |
| 7.02_937.5846 | 7.02 | 937.5846 | [M+Na]+ | 914.5884 | 7 | PI(22:4(7Z,10Z,13Z,16Z)/18:0) | C49H87O13P | SBW |
| 7.04_938.5852 | 7.04 | 938.5852 | NC |  |  |  |  | SBW |
| 6.83_961.5853 | 6.83 | 961.5853 | [M+H]+ | 960.5769 | 1 | Polyketide derivative (structurally similar to Megalomicin C1) | C48H84N2O17 | SBW |
| 6.83_962.5886 | 6.83 | 962.5886 | NC |  |  |  |  | SBW |
| 3.94_1039.664 | 3.94 | 1039.664 | [M+2Na-H]+ | 994.6944 | 1 | Dodecaprenyl diphosphate | C60H100O7P2 | HBW |
